# Effect of engineered mesoporous silica particles with tailored pore size on glycaemic control in individuals with prediabetes or type 2 diabetes: a randomised, double-blind, placebo-controlled SHINE trial

**DOI:** 10.1016/j.eclinm.2026.104042

**Published:** 2026-07-02

**Authors:** Jeanha Baek, Anna Ioannidou, Melissa L. Borg, Ghislaine Robert-Nicoud, Kirsi H. Pietiläinen, Stephan Rössner, Eric V. Johnston, Tore Bengtsson

**Affiliations:** a Sigrid Therapeutics AB, Stockholm, Sweden; b Obesity Research Unit, Research Program for Clinical and Molecular Metabolism, Faculty of Medicine, University of Helsinki, Finland; c Healthy Weight Hub, Abdominal Center, Helsinki University Hospital and University of Helsinki, Finland; d Apple Bay Obesity Research Centre, Stockholm, Sweden; e Department of Molecular Biosciences, The Wenner-Gren Institute, Stockholm University, Stockholm, Sweden

**Keywords:** Mesoporous silica, Prediabetes, Type 2 diabetes, Body weight, Glycaemic control

## Abstract

**Background:**

Prediabetes is a major global health concern due to its high prevalence and strong association with obesity and increased cardiovascular risk. Most individuals with prediabetes progress to type 2 diabetes, and although lifestyle modification is recommended early, long-term adherence and scalability remain challenging. SiPore21 offers a promising solution. SiPore21 is an oral gel containing engineered mesoporous silica particles that act locally in the gut by entrapping digestive enzymes and modulating macronutrient uptake.

**Methods:**

This 12-week, randomised, double-blind, placebo-controlled, multicentre trial was conducted at 27 centres across three European countries (ClinicalTrials.gov: NCT06087822). During Oct 5, 2023 and Jul 30, 2024, 318 participants were enrolled and assessed, and 312 completed the intervention (SiPore21 n = 155; placebo n = 157). Adults aged 18–70 years with overweight or obesity (BMI > 25 to ≤40 kg/m^2^) and elevated HbA1c (≥42 and ≤58 mmol/mol [≥6 and ≤ 7.5%]) were eligible. Participants were randomly assigned 1:1 to SiPore21 or placebo using a web-based interactive randomisation system. SiPore21 was administered three times daily with meals for 12 weeks. The primary outcome was change in HbA1c from baseline to Week 12. Secondary outcomes included changes in body weight, lipid profiles, and other metabolic parameters. Safety and tolerability were assessed throughout. The primary analysis was performed on all participants who received at least one dose and at least one post-baseline HbA1c measurement; the safety analysis included all participants who received at least one dose.

**Findings:**

SiPore21 significantly reduced HbA1c from baseline (p = 0.0036), indicating a favourable glycaemic effect, whereas no significant reduction was observed with placebo (p = 0.0872). In a post hoc sex-stratified analysis, the HbA1c reduction vs. placebo was statistically significant in women (p = 0.019), whereas a marked placebo response in men attenuated the overall between-group difference. SiPore21 also improved body weight (p = 0.0374), fat mass (p = 0.05), and lipid measures (LDL-C; p = 0.035, total cholesterol; p = 0.0496) compared to placebo, and was associated with reduced progression from prediabetes to diabetes (p = 0.0233) and increased reversion to earlier glycaemic states (p = 0.0044). SiPore21 was safe and well tolerated, with only mild, transient gastrointestinal events and no serious adverse device effects.

**Interpretation:**

SiPore21 improved glycaemic control, body composition, and lipid metabolism while stabilising glycaemic status, whereas corresponding improvements were not observed in the placebo group. SiPore21 was well tolerated, with no clinically meaningful safety concerns identified. These findings support its potential as a safe and effective non-pharmacological option for prediabetes or early type 2 diabetes.

**Funding:**

Sigrid Therapeutics AB.

**Research in context**

**Evidence before this study**

We searched PubMed from January 2010 to February 2025 using the terms “mesoporous silica”, “glycaemic control”, “lifestyle intervention’, “prediabetes”, and “type 2 diabetes prevention”. Preclinical studies showed that engineered mesoporous silica particles act as a mechanical sieve in the gastrointestinal tract by physically separating digestive enzymes from undigested or partially digested food, thereby slowing macronutrient breakdown and reducing macronutrient absorption. Prior clinical studies demonstrated that ingestion of these silica particles is safe and confirmed the intended mode of action in humans. The largest intervention trial in prediabetes to date, the Diabetes Prevention Program, showed that intensive lifestyle intervention and, to a lesser extent, pharmacological therapy can delay diabetes onset. Promoting lifestyle change is the primary recommendation for individuals with prediabetes and remains the foundation of early type 2 diabetes management before pharmacological therapy is initiated. However, lifestyle-based approaches are resource-intensive and associated with poor long-term adherence. Together, this evidence highlighted the need for safe, effective, and scalable non-pharmacological strategies to support metabolic health and delay diabetes progression.

**Added value of this study**

This multicentre trial provides, to our knowledge, the first randomised clinical evidence that a non-pharmacological, orally administered silica-based intervention can beneficially influence glycaemic control, body composition, and metabolic health in individuals with prediabetes or type 2 diabetes. SiPore21, an oral gel containing engineered mesoporous silica particles, is designed to address metabolic dysregulation without systemic exposure. Over twelve weeks, use of SiPore21 was associated with improved markers of glycaemic control, reductions in body weight, favourable changes in lipid metabolism, and higher rates of reversion to earlier glycaemic states, all accompanied by excellent safety and adherence. These findings build on earlier preclinical and clinical work and demonstrate the potential clinical relevance and scalability of this approach.

**Implications of all the available evidence**

This study provides the strongest clinical evidence to date supporting the use of mesoporous silica particles as a novel, non-pharmacological approach to improve glycaemic control and metabolic health. Acting locally in the gastrointestinal tract without systemic absorption, SiPore21 may serve as a complementary approach alongside lifestyle modification and could help delay the need for pharmacological therapy for individuals with prediabetes or early type 2 diabetes. Future research is warranted to assess the long-term efficacy of SiPore21 and its applicability in broader populations, including those with established type 2 diabetes.

## Introduction

SiPore21 is an oral gel containing engineered mesoporous silica particles. These particles are micron-sized and tailored to have pores with a specific diameter, which are slightly larger than the size of digestive enzymes but smaller than undigested food. Owing to this unique physical structure and the consequent diffusion of enzymes into the pores, SiPore21 functions as a molecular sieve that physically separates digestive enzymes from undigested or partially digested food in the gastrointestinal tract. This physical separation reduces the enzymatic breakdown of carbohydrates and fats, thereby lowering nutrient absorption.^1^^,^^2^ Consistent with this mode of action, mesoporous silica particles with precisely engineered pore sizes have demonstrated selective enzyme sequestration *in vitro*, *ex vivo*, and *in vivo* settings, resulting in reduced carbohydrate and lipid digestion. These positive effects were pore size-dependent and were not observed in particles with pores smaller than the enzymes, supporting a size-selective mode of action rather than non-specific adsorption.^2^ The downstream biological effects of this mode of action have been demonstrated in several preclinical and clinical studies using SiPore21 prototypes. In diet-induced obese mice, SiPore21 prototype treatment significantly reduced body weight and fat composition, suppressed further weight gain, and improved glucose tolerance.^3^^,^^4^ In the first-in-human trial, 12 weeks of treatment was safe and significantly lowered HbA1c and low-density lipoprotein cholesterol in individuals with obesity.^2^^,^^5^ A subsequent proof-of-concept study confirmed these findings in individuals with prediabetes and early type 2 diabetes (T2D), showing clinically meaningful HbA1c reductions and improvements in several metabolic parameters with minimal adverse effects.^6^

These findings demonstrate the safety and metabolic benefits of SiPore21 prototypes and underscore the potential of SiPore21 as a novel, non-pharmacological approach to address one of today’s most pressing metabolic health challenges, the prevention and early management of T2D. Prediabetes, or intermediate hyperglycaemia, is characterised by blood glucose above normal but below the diagnostic threshold for T2D. It represents a major global health concern due to its high prevalence and strong association with obesity, insulin resistance, and increased cardiovascular risk.^7^ Approximately 25% of individuals with prediabetes progress to T2D within three to five years, and up to 70% will do so over their lifetime.^8^ Despite its clinical importance, management options remain limited.

Current guidelines for prediabetes primarily recommend lifestyle interventions, such as dietary modification and increased physical activity. Similarly, for early T2D, promoting lifestyle change remains the foundation of disease management before pharmacological therapy is initiated.^7^^,^^9^ However, these strategies are resource-intensive, challenging to implement in real-world settings, and often associated with poor long-term adherence.^10^^,^^11^ Pharmacological options are also limited: while metformin is sometimes prescribed to individuals at high risk of developing T2D, its broader use in prediabetes is constrained by tolerability issues and the absence of a formal indication.^12^^,^^13^

Given these challenges, there is a pressing need for safe, effective, and scalable non-pharmacological strategies to prevent disease progression and support metabolic health. In this context, SiPore21 represents a novel, locally acting intervention that acts within the gastrointestinal tract without systemic exposure, offering a potential adjunct or complementary approach to existing lifestyle-based strategies. The present clinical study, SHINE (SiPore Halts Intermediate Hyperglycaemia), was therefore designed to evaluate the safety and efficacy of SiPore21 in individuals with overweight or obesity, diagnosed with prediabetes or early T2D.

## Methods

### Study design

The SHINE study was a 12-week, randomised, double-blind, placebo-controlled, multicenter trial conducted at 27 centres across three European countries (Poland, Romania, Slovakia). The study aimed to evaluate the efficacy and safety of SiPore21 in adults with overweight or obesity and elevated HbA1c levels.

### Ethics

The study adhered to the Medical Device Regulation (EU) 2017/745, ISO 14155:2020, the principles of Good Clinical Practice (GCP), applicable local regulations, and the ethical principles outlined in the Declaration of Helsinki. The trial is registered at ClinicalTrials.gov (identifier: NCT06087822; URL https://clinicaltrials.gov/study/NCT06087822) on 11 October 2023. The first participant was enrolled on 5 October 2023; the short interval between enrolment and public registration reflects the registry’s administrative processing period. The study protocol and statistical analysis plan were finalised prior to enrolment, and no changes were made during this interval. Ethics committee and regulatory approvals were obtained in each participating country before study initiation, including approval from the Bioethics Committee at the District Medical Chamber in Lublin (Poland; approval no. 93/2023/KB/IX), the National Bioethics Committee for Medicines and Medical Devices (Romania; approval no. 7DM/08.05.2023), and the Etická komisia Trencianskeho samosprávneho kraja (Slovakia; protocol no. SITH/001921). All participants provided written informed consent prior to enrolment. The detailed study protocol and statistical analysis plan is available in the Supplementary Material.

### Participants

A total of 318 participants were enrolled. Eligible participants were aged 18–70 years with overweight or obesity (BMI > 25 to ≤ 40 kg/m^2^) and elevated HbA1c levels (≥42 and ≤ 58 mmol/mol [≥6 and ≤ 7.5%]), and were instructed and agreed to maintain their usual diet and level of physical activity throughout the study. Key exclusion criteria included: type 1 diabetes, recent cardiovascular events, uncontrolled hypertension, gastrointestinal disease, major abdominal surgery, abnormal ECG or laboratory values at screening, recent use of weight loss or antidiabetic medications, extreme diets, high sugar intake, or recent blood loss or transfusion. A full list of eligibility criteria is provided in the study protocol (Supplementary Material).

### Randomisation and masking

Participants were randomly assigned (1:1) to receive either SiPore21 or placebo for 12 weeks using a web-based interactive randomisation system (IWRS). Stratified randomisation was employed, with stratification by baseline HbA1c (<48 vs. ≥48 mmol/mol [<6.5 vs. ≥6.5%]) and BMI (<30 vs. ≥30 kg/m^2^). SiPore21 and placebo were comparable in appearance, taste, texture, and smell and indistinguishable in packaging and labelling. Participants, investigators, site staff, and statisticians were blinded to group allocation throughout the trial. Emergency unblinding was implemented in the randomisation system and it was strictly restricted to authorised persons at participating study sites.

### Procedures and assessments

Participants received SiPore21 or a matching placebo for 12 weeks. Each dose of SiPore21 contained three grams of engineered mesoporous silica particles and was administered orally three times daily with the main meals (breakfast, lunch, and dinner), corresponding to a total daily dose of nine grams. Participants were instructed to take each dose in connection with a meal throughout the intervention period.

Participants attended clinic visits at baseline, Week 6, and Week 12, with interim phone calls on Week 3 and Week 9, and two post-treatment phone follow-ups at Week 13 and the other between Week 15–17. At each clinic visit, body weight, blood pressure and heart rate were measured, and fasting blood samples were collected for analysis of HbA1c, glucose, insulin and lipid parameters. Anthropometric assessments included body weight, body mass index (BMI), waist circumference (WC), and sagittal abdominal diameter (SAD). Body composition was assessed via bioelectrical impedance analysis (BIA). Safety parameters, including blood count, liver and renal function parameters, vitamins (B12, D), and trace elements (Mg, Zn) were evaluated at baseline and Week 12. Participants also completed the 12-Item Short Form Health Survey (SF-12) at these time points. Adverse events were documented at all participant contacts. A full list of assessed parameters is provided in the study protocol (Supplementary Material).

### Outcomes

The primary endpoint was the change in HbA1c from baseline to Week 12, compared between the SiPore21 and placebo groups. The main secondary endpoint was the change in body weight over the same period. Additional secondary endpoints included changes in HOMA-IR, fasting blood glucose and insulin levels, lipid parameters (low density lipoprotein cholesterol (LDL-C), total cholesterol, triglycerides), body composition parameters (fat mass, fat-free mass, waist–hip ratio, SAD), and health-related quality of life assessed via the SF-12 questionnaire. Safety outcomes included between group differences in the frequency and type of adverse events, serious or device-related adverse events and changes from baseline to Week 12 in vital signs (systolic and diastolic blood pressure, pulse rate), and laboratory safety parameters encompassing haematology, liver, and renal function. Further details are provided in the Clinical Investigation and Statistical Analysis Plan (Supplementary Material).

Post hoc analysis was conducted to evaluate within-group changes from baseline to Week 12 in HbA1c, body weight, LDL-C, total cholesterol, fat mass, fat-free mass, HOMA-B, SAD, and WC. To evaluate the overall metabolic effect of SiPore21, a composite analysis was also performed. For each participant in the SiPore21 and placebo groups, the percentage change from baseline to Week 12 was calculated for the following nine metabolic parameters: HbA1c, body weight, LDL-C, total cholesterol, fat mass, SAD, WC, fasting blood glucose, and HOMA-B. Since a decrease in HOMA-B indicates worsening β-cell function (in contrast to improvements in the other parameters), percentage changes for HOMA-B were inverted so that positive values consistently reflected metabolic improvement across all measures. The percentage change values for these nine parameters were then averaged to generate a single composite value for each group, referred to as the Composite Metabolic Risk, representing the overall metabolic response to treatment.

### Statistical analysis

It was estimated that a sample size of 288 participants (144 per group) would be the minimum required to achieve 80% power to detect a mean HbA1c difference of −0.7 mmol/mol between groups (with a standard deviation of 2 mmol/mol) from baseline to Week 12, assuming 10% attrition and a two-sided α = 0.05. This corresponds to a prespecified requirement of 130 participants per group completing the study, with additional participants randomised to account for anticipated dropout, consistent with the Statistical Analysis Plan. The primary analysis was performed on the Full Analysis Set (FAS), which included all participants who received at least one dose and at least one post-baseline HbA1c value. The Safety Analysis Set (SAS) included all participants who received at least one dose. Post hoc analyses were conducted using the Per-Protocol Set (PPS), which included the subset of participants in FAS who completed the study without any deviations. All efficacy outcomes reported in this article are based on post hoc analyses, and safety outcomes are presented from the planned safety analyses.

The primary endpoint was analysed using an ANCOVA model with baseline HbA1c as a covariate. The secondary endpoint was analysed using a similar model with baseline body weight as a covariate. Both models included treatment group, BMI category (<30 vs. ≥30 kg/m^2^), and screening HbA1c category (<48 vs. ≥48 mmol/mol [<6.5 vs. ≥6.5%]) as fixed factors. Distributional assumptions for the ANCOVA models were assessed according to the prespecified statistical analysis plan. Normality of model residuals was evaluated using the Kolmogorov–Smirnov test, and homogeneity of variances was assessed using Levene’s test within the GLM procedure. A p-value < 0.05 was considered to indicate violation of the respective assumption. If these assumptions were violated, a Wilcoxon rank-sum test was performed as a supportive analysis. Quade’s non-parametric ANCOVA was also performed, yielding results consistent with the primary analysis. Missing values for the primary and secondary endpoints were handled using multiple imputation (PROC MI, SAS) with a fully conditional specification (FCS) regression method. The majority of missing data were attributable to visits occurring outside the protocol-defined assessment window, resulting in exclusion per the statistical analysis plan; for the primary and main secondary endpoints, this accounted for >97% of missing values and was balanced across treatment groups. Safety data were analysed as collected. The imputation model included treatment group, longitudinal HbA1c measurements from baseline to Week 12, and randomisation stratification factors (baseline HbA1c category and BMI category). A total of 100 imputations were generated, and estimates were combined using Rubin’s rules (PROC MIANALYZE). The primary analysis for all endpoints was based on complete-case analyses, using ANCOVA (without imputation) and the Wilcoxon rank-sum test as appropriate, including only participants with non-missing data for the relevant endpoints and covariates. Multiple imputation was applied as a sensitivity analysis for the primary (HbA1c) and main secondary (body weight) endpoints, as specified in the statistical analysis plan, and yielded results consistent with the primary analysis. All main analyses were conducted using Statistical Analysis System version 9.4 or higher. Details can be found in the statistical analysis plan (Supplementary Material).

Post hoc exploratory endpoints included changes in HbA1c, body weight, LDL-C, total cholesterol, HOMA-B, fat mass, fat-free mass, SAD, and WC. Continuous variables were analysed using the Wilcoxon signed-rank test for within-group comparisons and the Mann–Whitney U test for between-group comparisons. Longitudinal changes were assessed using a mixed-effects model with repeated measures, applying Sidak’s correction for multiple comparisons. Categorical outcomes were analysed using McNemar’s test (glycaemic status) or the Chi-square test (distribution of sex). Correlations between continuous variables were evaluated using linear regression analysis. Statistical analyses were based on the change from baseline to Week 12, unless otherwise stated. All statistical tests were two-sided and p-values < 0.05 were considered significant. Unless otherwise specified, results are presented as mean change from baseline to Week 12 (±SEM). Exact p-values for all outcomes are provided in Table 2 and Supplementary Table S6. Post hoc analyses were performed using GraphPad Prism (version 10.4 or higher). For the post hoc analysis, outlier detection was conducted, blinded to group allocation. Outlier exclusion was based on assessment of physiological plausibility and identification of measurement artefacts (e.g., inconsistencies across related variables), rather than predefined statistical thresholds. For lipid parameters, values were excluded if identified as isolated distributional outliers after percentage transformation. For body composition, artefacts from bioimpedance measurements were removed following cross-validation with weight data. A total of five outliers (three in the SiPore21 group, two in the placebo group) were excluded from total cholesterol, four from LDL-C (two in the SiPore21 group, two in the placebo group), and 27 from body composition analyses (16 in the SiPore21 group, 11 in the placebo group).

### Role of the funding source

The sponsor provided the study intervention (SiPore21) and the placebo. The sponsor was involved in the design of the study, collection and analysis of data, interpretation of results, preparation of the manuscript, and the decision to submit the article for publication.

## Results

**Fig. 1:**
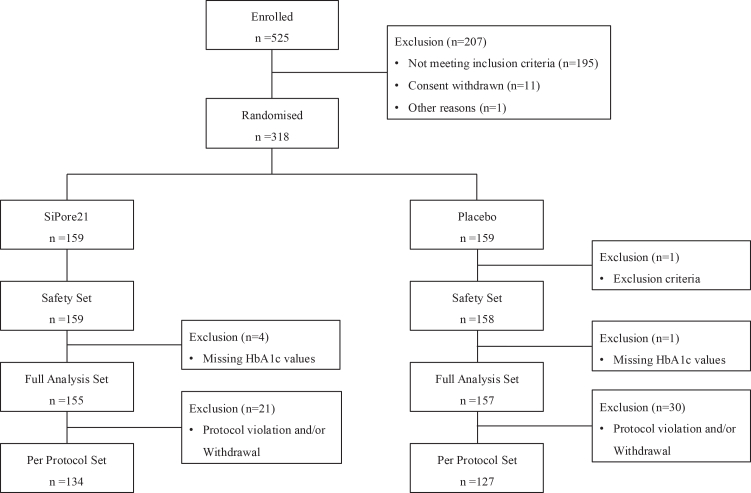
**Flow diagram of the study**.

The study population comprised individuals with prediabetes or early type 2 diabetes who met predefined inclusion and exclusion criteria. Of the 525 individuals screened, 207 were excluded mostly due to not meeting the eligibility criteria (195 of 207). A total of 318 participants were randomised equally to receive either SiPore21 or placebo. One participant in the placebo group was withdrawn before treatment initiation due to an exclusion criterion, leaving 317 participants who received at least one dose of study product. A total of 312 participants completed the 12-week intervention (Fig. 1). The baseline characteristics of participants are shown in Table 1. The distribution of sex was comparable between treatment groups (Table 1).

**Table 1:** Baseline characteristics of participants.

Characteristics	Safety analysis set		Per protocol set	
	SiPore21 (n = 159)	Placebo (n = 158)	SiPore21 (n = 134)	Placebo (n = 127)
Gender, (n [%])				
Female	96 (60.4)	76 (48.1)	81 (60.4)	62 (48.8)
Male	63 (39.6)	82 (51.9)	53 (39.6)	65 (51.2)
Age (years), mean ± SD	57.9 ± 9.3	57.3 ± 9.1	57.8 ± 9.7	57 ± 9.2
Race/Ethnicity, (n [%])				
Asian	0 (0.0)	0 (0.0)	0 (0.0)	0 (0.0)
Black or African American	0 (0.0)	0 (0.0)	0 (0.0)	0 (0.0)
White	159 (100)	157 (99.4)	134 (100)	126 (99.2)
Hispanic or Latino	0 (0.0)	0 (0.0)	0 (0.0)	0 (0.0)
Other	0 (0.0)	1 (0.6)	0 (0.0)	1 (0.8)
HbA1c (mmol/mol [%]), mean ± SD	46.8 (6.4) ± 4.3	46.5 (6.4) ± 4.1	46.9 (6.4) ± 4.1	46.4 (6.4) ± 4
Body weight (kg), mean ± SD	95.1 ± 16	99.4 ± 17	95.5 ± 15.8	100.5 ± 17.3
BMI (kg/m^2^), mean ± SD	33.6 ± 4.2	33.7 ± 4.2	33.7 ± 4.1	34 ± 4.3

### Efficacy outcomes

**Fig. 2:**
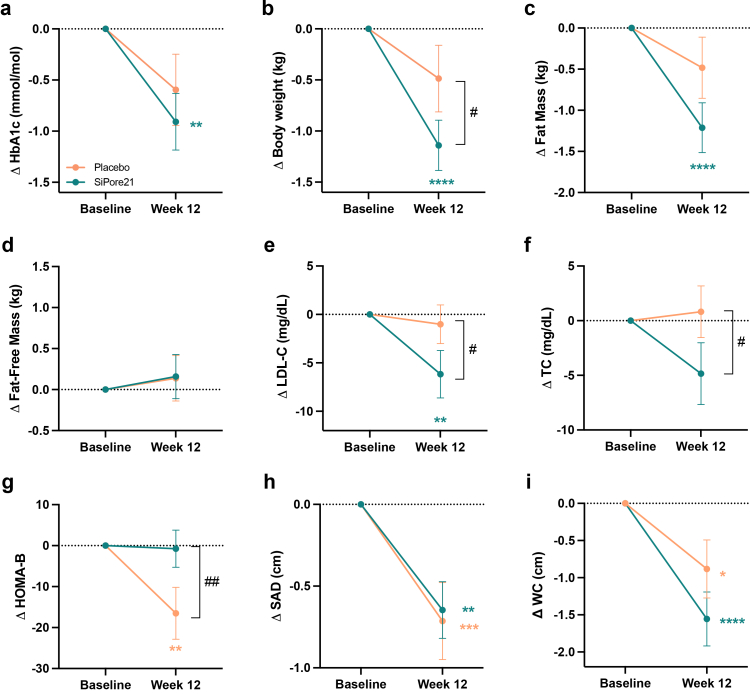
**Effects of 12-week SiPore21 treatment compared with placebo on primary and secondary outcomes.** Shown are changes from baseline to 12 weeks in participants receiving SiPore21 or placebo for (a) haemoglobin A1c (HbA1c), (b) body weight, (c) fat mass, (d) fat-free mass, (e) low-density lipoprotein cholesterol (LDL-C), (f) total cholesterol (TC), (g) homeostatic model assessment of β-cell function (HOMA-B), (h) sagittal abdominal diameter (SAD), and (i) waist circumference (WC). Data are presented as mean ± standard error of the mean (SEM). Sample sizes (SiPore21/placebo): n = 134/127 for HbA1c, body weight, WC; n = 134/125 for SAD; n = 124/119 for fat mass, fat-free mass; n = 124/114 for HOMA-B; n = 132/126 for LDL-C; n = 131/125 for TC. Statistical significance was assessed using a mixed-effects model with repeated measures and Sidak’s correction for multiple comparisons: ∗p < 0.05, ∗∗p < 0.01, ∗∗∗p < 0.001, ∗∗∗∗p < 0.0001 vs. baseline and ^#^p < 0.05, ^##^p < 0.001 vs. placebo; non-significant comparisons (p ≥ 0.05) are not annotated in the figure.

**Fig. 3:**
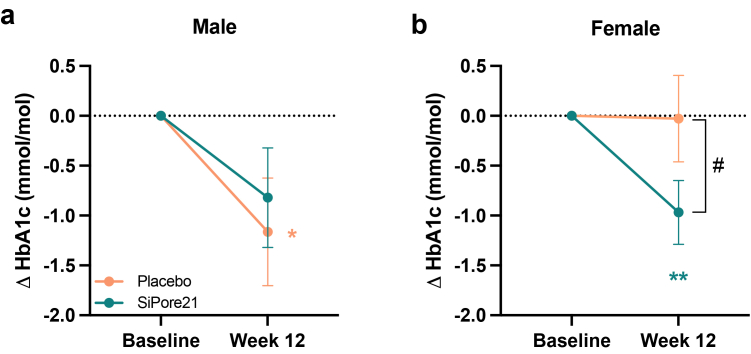
**Changes in HbA1c levels in males and females (post hoc subgroup analysis).** Shown are changes from baseline to 12 weeks in haemoglobin A1c (HbA1c) for (a) males and (b) females receiving SiPore21 or placebo. Data are presented as mean ± standard error of the mean (SEM). Sample sizes (SiPore21/placebo): n = 53/65 for males and n = 81/62 for females. Statistical significance was assessed using a mixed-effects model with repeated measures and Sidak’s correction for multiple comparisons: ∗p < 0.05, ∗∗p < 0.01 vs. baseline; ^#^p < 0.05 vs. placebo; non-significant comparisons (p ≥ 0.05) are not annotated in the figure.

**Fig. 4:**
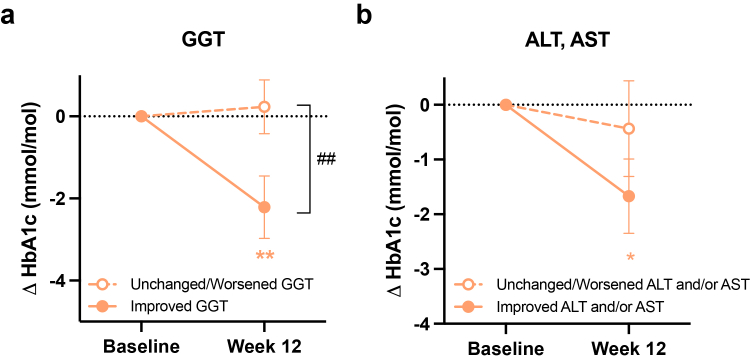
**Changes in HbA1c levels in placebo-treated males stratified by liver enzyme changes (post hoc subgroup analysis).** Shown are changes from baseline to 12 weeks in haemoglobin A1c (HbA1c) among placebo-treated male participants stratified by changes in liver enzyme levels: (a) gamma-glutamyl transferase (GGT) and (b) alanine aminotransferase (ALT) and/or aspartate aminotransferase (AST). Participants were categorised according to whether enzyme levels decreased (improved) or remained unchanged/increased (unchanged/worsened) from screening to Week 12. Sample sizes (improved/unchanged–worsened): n = 38/27 for GGT and n = 37/28 for ALT and/or AST. Data are presented as mean ± standard error of the mean (SEM). Statistical significance was assessed using a mixed-effects model with repeated measures and Sidak’s correction for multiple comparisons: ∗p < 0.05, ∗∗p < 0.01 vs. baseline; ^##^p < 0.01 vs. unchanged/worsened group; non-significant comparisons (p ≥ 0.05) are not annotated in the figure.

**Fig. 5:**
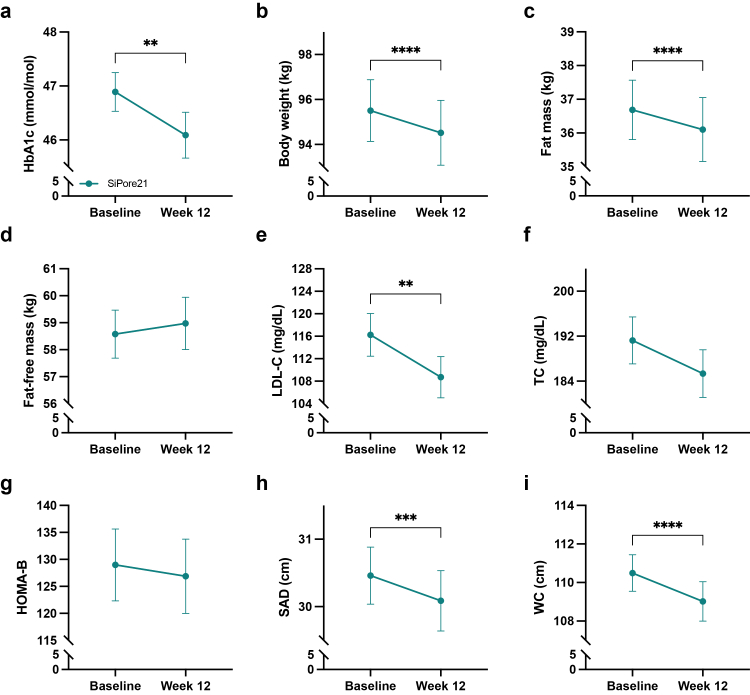
**Effects of 12-week SiPore21 treatment on metabolic parameters (post hoc within-group analysis).** Changes are shown for (a) haemoglobin A1c (HbA1c), (b) body weight, (c) fat mass, (d) fat-free mass, (e) low-density lipoprotein cholesterol (LDL-C), (f) total cholesterol (TC), (g) homeostatic model assessment of β-cell function (HOMA-B), (h) sagittal abdominal diameter (SAD), and (i) waist circumference (WC) compared to baseline. Data are presented as mean ± standard error of the mean (SEM); n = 134 for HbA1c, body weight, WC, SAD; n = 124 for fat mass, fat-free mass, HOMA-B; n = 132 for LDL-C; n = 131 for TC. Statistical significance was assessed using Wilcoxon signed-rank test; ∗p < 0.05, ∗∗p < 0.01, ∗∗∗p < 0.001, ∗∗∗∗p < 0.0001 vs. baseline; non-significant comparisons (p ≥ 0.05) are not annotated in the figure.

Twelve weeks of SiPore21 treatment resulted in significant improvements across multiple metabolic parameters (Figs. 2 and 5, Table 2, Supplementary Table S6). HbA1c levels were significantly reduced from baseline by an average of −0.91 mmol/mol (Figs. 2a and 5a). This reduction was also significantly correlated with improvements in body weight, fat mass, fasting glucose, insulin, and LDL-C levels (Supplementary Fig. S1). In line with these findings, HOMA-B levels remained stable in the SiPore21 group over the 12-week period (Figs. 2g and 5g), while a significant decline was noted in the placebo group (Fig. 2g). Although HbA1c levels improved significantly from baseline in the SiPore21 group, the between-group difference was not statistically significant (Fig. 2a). Post hoc analysis suggested that this may be due to a pronounced placebo response among male participants (Fig. 3a). Placebo-treated males showed a significant reduction in HbA1c (Fig. 3a), whereas no such effect was observed in females (Fig. 3b). Moreover, within the placebo group, males who showed improvements in liver enzyme levels, namely gamma-glutamyl transferase (GGT; Fig. 4a), alanine aminotransferase (ALT) and/or aspartate aminotransferase (AST; Fig. 4b) also exhibited corresponding reductions in HbA1c.

**Table 2:** Change from baseline to Week 12 in primary, secondary, and post hoc metabolic outcomes with between-group comparisons.

Primary/secondary outcomes (unit)	Change from baseline to week 12 (Mean ± SD)		Between-group p-value
	SiPore21	Placebo	
HbA1c (mmol/mol)	−0.91 ± 2.98	−0.60 ± 3.69	0.5134
Body weight (kg)	−1.14 ± 2.65	−0.49 ± 3.45	0.0374
Fat mass (kg)	−1.21 ± 3.11	−0.48 ± 3.79	0.0500
Fat-free mass (kg)	0.16 ± 2.77	0.14 ± 2.82	0.9959
LDL-C (mg/dL)	−6.17 ± 26.17	−1.02 ± 20.93	0.0350
TC (mg/dL)	−4.84 ± 30.02	0.82 ± 24.81	0.0496
HOMA-B	−0.75 ± 46.04	−16.52 ± 61.82	0.0050
SAD (cm)	−0.71 ± 2.49	−0.65 ± 1.87	0.9306
WC (cm)	−1.56 ± 3.91	−0.88 ± 4.14	0.1262
Post hoc outcomes			
Male HbA1c (mmol/mol)	−0.82 ± 3.42	−1.16 ± 4.04	0.7500
Female HbA1c (mmol/mol)	−0.97 ± 2.66	−0.03 ± 3.24	0.0190
Composite metabolic risk score	−1.99 ± 6.34	−0.03 ± 6.14	0.0075
Prediabetes progression to T2D (%)	6.06	10.29	a0.0233
T2D reversion to prediabetes (%)	23.81	11.76	b0.0044

LDL-C; Low density lipoprotein cholesterol, TC; Total cholesterol, HOMA-B; Homeostatic model assessment of β-cell function, SAD; Sagittal abdominal diameter, WC; Waist circumference, T2D; Type 2 diabetes.

a Indicates a statistically significant change within the placebo group.

b Indicates a statistically significant change within the SiPore21 group.

SiPore21 treatment led to a significant reduction in body weight (−1.1 kg from baseline; Figs. 2b and 5b), which was also significantly lower compared to placebo (−0.49 kg from baseline for placebo; Fig. 2b). This reduction in body weight, though moderate in magnitude, was primarily driven by decreases in fat mass (Figs. 2c and 5c), with no significant changes in fat-free mass (Figs. 2d and 5d). In prespecified subgroup analyses, participants with a BMI ≥ 30 kg/m^2^ receiving SiPore21 showed a greater reduction in body weight compared with placebo (−1.18 vs. −0.22 kg; p = 0.0313), whereas no significant difference was observed in the BMI <30 kg/m^2^ subgroup. Post hoc analysis further indicated that participants in the placebo group gained significantly greater weight compared to the SiPore21 group (2.58 vs. 1.44 kg; p = 0.0275). Additionally, markers of visceral adiposity, including SAD and WC, also showed significant reductions from baseline (Fig. 5h and i).

SiPore21 led to measurable and favourable changes in lipid profiles. LDL-C levels decreased significantly by 6.2 mg/dL from baseline and were also significantly lower compared to the placebo group (Figs. 2e and 5e). SiPore21 also reduced total cholesterol levels by 4.8 mg/dL from baseline, a change that was significantly different compared to the placebo group (Figs. 2f and 5f). The most pronounced effects in LDL-C and total cholesterol were observed in participants not receiving concomitant statin therapy. In this statin-free subgroup, LDL-C was decreased by 8.9 mg/dL and total cholesterol by 7.4 mg/dL from baseline, with both changes significantly different from baseline and from the placebo group (Supplementary Fig. 2a and b).

**Fig. 6:**
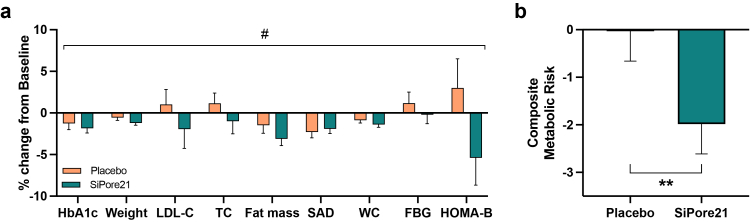
**Composite changes in metabolic risk factors following SiPore21 treatment (post hoc analysis).** (a) Shown are percentage changes from baseline to 12 weeks for each parameter with changes across all parameters evaluated collectively. Data are presented as mean ± standard error of the mean (SEM). Sample sizes (SiPore21/placebo): n = 116/112 for haemoglobin A1c (HbA1c) and body weight; n = 116/111 for waist circumference (WC); n = 116/110 for sagittal abdominal diameter (SAD); n = 110/106 for fat mass; n = 103/95 for the inverted value of homeostatic model assessment of β-cell function (HOMA-B); n = 114/110 for low-density lipoprotein cholesterol (LDL-C); n = 113/110 for total cholesterol (TC); n = 114/112 for fasting blood glucose (FBG). Statistical significance was assessed using a mixed-effects model with repeated measures and Sidak’s correction for multiple comparisons: ^#^p < 0.05 indicates a significant treatment effect across all parameters. (b) A Composite Metabolic Risk score was derived to reflect the overall metabolic impact of SiPore21 in each participant. Data are presented as mean ± SEM; n = 103/95 (SiPore21/placebo). Between-group comparison was conducted using the Mann–Whitney U test; ∗∗p < 0.01 vs. placebo.

To evaluate the overall metabolic impact of SiPore21, a composite analysis was conducted using percentage changes from baseline across multiple clinically relevant parameters (Fig. 6). This analysis confirmed a significant treatment effect across the set of parameters in the SiPore21 group (Fig. 6a). A Composite Metabolic Risk score was calculated for each participant to quantify the overall impact of SiPore21 on metabolic health. Participants receiving SiPore21 showed a statistically significant reduction in Composite Metabolic Risk compared to those receiving placebo (Fig. 6b), indicating a broad and simultaneous beneficial effect across multiple metabolic domains.

SiPore21 also demonstrated efficacy in stabilising glycaemic status and attenuating disease progression. Participants were stratified at baseline into prediabetes (HbA1c < 48 mmol/mol [<6.5%]) and T2D (HbA1c ≥ 48 mmol/mol [≥6.5%]) subgroups, and changes in glycaemic classification were assessed over time. Among participants with prediabetes, a statistically significant proportion progressed to T2D over 12 weeks in the placebo group, whereas no significant deterioration was observed in the SiPore21 group (Table 2). In participants with T2D at baseline, those receiving SiPore21 showed a statistically significant reversion to a prediabetic state, while no such improvement was observed in the placebo group (Table 2).

### Safety outcomes

SiPore21 was well tolerated throughout the 12-week intervention period, with high treatment adherence observed in both groups. Average compliance rates were 96.4% in the SiPore21 group and 97.1% in the placebo group, indicating consistent and reliable intake across participants. SiPore21 demonstrated an excellent safety profile, with adverse device effects (ADEs) reported in 13.2% of participants receiving SiPore21 and in 8.2% in the placebo group, showing no statistically significant difference between groups. No serious adverse device effects (SADEs) were reported in either treatment arm (Table 3).

**Table 3:** Summary of adverse events.

Adverse events	SiPore21 (n = 159)	Placebo (n = 158)
	n (%)	n (%)
Overall adverse events (AEs)	44 (27.7)	35 (22.2)
Adverse device effects (ADEs)	21 (13.2)	13 (8.2)
Serious AEs (SAEs)	2 (1.3)	4 (2.5)
Serious adverse device effects (SADEs)	0 (0.0)	0 (0.0)
AEs by relatedness		
Not related	28 (17.6)	29 (18.4)
Possible	12 (7.6)	7 (4.4)
Probable	8 (5.0)	2 (1.3)
Causal relationship	2 (1.3)	4 (2.5)
AEs that caused study withdrawal	5 (3.1)	5 (3.2)
AEs by severity		
Mild	28 (17.6)	27 (17.0)
Moderate	19 (12.0)	11 (7.0)
Severe	2 (1.3)	4 (2.5)
Deaths	0 (0.0)	1 (0.6)

The most common AEs were gastrointestinal in nature, reported in 14.5% of participants in the SiPore21 group and 8.2% in the placebo group. These included mild and transient symptoms such as diarrhoea, nausea, constipation, and flatulence (Table 4). Study withdrawals due to adverse events occurred equally in both groups, with five participants withdrawing in each arm, corresponding to approximately 3% of enrolled participants per group (Table 3).

**Table 4:** Adverse events by system organ class and preferred term.

Adverse events by		SiPore21 (n = 159)	Placebo (n = 158)
System organ class	Preferred term	n (%)	n (%)
Gastrointestinal disorders	Diarrhoea, nausea, constipation, flatulence etc.	23 (14.5)	13 (8.2)
Infections and infestations	COVID-19, nasopharyngitis, respiratory infection, etc.	11 (6.9)	14 (8.9)
Investigations	Vitamin D decreased, blood triglycerides increased, etc.	4 (2.5)	5 (3.2)
Musculoskeletal and connective tissue disorders	Arthralgia, back pain, spinal pain	2 (1.3)	4 (2.5)
Nervous system disorders	Headache, syncope, diabetic neuropathy, Guillain-Barre syndrome	3 (1.9)	2 (1.3)
Vascular disorders	Hypertension, aortic aneurysm rupture	2 (1.3)	3 (1.9)
Cardiac disorders	Angina pectoris, atrial fibrillation, tachycardia, myocardial infarction	2 (1.3)	2 (1.3)
Metabolism and nutrition disorders	Increased appetite, hyperuricaemia	3 (1.9)	1 (0.6)
Immune system disorders	Drug hypersensitivity, food allergy	1 (0.6)	1 (0.6)
Renal and urinary disorders	Nephrolithiasis, renal cyst	2 (1.3)	0 (0.0)
Endocrine disorders	Autoimmune thyroid disorder	1 (0.6)	0 (0.0)
General disorders and administration site conditions	Peripheral swelling	0 (0.0)	1 (0.6)
Hepatobiliary disorders	Liver disorder	1 (0.6)	0 (0.0)
Injury, poisoning and procedural complications	Hand fracture	0 (0.0)	1 (0.6)
Psychiatric disorders	Anxiety disorder	1 (0.6)	0 (0.0)
Reproductive system and breast disorders	Erectile dysfunction	1 (0.6)	0 (0.0)
Skin and subcutaneous tissue disorders	Dermal cyst	1 (0.6)	0 (0.0)

There were no clinically meaningful changes observed in haematological parameters, vital signs, or markers of liver and renal function over the course of the study. Furthermore, levels of vitamins and trace elements remained stable, with no clinically relevant deviations detected from baseline to Week 12 (Supplementary Tables S1–S5).

## Discussion

This randomised, double-blind, placebo-controlled multicentre trial demonstrated that 12 weeks of SiPore21 treatment resulted in clinically meaningful improvements across multiple metabolic domains in individuals with prediabetes or early T2D. Consistent with earlier clinical trials of SiPore21 prototypes, which reported efficacy in improving glycaemic outcomes,^2^^,^^5^^,^^6^ SiPore21 significantly reduced HbA1c by an average of 0.91 mmol/mol from baseline over the 12-week period. The magnitude of this effect is underscored by results from the Diabetes Prevention Program with individuals with prediabetes, where metformin achieved a reduction of approximately 0.6 mmol/mol only after six months of treatment.^14^ Despite the shorter intervention period in the present study, SiPore21 achieved a greater reduction, highlighting its potential as an effective, non-pharmacological option for early glycaemic management.

SiPore21 significantly reduced HbA1c from baseline, demonstrating a beneficial treatment effect. A greater reduction in HbA1c was also observed with SiPore21 compared with placebo; however, the between-group difference was not statistically significant, potentially reflecting a pronounced placebo response among male participants, as indicated by post hoc analyses. Evidence suggests that men are generally more susceptible to placebo responses than women,^15^^,^^16^ and biological differences, such as higher lean body mass and resting metabolic rate in men, may amplify physiological responses to subtle behavioural changes.^17^, ^18^, ^19^, ^20^, ^21^ In exploratory, post hoc analyses, a sex-specific response was observed in the present study: many male participants in the placebo group exhibited reductions in HbA1c, whereas no such effect was seen in females. Moreover, placebo-treated males who exhibited improvements in liver enzymes (GGT, ALT, AST), commonly interpreted as indicators of improved liver function,^22^^,^^23^ also showed corresponding reductions in HbA1c. Given the high sensitivity of GGT to alcohol intake, the observed pattern is consistent with inadvertent lifestyle modification among some male participants, most plausibly a reduction in alcohol consumption, despite explicit instructions to maintain habitual behaviours to prevent confounding of metabolic outcomes. This requirement was implemented for scientific control and not because such lifestyle improvements are undesirable. Such unreported behavioural changes, which might have been beneficial to health, appear to have influenced HbA1c outcomes in placebo-treated males, increasing variability and thereby diminishing the difference between SiPore21 and placebo in the overall study cohort.

SiPore21 significantly reduced body weight, driven primarily by fat mass reduction with preservation of fat-free (lean) mass. Even small reductions in weight have been shown to provide meaningful health benefits,^24^ with evidence suggesting that every kilogram of weight loss can improve metabolic markers and reduce the risks of diabetes progression and all-cause mortality.^25^^,^^26^ Maintenance of lean mass is critical for glucose metabolism and insulin sensitivity,^27^ and its preservation in this trial therefore strengthens the metabolic relevance of SiPore21-induced weight loss. Post hoc analyses further suggested that weight gain was greater in placebo group compared with the SiPore21 group; however, these findings should be interpreted as exploratory. Taken together, these findings suggest that SiPore21 may contribute to improvements in body composition and metabolic health in individuals with prediabetes and early T2D.

Cardiometabolic risk reduction was further supported by measurable and statistically significant reductions in LDL-C and total cholesterol, with more pronounced effects observed in participants not receiving statins, based on subgroup analyses. Elevated LDL-C is a key driver of atherosclerosis, and prediabetes itself is a recognised risk factor for cardiovascular disease. In this context, the lipid-lowering effects of SiPore21 may represent an additional clinical advantage, complementing existing lipid-lowering strategies.

Importantly, post hoc analyses revealed that SiPore21 demonstrated broad simultaneous metabolic benefits. These multifaceted effects of were captured by a reduction in Composite Metabolic Risk, reflecting SiPore21’s ability to concurrently target multiple interrelated risk factors. This approach is consistent with recent clinical studies that emphasize the importance of composite endpoints to evaluate metabolic interventions.^28^, ^29^, ^30^ Such integrative analyses provide a more clinically relevant assessment of long-term metabolic health, reinforcing the potential of SiPore21 as a comprehensive, non-pharmacological intervention.

Preventing progression from prediabetes to T2D is a primary focus in metabolic disease management. Beta-cell dysfunction is a key driver of progression, and by the time T2D is diagnosed, a substantial loss of β-cell has already occurred, emphasising the importance of early intervention.^31^ In the present study, SiPore21 preserved β-cell function, as reflected by stable HOMA-B levels compared with a significant decline in the placebo group. Furthermore, post hoc analyses indicated that SiPore21 attenuated glycaemic progression, with fewer participants with prediabetes advancing to T2D and a greater proportion of individuals with early T2D reverting to the prediabetic range. Collectively, these findings suggest that SiPore21 may help stabilise or even reverse early glycaemic deterioration. Although confirmation in long-term studies is needed, these results support the potential of SiPore21 to delay diabetes onset and preserve pancreatic function.

SiPore21 was safe and well tolerated throughout the 12-week intervention, with high adherence and no serious device-related adverse events reported. The incidence and nature of adverse events were comparable between groups and predominantly mild, transient gastrointestinal symptoms, such as diarrhoea, nausea, constipation, and flatulence, consistent with the device’s local intestinal mode of action. No clinically meaningful changes were observed in laboratory parameters, vital signs, or micronutrient levels. In addition to the observed safety data, the safety profile of SiPore21 is further supported by the established safety of its formulation components. The primary component of SiPore21, mesoporous silica, belongs to the class of synthetic amorphous silica, which is FDA-approved, generally recognised as safe (GRAS), and authorised as a food additive in Europe (E551). The silica used in SiPore21 complies with established purity requirements, and the additional formulation components consist of commonly used food-grade ingredients within their recognised safe use ranges. Taken together, the observed safety data, along with the established safety profile of the formulation components, support a favourable safety profile of SiPore21.

While these findings are encouraging, several limitations should be considered. First, the duration of the study was 12 weeks, which limits assessment of the long-term efficacy and safety, sustainability of metabolic benefits, and durability of glycaemic control, and potential long-term effects on nutrient absorption with SiPore21. Second, although the study was adequately powered for the primary endpoint, secondary and post hoc analyses were not individually powered and should therefore be interpreted as exploratory. Third, a pronounced placebo response, particularly among male participants, may have increased variability, attenuated the observable treatment effect, and contributed to the absence of a statistically significant difference in HbA1c between the SiPore21 and placebo groups. Finally, the magnitude of changes observed across individual endpoints was lower compared to established pharmacological therapies, and the clinical relevance of these effects should be interpreted with caution.

Overall, SiPore21 treatment led to clinically meaningful improvements across multiple metabolic domains, including glycaemic control, body composition, and lipid profiles, accompanied by a reduction in metabolic risk and a stabilisation of glycaemic status. Together with its favourable safety profile and strong adherence, SiPore21 represents a promising, multifaceted, non-pharmacological option to support individuals with prediabetes, those in the early stages of T2D, and metabolic health more broadly. Its localised gastrointestinal mode of action, without systemic absorption, further enhances its appeal for individuals seeking alternatives to pharmacological therapies. Future research should explore the long-term efficacy of SiPore21 and its applicability in broader populations, including those with established T2D.

## Contributors

TB, KHP, and SR contributed to the conceptualization and the study design.

TB, KHP, and SR contributed to the study methodology.

KHP was involved in the investigation and data collection.

JB, AI, GRN, MLB, EVJ, and TB were responsible for data analysis and interpretation.

JB, AI, GRN, and MLB worked on visualisation.

JB and AI contributed to the original draft.

All authors were involved in reviewing and editing the manuscript.

TB and GRN accessed and verified the data.

The authors take full responsibility for all aspects of the work, ensuring that any concerns regarding accuracy or integrity are properly addressed.

All authors had unrestricted access to all study data and held the final authority in the decision to submit the manuscript for publication.

## Data sharing statement

Anonymised data and clinical study report are available from the corresponding author upon request. Data will be available 3 months after article publication; availability will end 15 months following article publication. Requests for data should be directed to the corresponding author via email.

## Declaration of generative AI and AI-assisted technologies in the manuscript preparation process

During the preparation of this work the author(s) used ChatGPT (OpenAI, GPT-4.1) solely to improve language, clarity and readability of the manuscript. It was not used to generate scientific insights, analyse data, interpret results or draw conclusions. Assistance was applied only to author-written text after the scientific content had been fully drafted. After using this tool, the author(s) reviewed and edited the content as needed and take(s) full responsibility for the content of the published article.

## Declaration of interests

TB is supported by the Swedish Research Council (grant agreement numbers 2019-01508 and 2024-02446), the Eurostars project SYNSTAR (grant agreement number E114421) and Diabetes Wellness Sverige. TB is a board member of Sigrid Therapeutics AB and Atrogi AB, and a shareholder in Sigrid Therapeutics AB, Atrogi AB and Glucox Biotechnology AB. JB, AI, GRN, MLB, and EVJ are employees and shareholders of Sigrid Therapeutics AB, which funded and sponsored the study, including study design, data collection, analysis and manuscript preparation. JB, AI, GRN, MLB, EVJ, and TB are named inventors on patent applications owned by Sigrid Therapeutics related to mesoporous silica and SiPore® technology. KHP is supported by the Academy of Finland (grant numbers 266286, 272376, 314383, 335443, and 369181), the Finish Medical Foundation, the Gyllenberg Foundation, Novo Nordisk Foundation (grant numbers NNF20OC0060547, NNF17OC0027232, NNF10OC1013354, NNF25SA0103783, NNF24OC0091683), the Finnish Diabetes Research Foundation, the Finnish Foundation for Cardiovascular Research, the Paulo Foundation, the Sigrid Jusélius Foundation, the University of Helsinki and Helsinki University Hospital, and Government Research funds. SR has received consulting fees from Sigrid Therapeutics AB (paid to Rössner Resurs HB) and serves on a Scientific Advisory Board for Sigrid Therapeutics AB. The authors have no other relevant affiliations or financial involvement with any organization or entity with a financial interest in or financial conflict with the subject matter or materials discussed in the manuscript apart from those disclosed above.

## Appendix A Supplementary data

Supplementary Fig. S1 **Supplementary fig. 1. Association between changes in HbA1c and other metabolic parameters after 12 weeks of SiPore21 (post hoc analysis).** Relationships between changes in hemoglobin A1c (HbA1c) and changes in (a) fasting blood glucose (FBG), (b) fasting blood insulin (FBI), (c) low-density lipoprotein cholesterol (LDL-C), (d) body weight, and (e) fat mass, were evaluated by using simple linear regression. Data are shown as individual participant values with fitted regression lines. Goodness of fit is indicated by the as R^2^ value. Sample sizes: n = 116 for HbA1c and body weight; n = 114 for FBG and LDL-C; n = 112 for FBI; n = 106 for fat mass. Statistical significance: ∗*p* < 0.05, ∗∗*p* < 0.01, ∗∗∗∗*p* < 0.0001.

Supplementary Fig. S2 **Supplementary fig. 2. Impact of SiPore21 on lipid parameters in statin-naïve participants (post hoc analysis).** Shown are changes from baseline to 12 weeks in (a) low-density lipoprotein cholesterol (LDL-C) and (b) total cholesterol (TC) among participants not receiving concomitant statin therapy. Data are presented as mean ± standard error of the mean (SEM). Sample sizes (SiPore21/placebo): n = 75/74 for LDL-C, n = 75/75 for TC. Statistical comparisons were assessed using a mixed-effects model with repeated measures and Sidak’s correction for multiple comparisons: ∗*p* < 0.05, ∗∗*p* < 0.01 vs. baseline and ##*p* < 0.01 vs. placebo.

Supplementary Tables S1–S6

Study Protocol_SHINE

Statistical Analysis Plan_SHINE
