# Supplementary figures and images for: Effect of engineered mesoporous silica particles with tailored pore size on glycaemic control in individuals with prediabetes or type 2 diabetes: a randomised, double-blind, placebo-controlled SHINE trial
Source: eClinicalMedicine. 2026 Jul 2;97:104042. doi: 10.1016/j.eclinm.2026.104042 (PMC13352034; doi:10.1016/j.eclinm.2026.104042)

**a.**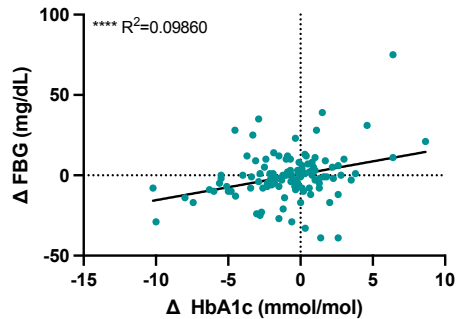**b.**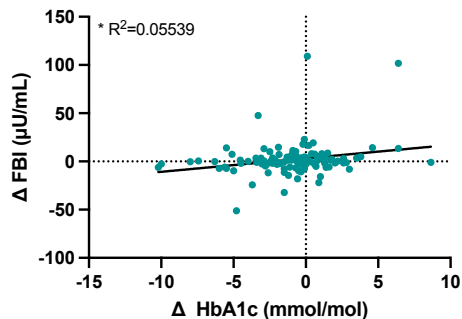**c.**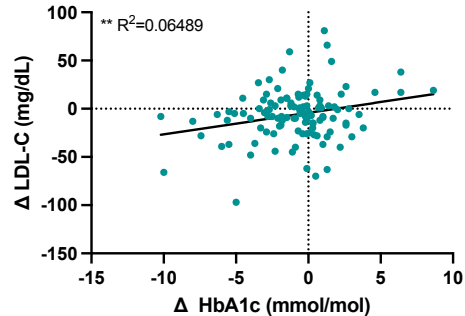**d.**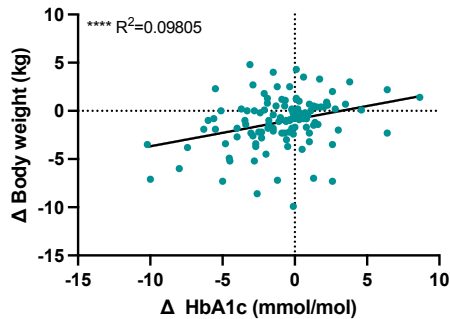**e.**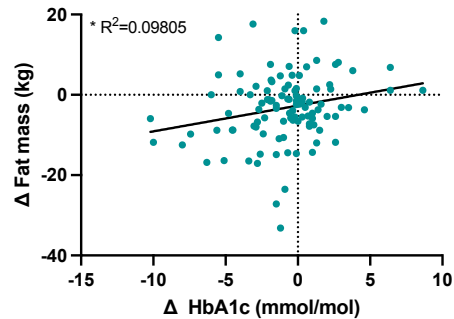

Supplement: Supplementary Fig. S1 — Supplementary fig. 1. Association between changes in HbA1c and other metabolic parameters after 12 weeks of SiPore21 (post hoc analysis). Relationships between changes in hemoglobin A1c (HbA1c) and changes in (a) fasting blood glucose (FBG), (b) fasting blood insulin (FBI), (c) low-density lipoprotein cholesterol (LDL-C), (d) body weight, and (e) fat mass, were evaluated by using simple linear regression. Data are shown as individual participant values with fitted regression lines. Goodness of fit is indicated by the as R2 value. Sample sizes: n = 116 for HbA1c and body weight; n = 114 for FBG and LDL-C; n = 112 for FBI; n = 106 for fat mass. Statistical significance: ∗p < 0.05, ∗∗p < 0.01, ∗∗∗∗p < 0.0001. [file mmc1.pdf]

a.

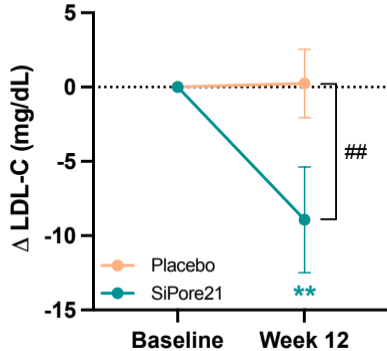

b.

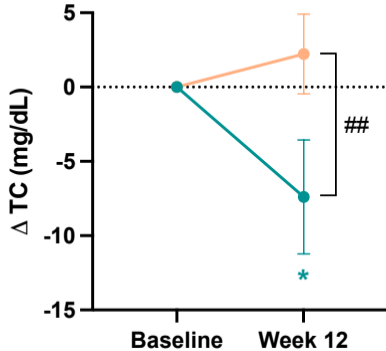

Supplement: Supplementary Fig. S2 — Supplementary fig. 2. Impact of SiPore21 on lipid parameters in statin-naïve participants (post hoc analysis). Shown are changes from baseline to 12 weeks in (a) low-density lipoprotein cholesterol (LDL-C) and (b) total cholesterol (TC) among participants not receiving concomitant statin therapy. Data are presented as mean ± standard error of the mean (SEM). Sample sizes (SiPore21/placebo): n = 75/74 for LDL-C, n = 75/75 for TC. Statistical comparisons were assessed using a mixed-effects model with repeated measures and Sidak’s correction for multiple comparisons: ∗p < 0.05, ∗∗p < 0.01 vs. baseline and ##p < 0.01 vs. placebo. [file mmc2.pdf]
