# Supplementary material for: Effect of engineered mesoporous silica particles with tailored pore size on glycaemic control in individuals with prediabetes or type 2 diabetes: a randomised, double-blind, placebo-controlled SHINE trial
Source: eClinicalMedicine. 2026 Jul 2;97:104042. doi: 10.1016/j.eclinm.2026.104042 (PMC13352034; doi:10.1016/j.eclinm.2026.104042)
Supplement: Supplementary Tables S1–S6 [file mmc3.docx]

**Supplementary Table 1.** Changes in haematological parameters

| **Haematological parameters** |  | | **SiPore21 N=159** | **Placebo N=158** |
| --- | --- | --- | --- | --- |
| Hemoglobin (g/dl) | Baseline | n | 159 | 158 |
|  | (Visit 2) | Mean ± SD | 14.50 ± 1.19 | 14.68 ± 1.49 |
|  |  | Median | 14.50 | 14.70 |
|  |  | Q1, Q3 | 13.70, 15.40 | 13.80, 15.60 |
|  |  | Min, Max | 11.80, 17.90 | 9.50, 18.50 |
|  | Week 12 | n | 149 | 147 |
|  | (Visit 4) | Mean ± SD | 14.47 ± 1.21 | 14.64 ± 1.47 |
|  |  | Median | 14.50 | 14.70 |
|  |  | Q1, Q3 | 13.70, 15.20 | 13.70, 15.70 |
|  |  | Min, Max | 11.10, 17.50 | 9.10, 17.80 |
|  | Change | n | 149 | 147 |
|  | (Week 12 – Baseline) | Mean ± SD | -0.06 ± 0.70 | -0.08 ± 0.75 |
|  |  | Median | -0.10 | 0.00 |
|  |  | Q1, Q3 | -0.50, 0.30 | -0.50, 0.40 |
|  |  | Min, Max | -1.70, 3.30 | -3.30, 2.20 |
| Hematocrit (%) | Baseline | n | 159 | 158 |
|  | (Visit 2) | Mean ± SD | 44.93 ± 3.57 | 45.50 ± 4.36 |
|  |  | Median | 44.60 | 45.10 |
|  |  | Q1, Q3 | 42.60, 47.00 | 42.90, 48.10 |
|  |  | Min, Max | 37.50, 58.10 | 33.90, 60.30 |
|  | Week 12 | n | 149 | 147 |
|  | (Visit 4) | Mean ± SD | 45.37 ± 3.97 | 45.80 ± 4.42 |
|  |  | Median | 45.20 | 45.70 |
|  |  | Q1, Q3 | 42.70, 48.10 | 42.80, 49.00 |
|  |  | Min, Max | 36.10, 57.60 | 32.00, 57.20 |
|  | Change | n | 149 | 147 |
|  | (Week 12 – Baseline) | Mean ± SD | 0.38 ± 3.21 | 0.23 ± 3.15 |
|  |  | Median | 0.20 | 0.30 |
|  |  | Q1, Q3 | -1.50, 2.20 | -1.90, 2.00 |
|  |  | Min, Max | -7.60, 13.50 | -9.00, 8.70 |
| Erythrocytes (T/l) | Baseline | n | 159 | 158 |
|  | (Visit 2) | Mean ± SD | 4.80 ± 0.40 | 4.85 ± 0.43 |
|  |  | Median | 4.83 | 4.81 |
|  |  | Q1, Q3 | 4.53, 5.08 | 4.57, 5.13 |
|  |  | Min, Max | 3.33, 5.86 | 3.74, 6.02 |
|  | Week 12 | n | 149 | 147 |
|  | (Visit 4) | Mean ± SD | 4.78 ± 0.41 | 4.82 ± 0.41 |
|  |  | Median | 4.77 | 4.83 |
|  |  | Q1, Q3 | 4.48, 5.05 | 4.54, 5.10 |
|  |  | Min, Max | 3.64, 5.95 | 3.68, 5.95 |
|  | Change | n | 149 | 147 |
|  | (Week 12 – Baseline) | Mean ± SD | -0.03 ± 0.24 | -0.04 ± 0.26 |
|  |  | Median | -0.06 | -0.03 |
|  |  | Q1, Q3 | -0.18, 0.08 | -0.16, 0.16 |
|  |  | Min, Max | -0.53, 1.10 | -0.98, 0.68 |
| Thrombocytes (G/l) | Baseline | n | 159 | 158 |
|  | (Visit 2) | Mean ± SD | 255.01 ± 65.36 | 258.61 ± 70.93 |
|  |  | Median | 247.00 | 248.00 |
|  |  | Q1, Q3 | 207.00, 291.00 | 209.00, 297.00 |
|  |  | Min, Max | 130.00, 492.00 | 92.00, 540.00 |
|  | Week 12 | n | 149 | 147 |
|  | (Visit 4) | Mean ± SD | 242.39 ± 60.35 | 254.07 ± 69.31 |
|  |  | Median | 237.00 | 248.00 |
|  |  | Q1, Q3 | 202.00, 276.00 | 207.00, 295.00 |
|  |  | Min, Max | 91.00, 410.00 | 62.00, 574.00 |
|  | Change | n | 149 | 147 |
|  | (Week 12 – Baseline) | Mean ± SD | -14.14 ± 37.30 | -4.27 ± 31.81 |
|  |  | Median | -10.00 | -2.00 |
|  |  | Q1, Q3 | -28.00, 7.00 | -19.00, 10.00 |
|  |  | Min, Max | -228.00, 79.00 | -143.00, 125.00 |
| Reticulocytes (G/l) | Baseline | n | 159 | 158 |
|  | (Visit 2) | Mean ± SD | 82.26 ± 20.33 | 85.94 ± 24.54 |
|  |  | Median | 80.00 | 81.50 |
|  |  | Q1, Q3 | 67.00, 96.00 | 68.00, 102.00 |
|  |  | Min, Max | 45.00, 170.00 | 38.00, 163.00 |
|  | Week 12 | n | 149 | 147 |
|  | (Visit 4) | Mean ± SD | 82.13 ± 22.98 | 83.20 ± 23.01 |
|  |  | Median | 81.00 | 81.00 |
|  |  | Q1, Q3 | 66.00, 96.00 | 68.00, 97.00 |
|  |  | Min, Max | 39.00, 166.00 | 21.00, 166.00 |
|  | Change | n | 149 | 147 |
|  | (Week 12 – Baseline) | Mean ± SD | -0.58 ± 15.51 | -2.61 ± 17.85 |
|  |  | Median | -1.00 | -1.00 |
|  |  | Q1, Q3 | -10.00, 8.00 | -10.00, 7.00 |
|  |  | Min, Max | -42.00, 70.00 | -69.00, 54.00 |
| Leucocytes (G/l) | Baseline | n | 159 | 158 |
|  | (Visit 2) | Mean ± SD | 7.23 ± 2.06 | 7.37 ± 2.00 |
|  |  | Median | 6.93 | 7.01 |
|  |  | Q1, Q3 | 5.81, 8.26 | 5.96, 8.57 |
|  |  | Min, Max | 2.90, 20.19 | 2.60, 15.18 |
|  | Week 12 | n | 149 | 147 |
|  | (Visit 4) | Mean ± SD | 7.15 ± 2.01 | 7.15 ± 2.07 |
|  |  | Median | 6.87 | 6.93 |
|  |  | Q1, Q3 | 5.79, 8.43 | 5.81, 7.92 |
|  |  | Min, Max | 3.80, 13.86 | 3.03, 14.66 |
|  | Change | n | 149 | 147 |
|  | (Week 12 – Baseline) | Mean ± SD | -0.09 ± 1.43 | -0.20 ± 1.47 |
|  |  | Median | -0.13 | -0.11 |
|  |  | Q1, Q3 | -0.78, 0.70 | -0.83, 0.54 |
|  |  | Min, Max | -6.33, 4.31 | -5.94, 3.89 |
|  | | | | |

**Supplementary Table 2.** Changes in liver function parameters

| **Liver function parameters** |  | | **SiPore21 N=159** | **Placebo N=158** |
| --- | --- | --- | --- | --- |
| ALAT (U/l) | Baseline | n | 159 | 158 |
|  | (Visit 2) | Mean ± SD | 34.81 ± 18.72 | 33.72 ± 18.07 |
|  |  | Median | 30.00 | 29.00 |
|  |  | Q1, Q3 | 24.00, 39.00 | 21.00, 41.00 |
|  |  | Min, Max | 14.00, 150.00 | 6.00, 126.00 |
|  | Week 12 | n | 153 | 150 |
|  | (Visit 4) | Mean ± SD | 37.72 ± 29.03 | 32.37 ± 15.40 |
|  |  | Median | 30.00 | 29.50 |
|  |  | Q1, Q3 | 24.00, 42.00 | 22.00, 38.00 |
|  |  | Min, Max | 12.00, 295.00 | 9.00, 99.00 |
|  | Change | n | 153 | 150 |
|  | (Week 12 – Baseline) | Mean ± SD | 2.47 ± 25.69 | -0.93 ± 10.67 |
|  |  | Median | 0.00 | 1.00 |
|  |  | Q1, Q3 | -6.00, 6.00 | -5.00, 4.00 |
|  |  | Min, Max | -46.00, 272.00 | -49.00, 40.00 |
| ASAT (U/l) | Baseline | n | 159 | 158 |
|  | (Visit 2) | Mean ± SD | 28.79 ± 10.77 | 28.38 ± 12.99 |
|  |  | Median | 27.00 | 26.00 |
|  |  | Q1, Q3 | 22.00, 32.00 | 21.00, 31.00 |
|  |  | Min, Max | 16.00, 81.00 | 7.00, 120.00 |
|  | Week 12 | n | 152 | 150 |
|  | (Visit 4) | Mean ± SD | 30.82 ± 14.26 | 27.84 ± 11.40 |
|  |  | Median | 27.00 | 25.00 |
|  |  | Q1, Q3 | 23.00, 34.00 | 21.00, 31.00 |
|  |  | Min, Max | 16.00, 113.00 | 15.00, 92.00 |
|  | Change | n | 152 | 150 |
|  | (Week 12 – Baseline) | Mean ± SD | 1.91 ± 11.83 | -0.19 ± 8.67 |
|  |  | Median | 0.00 | 0.00 |
|  |  | Q1, Q3 | -3.50, 4.00 | -3.00, 4.00 |
|  |  | Min, Max | -32.00, 96.00 | -43.00, 40.00 |
| Gamma-GT (U/l) | Baseline | n | 159 | 158 |
|  | (Visit 2) | Mean ± SD | 45.38 ± 74.90 | 42.13 ± 35.57 |
|  |  | Median | 30.00 | 29.00 |
|  |  | Q1, Q3 | 19.00, 47.00 | 20.00, 54.00 |
|  |  | Min, Max | 3.00, 879.00 | 3.00, 232.00 |
|  | Week 12 | n | 153 | 151 |
|  | (Visit 4) | Mean ± SD | 55.79 ± 116.94 | 38.97 ± 28.69 |
|  |  | Median | 27.00 | 29.00 |
|  |  | Q1, Q3 | 20.00, 49.00 | 20.00, 50.00 |
|  |  | Min, Max | 7.00, 1307.00 | 4.00, 179.00 |
|  | Change | n | 153 | 151 |
|  | (Week 12 – Baseline) | Mean ± SD | 9.64 ± 54.01 | -1.28 ± 14.13 |
|  |  | Median | 0.00 | 0.00 |
|  |  | Q1, Q3 | -4.00, 5.00 | -4.00, 4.00 |
|  |  | Min, Max | -87.00, 428.00 | -93.00, 33.00 |
| Alkaline Phosphatase (U/l) | Baseline | n | 159 | 158 |
|  | (Visit 2) | Mean ± SD | 79.19 ± 21.25 | 77.48 ± 21.92 |
|  |  | Median | 77.00 | 73.00 |
|  |  | Q1, Q3 | 62.00, 92.00 | 62.00, 91.00 |
|  |  | Min, Max | 40.00, 141.00 | 28.00, 161.00 |
|  | Week 12 | n | 153 | 151 |
|  | (Visit 4) | Mean ± SD | 79.47 ± 19.88 | 75.39 ± 19.69 |
|  |  | Median | 78.00 | 72.00 |
|  |  | Q1, Q3 | 65.00, 90.00 | 63.00, 86.00 |
|  |  | Min, Max | 38.00, 136.00 | 28.00, 139.00 |
|  | Change | n | 153 | 151 |
|  | (Week 12 – Baseline) | Mean ± SD | 0.07 ± 8.45 | -2.36 ± 14.78 |
|  |  | Median | 0.00 | -1.00 |
|  |  | Q1, Q3 | -5.00, 5.00 | -8.00, 6.00 |
|  |  | Min, Max | -26.00, 23.00 | -79.00, 24.00 |
| Bilirubin (mg/dl) | Baseline | n | 159 | 158 |
|  | (Visit 2) | Mean ± SD | 0.50 ± 0.31 | 0.50 ± 0.24 |
|  |  | Median | 0.42 | 0.45 |
|  |  | Q1, Q3 | 0.34, 0.55 | 0.32, 0.63 |
|  |  | Min, Max | 0.15, 2.08 | 0.16, 1.32 |
|  | Week 12 | n | 153 | 151 |
|  | (Visit 4) | Mean ± SD | 0.49 ± 0.29 | 0.50 ± 0.25 |
|  |  | Median | 0.41 | 0.46 |
|  |  | Q1, Q3 | 0.32, 0.55 | 0.35, 0.59 |
|  |  | Min, Max | 0.15, 1.91 | 0.15, 2.12 |
|  | Change | n | 153 | 151 |
|  | (Week 12 – Baseline) | Mean ± SD | -0.01 ± 0.19 | -0.01 ± 0.18 |
|  |  | Median | -0.01 | 0.00 |
|  |  | Q1, Q3 | -0.11, 0.09 | -0.10, 0.10 |
|  |  | Min, Max | -0.74, 0.63 | -0.54, 0.80 |
|  | | | | |

**Supplementary Table 3.** Changes in renal function parameters

| **Renal function parameters** |  | | **SiPore21 N=159** | **Placebo N=158** |
| --- | --- | --- | --- | --- |
| Creatinine (mg/dl) | Baseline | n | 159 | 158 |
|  | (Visit 2) | Mean ± SD | 0.88 ± 0.25 | 0.87 ± 0.19 |
|  |  | Median | 0.85 | 0.84 |
|  |  | Q1, Q3 | 0.75, 0.94 | 0.73, 1.00 |
|  |  | Min, Max | 0.47, 2.83 | 0.48, 1.50 |
|  | Week 12 | n | 153 | 151 |
|  | (Visit 4) | Mean ± SD | 0.84 ± 0.22 | 0.87 ± 0.19 |
|  |  | Median | 0.83 | 0.84 |
|  |  | Q1, Q3 | 0.71, 0.93 | 0.73, 1.00 |
|  |  | Min, Max | 0.45, 2.16 | 0.48, 1.58 |
|  | Change | n | 153 | 151 |
|  | (Week 12 – Baseline) | Mean ± SD | -0.03 ± 0.10 | 0.00 ± 0.10 |
|  |  | Median | -0.03 | -0.01 |
|  |  | Q1, Q3 | -0.07, 0.03 | -0.07, 0.04 |
|  |  | Min, Max | -0.67, 0.26 | -0.25, 0.52 |
| Urea (mg/dl) | Baseline | n | 159 | 158 |
|  | (Visit 2) | Mean ± SD | 33.48 ± 10.18 | 31.78 ± 8.00 |
|  |  | Median | 32.50 | 31.00 |
|  |  | Q1, Q3 | 27.60, 38.70 | 26.90, 36.60 |
|  |  | Min, Max | 9.80, 81.50 | 11.80, 61.80 |
|  | Week 12 | n | 153 | 151 |
|  | (Visit 4) | Mean ± SD | 32.40 ± 9.00 | 32.40 ± 8.71 |
|  |  | Median | 31.60 | 31.30 |
|  |  | Q1, Q3 | 25.70, 37.40 | 25.90, 37.50 |
|  |  | Min, Max | 14.60, 70.80 | 14.50, 57.90 |
|  | Change | n | 153 | 151 |
|  | (Week 12 – Baseline) | Mean ± SD | -1.07 ± 7.41 | 0.54 ± 8.17 |
|  |  | Median | -1.10 | 0.10 |
|  |  | Q1, Q3 | -5.20, 3.50 | -4.20, 5.10 |
|  |  | Min, Max | -36.30, 18.50 | -40.80, 23.70 |
| Cystatine C (mg/l) | Baseline | n | 159 | 158 |
|  | (Visit 2) | Mean ± SD | 1.01 ± 0.24 | 0.98 ± 0.20 |
|  |  | Median | 0.98 | 0.96 |
|  |  | Q1, Q3 | 0.86, 1.10 | 0.86, 1.08 |
|  |  | Min, Max | 0.58, 2.70 | 0.56, 1.71 |
|  | Week 12 | n | 153 | 151 |
|  | (Visit 4) | Mean ± SD | 1.00 ± 0.20 | 0.99 ± 0.22 |
|  |  | Median | 0.97 | 0.95 |
|  |  | Q1, Q3 | 0.85, 1.08 | 0.85, 1.09 |
|  |  | Min, Max | 0.58, 1.87 | 0.52, 1.74 |
|  | Change | n | 153 | 151 |
|  | (Week 12 – Baseline) | Mean ± SD | -0.01 ± 0.13 | 0.01 ± 0.12 |
|  |  | Median | 0.01 | 0.01 |
|  |  | Q1, Q3 | -0.06, 0.05 | -0.05, 0.06 |
|  |  | Min, Max | -0.83, 0.30 | -0.27, 0.59 |
| Uric acid (mg/dl) | Baseline | n | 159 | 158 |
|  | (Visit 2) | Mean ± SD | 5.93 ± 1.36 | 5.86 ± 1.30 |
|  |  | Median | 5.80 | 5.70 |
|  |  | Q1, Q3 | 4.90, 6.90 | 5.00, 6.50 |
|  |  | Min, Max | 3.20, 10.50 | 2.70, 12.40 |
|  | Week 12 | n | 153 | 151 |
|  | (Visit 4) | Mean ± SD | 5.85 ± 1.28 | 5.91 ± 1.32 |
|  |  | Median | 5.80 | 5.80 |
|  |  | Q1, Q3 | 4.90, 6.80 | 5.10, 6.70 |
|  |  | Min, Max | 3.30, 9.20 | 3.00, 10.20 |
|  | Change | n | 153 | 151 |
|  | (Week 12 – Baseline) | Mean ± SD | -0.05 ± 0.79 | 0.03 ± 0.83 |
|  |  | Median | 0.00 | 0.00 |
|  |  | Q1, Q3 | -0.50, 0.40 | -0.50, 0.60 |
|  |  | Min, Max | -3.10, 2.60 | -3.00, 2.70 |
| eGRF (mL/min/1.73m²) | Baseline | n | 159 | 158 |
|  | (Visit 2) | Mean ± SD | 85.79 ± 19.05 | 88.25 ± 16.80 |
|  |  | Median | 85.00 | 88.00 |
|  |  | Q1, Q3 | 72.00, 99.00 | 76.00, 99.00 |
|  |  | Min, Max | 22.00, 130.00 | 43.00, 140.00 |
|  | Week 12 | n | 153 | 151 |
|  | (Visit 4) | Mean ± SD | 87.14 ± 18.12 | 87.93 ± 18.57 |
|  |  | Median | 87.00 | 90.00 |
|  |  | Q1, Q3 | 75.00, 100.00 | 75.00, 102.00 |
|  |  | Min, Max | 33.00, 130.00 | 40.00, 144.00 |
|  | Change | n | 153 | 151 |
|  | (Week 12 – Baseline) | Mean ± SD | 0.97 ± 8.47 | -0.13 ± 8.39 |
|  |  | Median | 1.00 | 0.00 |
|  |  | Q1, Q3 | -3.00, 5.00 | -5.00, 5.00 |
|  |  | Min, Max | -24.00, 31.00 | -29.00, 23.00 |
|  | | | | |

**Supplementary Table 4.** Changes in vital Signs

| **Vital Signs** |  | | **SiPore21 N=159** | **Placebo N=158** |
| --- | --- | --- | --- | --- |
| Systolic Blood Pressure (mmHg) | Baseline | n | 159 | 158 |
|  | (Visit 2) | Mean ± SD | 132.04 ± 13.59 | 134.37 ± 12.78 |
|  |  | Median | 132.00 | 135.00 |
|  |  | Q1, Q3 | 122.00, 140.00 | 125.00, 144.00 |
|  |  | Min, Max | 96.00, 170.00 | 102.00, 180.00 |
|  | Week 6 | n | 150 | 152 |
|  | (Visit 3) | Mean ± SD | 131.41 ± 14.17 | 132.81 ± 12.57 |
|  |  | Median | 131.50 | 133.50 |
|  |  | Q1, Q3 | 123.00, 140.00 | 127.00, 140.00 |
|  |  | Min, Max | 95.00, 174.00 | 100.00, 165.00 |
|  | Week 12 | n | 155 | 152 |
|  | (Visit 4) | Mean ± SD | 131.80 ± 13.70 | 132.80 ± 13.41 |
|  |  | Median | 131.00 | 132.50 |
|  |  | Q1, Q3 | 122.00, 140.00 | 125.00, 140.00 |
|  |  | Min, Max | 98.00, 178.00 | 100.00, 170.00 |
|  | Change | n | 150 | 152 |
|  | (Week 6 – Baseline) | Mean ± SD | -0.60 ± 12.37 | -1.59 ± 13.23 |
|  |  | Median | 0.00 | 0.00 |
|  |  | Q1, Q3 | -8.00, 7.00 | -10.00, 6.50 |
|  |  | Min, Max | -41.00, 34.00 | -40.00, 31.00 |
|  | Change | n | 155 | 152 |
|  | (Week 12 – Baseline) | Mean ± SD | -0.23 ± 12.23 | -1.41 ± 14.74 |
|  |  | Median | 0.00 | 0.00 |
|  |  | Q1, Q3 | -8.00, 8.00 | -10.00, 9.00 |
|  |  | Min, Max | -34.00, 27.00 | -40.00, 37.00 |
| Diastolic Blood Pressure (mmHg) | Baseline | n | 159 | 158 |
|  | (Visit 2) | Mean ± SD | 80.92 ± 8.83 | 82.34 ± 8.87 |
|  |  | Median | 80.00 | 80.00 |
|  |  | Q1, Q3 | 76.00, 86.00 | 78.00, 89.00 |
|  |  | Min, Max | 53.00, 107.00 | 61.00, 107.00 |
|  | Week 6 | n | 150 | 152 |
|  | (Visit 3) | Mean ± SD | 80.36 ± 9.57 | 82.18 ± 8.89 |
|  |  | Median | 80.00 | 81.50 |
|  |  | Q1, Q3 | 73.00, 86.00 | 76.00, 89.00 |
|  |  | Min, Max | 57.00, 104.00 | 63.00, 105.00 |
|  | Week 12 | n | 155 | 152 |
|  | (Visit 4) | Mean ± SD | 80.94 ± 9.02 | 82.03 ± 8.66 |
|  |  | Median | 80.00 | 82.00 |
|  |  | Q1, Q3 | 76.00, 87.00 | 76.00, 88.00 |
|  |  | Min, Max | 48.00, 116.00 | 60.00, 100.00 |
|  | Change | n | 150 | 152 |
|  | (Week 6 – Baseline) | Mean ± SD | -0.35 ± 8.68 | -0.13 ± 9.72 |
|  |  | Median | 0.00 | 0.00 |
|  |  | Q1, Q3 | -5.00, 4.00 | -5.00, 5.00 |
|  |  | Min, Max | -30.00, 30.00 | -29.00, 26.00 |
|  | Change | n | 155 | 152 |
|  | (Week 12 – Baseline) | Mean ± SD | 0.10 ± 7.46 | -0.17 ± 9.49 |
|  |  | Median | 0.00 | 0.00 |
|  |  | Q1, Q3 | -4.00, 4.00 | -5.00, 5.00 |
|  |  | Min, Max | -18.00, 32.00 | -37.00, 27.00 |
| Pulse Rate (beats/min) | Baseline | n | 159 | 158 |
|  | (Visit 2) | Mean ± SD | 73.09 ± 9.71 | 73.30 ± 8.47 |
|  |  | Median | 72.00 | 73.00 |
|  |  | Q1, Q3 | 66.00, 80.00 | 68.00, 79.00 |
|  |  | Min, Max | 52.00, 103.00 | 52.00, 96.00 |
|  | Week 6 | n | 150 | 152 |
|  | (Visit 3) | Mean ± SD | 72.33 ± 9.99 | 73.97 ± 9.24 |
|  |  | Median | 71.50 | 75.00 |
|  |  | Q1, Q3 | 66.00, 79.00 | 68.00, 79.00 |
|  |  | Min, Max | 52.00, 98.00 | 52.00, 102.00 |
|  | Week 12 | n | 155 | 152 |
|  | (Visit 4) | Mean ± SD | 74.32 ± 10.55 | 74.44 ± 8.89 |
|  |  | Median | 74.00 | 74.50 |
|  |  | Q1, Q3 | 67.00, 81.00 | 68.00, 80.50 |
|  |  | Min, Max | 50.00, 105.00 | 53.00, 97.00 |
|  | Change | n | 150 | 152 |
|  | (Week 6 – Baseline) | Mean ± SD | -0.65 ± 7.51 | 0.57 ± 9.04 |
|  |  | Median | 0.00 | 0.00 |
|  |  | Q1, Q3 | -5.00, 4.00 | -5.50, 6.50 |
|  |  | Min, Max | -30.00, 23.00 | -31.00, 27.00 |
|  | Change | n | 155 | 152 |
|  | (Week 12 – Baseline) | Mean ± SD | 1.29 ± 8.84 | 1.16 ± 10.25 |
|  |  | Median | 2.00 | 0.00 |
|  |  | Q1, Q3 | -5.00, 6.00 | -6.00, 8.00 |
|  |  | Min, Max | -30.00, 33.00 | -23.00, 43.00 |
|  | | | | |

**Supplementary Table 5.** Changes in vitamins and trace elements

| **Vitamins and trace elements** |  | | **SiPore21 N=159** | **Placebo N=158** |
| --- | --- | --- | --- | --- |
| Vitamin B12 (pg/ml) | Baseline | n | 158 | 155 |
|  | (Visit 2) | Mean ± SD | 421.65 ± 165.01 | 405.49 ± 148.83 |
|  |  | Median | 394.00 | 376.00 |
|  |  | Q1, Q3 | 311.00, 492.00 | 305.00, 475.00 |
|  |  | Min, Max | 150.00, 1424.00 | 100.00, 1094.00 |
|  | Week 12 | n | 151 | 145 |
|  | (Visit 4) | Mean ± SD | 416.11 ± 148.30 | 403.97 ± 135.62 |
|  |  | Median | 386.00 | 387.00 |
|  |  | Q1, Q3 | 317.00, 483.00 | 298.00, 498.00 |
|  |  | Min, Max | 172.00, 991.00 | 196.00, 902.00 |
|  | Change | n | 150 | 143 |
|  | (Week 12 – Baseline) | Mean ± SD | -9.11 ± 96.62 | -2.69 ± 98.18 |
|  |  | Median | -10.50 | 0.00 |
|  |  | Q1, Q3 | -46.00, 28.00 | -45.00, 38.00 |
|  |  | Min, Max | -640.00, 351.00 | -520.00, 319.00 |
| Vitamin D (ng/ml) | Baseline | n | 159 | 157 |
|  | (Visit 2) | Mean ± SD | 27.64 ± 10.91 | 25.98 ± 11.74 |
|  |  | Median | 25.50 | 24.50 |
|  |  | Q1, Q3 | 19.80, 34.20 | 18.20, 32.20 |
|  |  | Min, Max | 5.20, 57.10 | 7.60, 98.70 |
|  | Week 12 | n | 153 | 151 |
|  | (Visit 4) | Mean ± SD | 29.19 ± 10.72 | 29.70 ± 10.93 |
|  |  | Median | 27.10 | 28.90 |
|  |  | Q1, Q3 | 22.80, 34.20 | 21.70, 37.00 |
|  |  | Min, Max | 8.80, 65.60 | 9.00, 69.20 |
|  | Change | n | 153 | 151 |
|  | (Week 12 – Baseline) | Mean ± SD | 1.51 ± 7.36 | 3.43 ± 8.39 |
|  |  | Median | 1.50 | 3.10 |
|  |  | Q1, Q3 | -3.20, 5.60 | -2.40, 8.20 |
|  |  | Min, Max | -22.00, 21.90 | -29.50, 27.30 |
| Mg (mg/dl) | Baseline | n | 158 | 157 |
|  | (Visit 2) | Mean ± SD | 2.00 ± 0.19 | 2.03 ± 0.18 |
|  |  | Median | 2.00 | 2.00 |
|  |  | Q1, Q3 | 1.90, 2.10 | 1.90, 2.20 |
|  |  | Min, Max | 1.20, 2.40 | 1.50, 2.50 |
|  | Week 12 | n | 153 | 151 |
|  | (Visit 4) | Mean ± SD | 2.00 ± 0.17 | 2.01 ± 0.18 |
|  |  | Median | 2.00 | 2.00 |
|  |  | Q1, Q3 | 1.90, 2.10 | 1.90, 2.10 |
|  |  | Min, Max | 1.30, 2.40 | 1.40, 2.50 |
|  | Change | n | 152 | 151 |
|  | (Week 12 – Baseline) | Mean ± SD | 0.00 ± 0.14 | -0.02 ± 0.15 |
|  |  | Median | 0.00 | 0.00 |
|  |  | Q1, Q3 | -0.10, 0.10 | -0.10, 0.10 |
|  |  | Min, Max | -0.40, 0.50 | -0.50, 0.50 |
| Zn (ug/dl) | Baseline | n | 152 | 148 |
|  | (Visit 2) | Mean ± SD | 107.02 ± 17.43 | 108.43 ± 18.86 |
|  |  | Median | 105.60 | 106.45 |
|  |  | Q1, Q3 | 95.10, 117.75 | 95.15, 118.55 |
|  |  | Min, Max | 67.60, 158.80 | 71.60, 181.20 |
|  | Week 12 | n | 141 | 142 |
|  | (Visit 4) | Mean ± SD | 106.84 ± 16.70 | 108.68 ± 17.89 |
|  |  | Median | 105.50 | 107.50 |
|  |  | Q1, Q3 | 94.70, 114.60 | 98.10, 116.70 |
|  |  | Min, Max | 64.60, 162.10 | 64.70, 169.80 |
|  | Change | n | 138 | 136 |
|  | (Week 12 – Baseline) | Mean ± SD | -0.19 ± 18.53 | 0.13 ± 23.38 |
|  |  | Median | 2.50 | 1.10 |
|  |  | Q1, Q3 | -11.10, 12.10 | -11.80, 13.80 |
|  |  | Min, Max | -64.00, 39.60 | -74.90, 62.30 |
|  | | | | |

**Supplementary Table 6:** Change from baseline to Week 12 in metabolic outcomes with within-group comparisons (post hoc analyses)

| **Primary/Secondary Outcomes**  **(unit)** | **SiPore21** | | **Placebo** | |
| --- | --- | --- | --- | --- |
|  | **Change from baseline to Week 12**  **(Mean ± SD)** | **Within-Group**  **p-value** | **Change from baseline to Week 12**  **(Mean ± SD)** | **Within-Group**  **p-value** |
| HbA1c (mmol/mol) | -0.91 ± 2.98 | 0.0036 | -0.60 ± 3.69 | 0.0872 |
| Body weight (kg) | -1.14 ± 2.65 | <0.0001 | -0.49 ± 3.45 | 0.1422 |
| Fat mass (kg) | -1.21 ± 3.11 | 0.0002 | -0.48 ± 3.79 | 0.2390 |
| Fat-free mass (kg) | 0.16 ± 2.77 | 0.7777 | 0.14 ± 2.82 | 0.8319 |
| LDL-C (mg/dL) | -6.17 ± 26.17 | 0.0060 | -1.02 ± 20.93 | 0.8630 |
| TC (mg/dL) | -4.84 ± 30.02 | 0.0890 | 0.82 ± 24.81 | 0.9321 |
| HOMA-B | -0.75 ± 46.04 | 0.9851 | -16.52 ± 61.82 | 0.0024 |
| SAD (cm) | -0.71 ± 2.49 | 0.0014 | -0.65 ± 1.87 | 0.0006 |
| WC (cm) | -1.56 ± 3.91 | <0.0001 | -0.88 ± 4.14 | 0.0269 |
| **Post hoc Outcomes** |  |  |  |  |
| Male HbA1c (mmol/mol) | -0.82 ± 3.42 | 0.2129 | -1.16 ± 4.04 | 0.0274 |
| Female HbA1c (mmol/mol) | -0.97 ± 2.66 | 0.0068 | -0.03 ± 3.24 | 0.9963 |

*Abbreviations:* LDL-C; Low density lipoprotein cholesterol, TC; Total cholesterol, HOMA-B; Homeostatic model assessment of β-cell function, SAD; Sagittal abdominal diameter, WC; Waist circumference.
