# Supplementary material for: Effect of engineered mesoporous silica particles with tailored pore size on glycaemic control in individuals with prediabetes or type 2 diabetes: a randomised, double-blind, placebo-controlled SHINE trial
Source: eClinicalMedicine. 2026 Jul 2;97:104042. doi: 10.1016/j.eclinm.2026.104042 (PMC13352034; doi:10.1016/j.eclinm.2026.104042)
Supplement: Study Protocol_SHINE [file mmc4.pdf]

DocuSign Envelope ID: DC97F66E-B279-4A14-AC63-5C291FAB6C7D

Clinical Investigation Plan

SITH/001921

Randomized, double-blinded, placebo-controlled, multicenter trial investigating performance and safety of the medical device SiPore21® in obese or overweight subjects with elevated blood glucose levels

Investigational Medical Device SiPore21®

Legal Manufacturer Sigrid Therapeutics AB

**Device classification**	Class IIb medical device according to the classification rules of the European Medical Device Regulation (EU) 2017/745 (rules 5 and 21)
**Sponsor signatory**	Name: Sana Alajmovic<br>Address: Sigrid Therapeutics AB<br>C/O 7A Odenplan<br>Norrtullsgatan 6, plan 5<br>SE-113 29 Stockholm, Sweden<br>Phone: +46 723 893 396<br>e-mail: sana@sigridthx.com
**Coordinating Investigator**	Name: Kirsi Pietiläinen, MD, Professor<br>Address: University of Helsinki<br>Haartmaninkatu 8<br>FI-00014 Helsinki, Finland<br>Phone: +358 50 5992295<br>E-mail: kirsi.pietilainen@helsinki.fi
**Reference number of the clinical investigation (CIV-ID)**	TBD

CONFIDENTIALITY STATEMENT

This document contains confidential information, which should not be copied, referred to, released or published without written approval from the Sponsor. Investigators are cautioned that the information given in this Clinical Investigation Plan (CIP) might be subject to change and revision. Any conclusion regarding efficacy and safety must be considered provisional.

The following amendments have been made to the Final CIP Version 1.0; 2023-04-05:

Amendment No.	Date of Amendment	Revised CIP Version
1	*24 Jul 2023*	*2.0*

DocuSign Envelope ID: DC97F66E-B279-4A14-AC63-5C291FAB6C7D

TABLE OF CONTENTS

2 SYNOPSIS ... 8

3 LIST OF ABBREVIATIONS AND DEFINITION OF TERMS ... 18

4 ETHICAL AND REGULATORY REQUIREMENTS ... 21

4.1 Regulatory Aspects ... 21

4.2 Independent Ethics Committee/Regulatory Authority ... 21

4.3 Subject Information and Informed Consent Form ... 21

4.4 Investigator's Obligations ... 22

4.5 Data Protection and Confidentiality ... 22

5 RISKS AND BENEFITS OF THE INVESTIGATIONAL MEDICAL DEVICE AND CLINICAL INVESTIGATION ... 24

5.1 Anticipated Clinical Benefit of the IMD ... 24

5.2 Anticipated Adverse Device Effects ... 24

5.2.1 Risk Associated With the IMD ... 24

5.2.2 Risks due to Manufacturing Errors ... 25

5.2.3 Risks due to Improper Use Including Overdose ... 25

5.2.4 Risks due to Interactions with Pharmaceutical Products ... 25

5.2.5 Overall Risk Assessment ... 25

5.3 Risks Associated with the Participation in the Clinical Investigation ... 25

5.4 Rationale for Benefit-Risk Ratio ... 27

6 INVESTIGATOR(S) AND INVESTIGATION ADMINISTRATIVE STRUCTURE ... 28

7 INTRODUCTION ... 30

7.1 Background ... 30

7.2 Investigational Medical Device ... 31

7.3 Non-Clinical Summary ... 32

7.3.1 Biocompatibility and Toxicity ... 32

7.3.1.1 Physical and chemical characterization ... 32

7.3.1.2 Cytotoxicity ... 32

7.3.1.3 Genotoxicity ... 32

7.3.1.4 Irritation and Sensitization ... 32

7.3.2 Other Non-Clinical Data ... 32

7.4 Clinical Experience 33

7.5 Rationale for the Investigation 34

8 INVESTIGATIONAL OBJECTIVES AND ENDPOINTS 35

8.1 Hypothesis 35

8.2 Primary and Secondary Objectives and Endpoints 35

8.2.1 Justification of Endpoints 36

8.3 Safety/Tolerability Endpoints 37

8.4 Other Explorative Parameters 39

9 INVESTIGATIONAL PLAN 40

9.1 Overall Investigational Design and Schedule of Events 40

9.2 Duration of the Study 45

9.3 Justification for the Design of the Clinical Investigation 45

9.4 Selection of Investigational Population 46

9.4.1 Number of Subjects 46

9.4.2 Screening and Enrolment Log 46

9.4.3 Inclusion Criteria 46

9.4.4 Exclusion Criteria 47

9.4.5 Women of Childbearing Potential 48

9.4.6 Restrictions 49

9.5 Identification and Description of the Investigational Medical Device and Placebo 49

9.5.1 Description of the IMD and Placebo 49

9.5.2 Manufacturer of the IMD and Placebo 51

9.5.3 Production, Packaging and Labelling of IMD and Placebo 53

9.5.4 Storage, Inventory, Return, Traceability and Documentation of the IMD and Placebo 53

9.5.5 Intended Purpose of the IMD in the Proposed Clinical Investigation 53

9.5.6 Populations and Indications for which the IMD is Intended 54

9.5.7 Required Training and Experience of Users 54

9.5.8 Medical or Surgical Procedures Involved in the Use of the Device 54

9.5.9 Method of Assigning Subjects to Treatment Groups and Blinding 54

9.5.10 Blinding and Emergency Decoding of Blinded Treatment 55

DocuSign Envelope ID: DC97F66E-B279-4A14-AC63-5C291FAB6C7D

9.6 Investigational Assessments 55
9.6.1 Visit Schedule 55
9.6.1.1 Visit 1 (V1, screening), Day -14 to Day -5 55
9.6.1.2 Visit 2 (baseline/randomization), Day 0 56
9.6.1.3 Phone Call 1, Day 21 ±3 (3 weeks ±3 days after V2) 57
9.6.1.4 Visit 3 (control), Day 42 ±3 (6 weeks ±3 days after V2) 57
9.6.1.5 Phone Call 2, Day 63 ±5 (9 weeks ±5 days after V2) 57
9.6.1.6 Visit 4 (final visit), Day 84 ±5 (12 weeks ±5 days after V2) 58
9.6.1.7 Phone Call 3, 7-10 days after V4 58
9.6.1.8 Phone Call 4, 21-35 days after V4 59
9.6.2 Screening Procedures 59
9.6.2.1 Demographic, Anthropometric and Anamnestic Data at Screening 59
9.6.2.2 Laboratory Parameters at Screening 59
9.6.3 Assessment of Efficacy/Performance 60
9.6.3.1 Blood Glucose Control Parameters 60
9.6.3.2 Lipid Levels 60
9.6.3.3 Atherogenic Index, Atherogenic Coefficient, Cardiac Risk Ratio 60
9.6.3.4 Body Weight Measurement – On Site 61
9.6.3.5 Body Weight Measurement – Off Site 61
9.6.3.6 Body Composition 61
9.6.3.7 Waist/Hip Circumference 61
9.6.3.8 Sagittal Abdominal Diameter 61
9.6.3.9 Short Form Health Survey 61
9.6.4 Assessment of Safety 61
9.6.4.1 Adverse Events and Device Deficiencies 61
9.6.4.2 Blood Pressure/Pulse Rate 62
9.6.4.3 Central Laboratory Parameters 62
9.6.4.3.1 Safety Parameters 62
9.6.4.3.2 Vitamins and Minerals 62
9.6.5 Other Parameters 62
9.6.5.1 Stool Sample Analyses 62

DocuSign Envelope ID: DC97F66E-B279-4A14-AC63-5C291FAB6C7D

9.6.5.2 Rating of Subject Satisfaction and Ease of Use 63
9.6.6 Comment on Methodology Deviating from Normal Clinical Practice 63
9.7 Adverse Events and Device Deficiencies 64
9.7.1 Definitions 64
9.7.2 Collecting and Recording of AEs and DDs 67
9.7.3 Assessment of Severity/Intensity 67
9.7.4 Assessment of Causal Relationship 68
9.7.5 Assessment of Outcome 69
9.7.6 Reporting of SAE/SADE and DDs with SADE potential 70
9.7.6.1 Reporting by the Investigator 70
9.7.6.2 Reporting to Regulatory Authorities 71
9.7.6.3 Reporting to Ethics Committees 72
9.7.7 Follow-up Period after an AE 72
9.7.8 Procedures in Case of Pregnancy 72
9.8 Data Quality Assurance 73
9.8.1 CIP Adherence 73
9.8.2 Monitoring 73
9.8.3 Audits and Inspections 74
9.8.4 Case Report Forms 74
9.8.5 Source Data 74
9.8.6 Training of Investigation Staff 74
9.9 Statistical Methods and Determination of Sample Size 75
9.9.1 Statistical Analysis Plan 75
9.9.2 Sample Size Calculation 75
9.9.3 Analysis Sets 76
9.9.4 General Principles 76
9.9.5 Primary Endpoint 77
9.9.6 Main Secondary Endpoint 77
9.9.7 Further Secondary Endpoints 78
9.9.8 Exploratory Endpoints 78
9.9.9 Analysis of Safety and Tolerability 78

DocuSign Envelope ID: DC97F66E-B279-4A14-AC63-5C291FAB6C7D

9.9.10 Treatment Compliance 79
9.9.11 Interim Analysis 79
9.9.12 Subgroup Analysis 79
9.9.13 Sensitivity Analyses 79
9.10 Data Management 79
10 DEVICE ACCOUNTABILITY 81
11 EMERGENCY PROCEDURES 82
12 MANAGEMENT OF THE INVESTIGATION 83
12.1 CIP Amendments 83
12.2 Discontinuation of the Investigation 83
12.2.1 Withdrawal of Subjects 83
12.2.2 Study Discontinuation Criteria 84
12.3 Reporting and Publication of Investigation Results 84
12.4 Disclosure and Confidentiality 84
12.5 Archiving 85
12.6 Insurance 85
12.7 Financing and Agreements 85
13 REFERENCES 87
14 APPENDICES 90
14.1 Signature Pages 90
14.2 Declaration of Helsinki 92
14.3 Short Form 12 Health Survey Questionnaire 93

LIST OF TABLES

Table 9.1 Schedule of events 42
Table 9.2 Composition of the IMD 50
Table 9.3 Composition of the placebo 51

2 SYNOPSIS

**Title of the clinical investigation**<br><br>Randomized, double-blinded, placebo-controlled, multicenter trial investigating performance and safety of the medical device SiPore21® in obese or overweight subjects with elevated blood glucose levels		
**Investigation code**<br><br>SITH/001921	**Investigational Medical Device (IMD)**<br><br>SiPore21®	**IMD classification**<br><br>Class IIb
**Reference number of the clinical investigation (CIV-ID)**<br><br>TBD		
**Intended use**<br><br>The purpose of SiPore21® is to reduce blood glucose levels (as measured by Glycated Hemoglobin [HbA1c] levels) and improve blood sugar control.<br><br>The SiPore21® is intended for self-treatment.		
**Sponsor**<br><br>Sigrid Therapeutics AB<br>C/O 7A Odenplan<br>Norrtullsgatan 6, plan 5<br>SE-113 29 Stockholm, Sweden		
**Manufacturer**<br><br>Sigrid Therapeutics AB<br>C/O 7A Odenplan<br>Norrtullsgatan 6, plan 5<br>SE-113 29 Stockholm, Sweden		
**Coordinating Investigator**<br><br>Kirsi Pietiläinen, MD, Professor, University of Helsinki		
**Time period for the clinical investigation**<br><br>Estimated date of first subject enrolled: Q3 2023<br><br>Estimated date of last subject completed: Q3 2024		

Design of the clinical investigation

Randomized, double-blinded, placebo-controlled, multicenter clinical investigation according to Medical Device Regulation article 62

Objectives and endpoints

**Primary objective**	**Primary endpoint**
To evaluate if the IMD treatment leads to a greater reduction in HbA1c level (relative to baseline) in comparison to placebo, in obese or overweight subjects with elevated blood glucose levels.	Difference in changes in HbA1c levels from V2 (baseline) to V4 (week 12) between IMD treatment group and placebo group.
**Main secondary objective**	**Main secondary endpoint**
To evaluate if the IMD treatment leads to a greater reduction in body weight (relative to baseline) in comparison to placebo.	Difference in changes in body weight assessed on-site from V2 (baseline) to V4 (week 12) between IMD treatment group and placebo group.
**Further secondary objectives**	**Further secondary endpoints**
To evaluate if the IMD treatment leads to a greater reduction in Homeostasis Model Assessment for Insulin Resistance (HOMA-IR), lipid levels, fasting blood insulin (FBI) and glucose (FBG) levels, sagittal abdominal diameter (SAD), waist-hip-ratio, body composition, and 12 Items Short Form Health Survey (SF-12) (all relative to baseline) in comparison to placebo.	Difference between IMD treatment group and placebo group in changes from V2 (baseline) to V4 (week 12) in:<br>- HOMA-IR<br>- Total cholesterol (TC)<br>- FBI<br>- FBG<br>- Low Density Lipoprotein Cholesterol (LDL-C)<br>- SAD<br>- Triglyceride (TG)<br>- Waist-hip-ratio<br>- Body fat content/mass, fat free mass (assessed by bioelectrical impedance analysis [BIA])<br>- SF-12
To evaluate the effect of the IMD treatment on body weight in comparison to placebo after treatment has ended.	Difference between IMD treatment group and placebo group in changes in body weight assessed off site from V2

	(baseline) to V4 (week 12) and to PC3 (week 13).
**Safety objective**	**Safety endpoints**
To assess the clinical safety of the IMD treatment in comparison to placebo.	Assessment of adverse events (AEs) (incidence, seriousness, outcome) throughout the study, comparison between IMD treatment and placebo.
	Assessment of adverse device effects (ADEs) throughout the study, post V2, comparison between IMD treatment and placebo.
	Assessment, including difference between IMD treatment and placebo in changes from V2 (baseline) to V3 (week 6) and to V4 (week 12), of:<br>- systolic/diastolic blood pressure and pulse rate.
	Assessment, including difference between IMD treatment and placebo in changes from V2 (baseline) to V4 (week 12), of:<br>- safety laboratory parameters (blood count, liver, and renal function parameters).
	Assessment of device deficiencies (DDs) throughout the study treatment period.
	Assessment, including difference between IMD treatment and placebo in changes from V2 (baseline) to V4 (week 12) of:<br>- vitamin B12 (cobalamin) and vitamin D<br>- trace elements Mg (magnesium) and Zn (zinc).
**Explorative objectives**	**Explorative endpoints**

To evaluate the effect of the IMD treatment on HbA1c levels, body weight assessed on site, HOMA-IR, lipid levels, FBI, FBG levels, SAD, waist-hip-ratio, body composition in comparison to placebo.	Difference between IMD treatment group and placebo group in changes from V2 (baseline) to V3 (week 6) in:<br>- HbA1c levels<br>- Body weight assessed on site<br>- HOMA-IR<br>- TC<br>- FBI<br>- FBG<br>- LDL-C<br>- SAD<br>- TG<br>- Waist-hip-ratio<br>- Body fat content/mass, fat free mass (assessed by BIA)
To evaluate the effect of the IMD treatment on Homeostasis Model Assessment for Beta Cell Function (HOMA-B), Quantitative insulin sensitivity check index (QUICKI), lipid levels, cardiometabolic risk indices, waist (WC) and hip circumference (HC) in comparison to placebo.	Difference between IMD treatment group and placebo group in changes from V2 (baseline) to V3 (week 6) and to V4 (week 12) in:<br>- HOMA-B, QUICKI<br>- Very Low-Density Lipoprotein Cholesterol (VLDL-C), High Density Lipoprotein Cholesterol (HDL-C), non-HDL-C<br>- Atherogenic index of plasma (AIP), atherogenic coefficient (AC) and cardiac risk ratio (CRR) 1 and 2<br>- WC and HC<br>- BMI
To evaluate the effect of the IMD treatment on high sensitivity C-Reactive Protein (hs-CRP) in comparison to placebo.	Difference between IMD treatment group and placebo group in changes from V2 (baseline) to V4 (week 12) in hs-CRP levels.
To assess subject satisfaction and the ease of use of study treatment.	Assessment of subject satisfaction and ease of use of study treatment at V4 (week 12).

To evaluate the microbiome diversity in stool samples.	Explore the differences in intestinal flora composition at V2 (baseline) and V4 (week 12) and content between IMD treatment group and placebo group.

Number of subjects planned to be randomized

288 1:1 IMD/placebo

Diagnosis and main eligibility criteria

Inclusion criteria:

1. Male or female 18-70 years old
2. HbA1c level ≥42 to ≤58 mmol/mol (≥6 to ≤7.5% - according to Diabetes Control and Complications Trial [DCCT, 1987]) at V1

   For Poland only: HbA1c level ≥42 to ≤53 mmol/mol (≥6 to ≤7% - according to the Official Journal of the Diabetes Poland, 2023 Vol. 3 Issue 1) at V1
3. Body mass index (BMI) >25 kg/m2 and ≤40 kg/m2
4. Regular intake of 3 main meals (self-reported)
5. Readiness and ability to:
   a. use the study treatment as recommended and attend all scheduled visits
   b. comply with all further study procedures
6. Readiness to maintain the current diet and level of physical activity during the study
7. Readiness not to participate in another clinical study during this study
8. Women of childbearing potential: commitment to use medically recognized contraception methods during the treatment period
9. Written informed consent by the participant following written and oral information by the investigator regarding nature, purpose, consequences and possible risks of the clinical study

Exclusion criteria:

1. Known allergy or hypersensitivity to the components of the IMD or placebo (self-reported)

2. Type 1 diabetes (T1D)/Latent Autoimmune Diabetes in Adult or secondary diabetes (self-reported)
3. Uncontrolled hypertension (regularly >179/109 mmHg [self-reported] and as per investigator's judgement based on screening procedures at V1)
4. History (self-reported) of myocardial infarction or stroke 6 months prior to V1
5. Clinically relevant abnormal electrocardiogram (ECG) at V1
6. History (<3 years prior to V1) or presence (self-reported) of:
    a. exocrine pancreatic insufficiency, chronic pancreatitis
    b. chronic inflammatory bowel disease, celiac disease
    c. diverticulosis (usually affecting the large intestine), adhesions, chronic constipation
7. State after pancreatic head resection with the need for additional intake of pancreatic enzymes (self-reported)
8. Major surgery of esophagus, stomach, intestine including colon which took place <3 years prior to V1, or >3 years prior to V1 in case of related current clinical symptoms (self-reported)
9. Clinically significant deviation, based on investigators judgment, in blood laboratory values at V1 of blood status (hemoglobin, erythrocytes, platelets, leucocytes, reticulocytes), kidney parameters (creatinine, cystatin C and estimated glomerular filtration rate), thyroid hormone status: thyroid-stimulating hormone (TSH)
10. Deviation in blood laboratory values at V1 of liver parameters (aspartate aminotransferase [ASAT], alanine transaminase [ALAT], alkaline phosphatase and γ-glutamyl transpeptidase [Gamma-GT]) that is clinically significant based on investigators judgment
11. Blood donation/other major blood loss or blood transfusion, that may interfere with the study as per investigator's judgment, within 56 days prior to V1 and any blood donation or transfusion during the study
12. Previous or current metformin or other medical anti-diabetic treatment or blood glucose levels reducing/influencing treatment/supplementation within 30 days prior to V1 and during the study
13. Current treatment/supplementation for weight management (e.g., fat binder/burner, carb blocker, satiety products) or known to influence weight (e.g., systemic corticosteroids)
14. Medical conditions that require medications taken during meals

15. Extreme diet form (e.g., ketogenic, very low carbohydrate) during the last 3 months prior to study
16. Self-reported regular average consumption of >1 L/day total of sugary beverages (e.g., soft drinks, fruit juices, energy drinks) and/or >200 g/day total of food based on simple sugar(s) between meals (e.g., commercial candies, dried fruit)
17. Pregnancy, lactation or active planning to achieve pregnancy
18. History of or current abuse of drugs, alcohol or medication
19. Any severe diseases/disorder (e.g., chronic kidney disease, neoplastic disease or psychiatric disorder) which may interfere with the compliance to the study procedures as per investigator's judgement
20. Participation in another study during the last 30 days prior to V1
21. Belonging to a vulnerable population, having any condition or other reason which in the opinion of the investigator would confound the conduct of the study or interpretation of the study results
22. Relative of the investigator or an employee at the clinical study site and Sponsor

Overall investigational design

The present clinical investigation is a randomized, double-blinded, placebo-controlled, multicenter international trial, planned to be conducted in Poland, Romania and Slovakia. The investigation will be performed to establish clinical evidence regarding the performance and safety of the IMD and is aiming at evaluating the suitability of the IMD for the intended purpose and population.

Investigational Medical Device, dosage and mode of administration

IMD:

The IMD is SiPore21® (with Mesoporous Silica Particles 21 [MSP21] as principal component). It is a pre-market Class IIb medical device with the intended use/purpose to improve blood glucose control, as measured by a reduction in HbA1c.

Placebo:

The placebo contains maltodextrin in place of the principal component.

One stick pack with 30 mL gel, to be orally consumed, containing the assigned study treatment, should be taken daily at the start of each of the three main meals (with the first bite of each respective meal, preferably after having chewed [if solid food] and swallowed the first bite of the meal). Subjects are instructed to drink one glass of water (at least 200 mL) with each main meal when the IMD is taken.

Total daily dose: 3 stick packs (1 x 3 main meals).

Duration of exposure to the Investigational Medical Device

12 weeks of study treatment

Duration of subjects' involvement in the investigation

Approximately 16-18 weeks

Visit schedule

- V1 (Day -14 to Day -5): Screening
- V2 (Day 0): Baseline/randomization/start of study treatment
- PC1 (Day 21 ± 3): Phone call at 3 weeks ±3 days after V2
- V3 (Day 42 ± 3): Intermediate control visit at 6 weeks ±3 days after V2
- PC2 (Day 63 ± 5): Phone call at 9 weeks ±5 days after V2
- V4 (Day 84 ± 5): Final visit, end of study treatment at 12 weeks ±5 days after V2
- PC3: Follow-up phone call at 7-10 days after V4
- PC4: Follow-up phone call at 21-35 days after V4

Clinical performance and safety assessments

- Demographic and anamnestic data (incl. medical history/concurrent treatment) collection
- Physical examination
- 12 lead ECG
- Pregnancy test (urine) for women of childbearing potential
- Blood draws for HbA1c, fasting glucose, fasting insulin, hs-CRP and lipid parameters (TG, TC, LDL-C, HDL-C)
    - Calculation of VLDL-C and non-HDL-C
    - Calculation of HOMA indices (HOMA-IR and HOMA-B) as well as QUICKI
- Blood draws for safety laboratory parameters (blood count, liver and renal function parameters) and TSH
- Blood draw for storage for analyses of vitamin/mineral levels
- Stool sample collection for analyses of gut microbiome parameters
- Measurements of body weight (on site by the site staff, off site by the subjects – instructions to be provided to subjects and in Informed Consent Form [ICF])
- Body height measurements, BMI assessment

- Body composition assessment (BIA) – body fat content/mass, fat free mass
- Waist and HC measurements, SAD
- SF-12
- Assessment of final treatment compliance (accountability based on provided / unused product)
- Blood pressure/pulse rate measurements
- Concurrent treatment(s) (e.g., other medical devices, medication, any natural health products including food supplements etc.)
- Assessment of AEs/ADEs
- Assessment of DDs
- Assessment of subject satisfaction and ease of use of study treatment

For all time points, please refer to the schedule of events.

Statistical methods

The primary objective is to evaluate if the IMD leads to a reduction of HbA1c (related to baseline) in comparison to placebo.

The primary endpoint is defined as the difference in changes in HbA1c levels at V4 (week 12) compared to V2 (baseline), between IMD and placebo group. A higher reduction of HbA1c (related to baseline) in comparison to placebo corresponds to a better clinical performance.

The primary analysis will be conducted using a two-sided test with α = 0.05 (significance level α=5%), using the following hypotheses:

- H_0: $\mu_{active} = \mu_{placebo}$, i.e., no difference between IMD and placebo control group with respect to the primary endpoint,
- H_A: $\mu_{active} \neq \mu_{placebo}$, i.e., difference between IMD and placebo control group with respect to the primary endpoint,

where H_0 is the null hypothesis and H_A is the alternative hypothesis.

μ_{active} and $\mu_{placebo}$ are,
μ_{active} = expected change in HbA1c levels at V4 (week 12) compared to V2 (baseline) for the active group treated with IMD,
$\mu_{placebo}$ = expected change in HbA1c levels at V4 (week 12) compared to V2 (baseline) for the control group treated with placebo.

The analysis will be conducted using an Analysis of Covariance (ANCOVA) model. The model will include baseline HbA1c at V2 as covariate and treatment group, BMI at V1 (<30 or ≥ 30 kg/m2), HbA1c level at V1 (<48 or ≥48 mmol/mol) and site as factors. The two-sided p-value will be considered statistically significant if it is below 5%.

The primary analysis will be conducted on both Full Analysis Set (FAS) and Per-Protocol (PPS) populations. The FAS is considered the main analysis while the PPS is only supportive.<br>All statistical analyses proposed in the CIP and the SAP will be reported in the CIR, regardless if the primary endpoint reaches significance.<br>Safety evaluations will be based on AEs, ADEs, DDs, safety laboratory parameters, vital signs, physical examinations, and assessment of serum levels of vitamin and minerals using Safety set (SS) population.<br>Subgroup analyses will be performed for the primary endpoint and the main secondary endpoint using FAS.<br>A Statistical Analysis Plan (SAP) will be finalized prior to the database lock of the study. The SAP contains a detailed description of all statistical methodology utilized.
**Conformity to good clinical practice**<br>This clinical investigation will be performed in accordance with the Medical Device Regulation (EU) 2017/745 (MDR), ISO 14155:2020, Good Clinical Practice (GCP), applicable local regulations and the ethical principles that have their origin in the Declaration of Helsinki.

3 LIST OF ABBREVIATIONS AND DEFINITION OF TERMS

AE	Adverse Event
AC	Atherogenic Coefficient
ADA	American Diabetes Association
ADE	Adverse Device Effect
AIP	Atherogenic Index of Plasma
ALAT	Alanine Transaminase
ANCOVA	Analysis of Covariance
ASADE	Anticipated Serious Adverse Device Effect
ASAT	Aspartate Aminotransferase
BIA	Bioelectrical Impedance Analysis
BMI	Body Mass Index
CDP	Clinical Development Plan
CE	European Conformity ("Conformité Européenne")
CEP	Clinical Evaluation Plan
CIP	Clinical Investigation Plan
CRI	Castelli's Risk Index
CRO	Contract Research Organization
CRR	Cardiac Risk Ratio
DCCT	Diabetes Control and Complications Trial
DD	Device Deficiency
DPP	Diabetes Prevention Program
EC	Ethics Committee
ECG	Electrocardiogram
eCRF	Electronic Case Report Form
EFSA	European Food Safety Authority
EMA	European Medicines Agency
EN	European Norm
ES	Enrolled Set
EU	European Union
FAS	Full Analysis Set
FBG	Fasting Blood Glucose
FBI	Fasting Blood Insulin
FDA	Food and Drug Administration
FPG	Fasting Plasma Glucose
Gamma-GT	y-glutamyl transpeptidase

GCP	Good Clinical Practice
GI	Gastrointestinal
HACCP	Hazard Analysis and Critical Control Points
HbA1c	Glycated Hemoglobin
HC	Hip Circumference
HDL	High Density Lipoprotein
HDL-C	High Density Lipoprotein Cholesterol
HOMA	Homeostasis Model Assessment
HOMA-IR	Homeostasis Model Assessment for Insulin Resistance
HOMA-B	Homeostasis Model Assessment for Beta Cell Function
hs-CRP	High Sensitivity C-Reactive Protein
IB	Investigator's Brochure
ICF	Informed Consent Form
IFU	Instructions For Use
IMD	Investigational Medical Device
ISF	Investigator Site File
ISO	International Organization for Standardization
IWRS	Web-based Randomization System
LDL-C	Low Density Lipoprotein Cholesterol
MDCG	Medical Device Coordination Group
MDR	Medical Device Regulation (EU) 2017/745 MDR
MedDRA	Medical Dictionary for Regulatory Activities
Mg	Magnesium
mmHg	Millimeters of mercury
MSP21	Mesoporous Silica Particles 21
PC	Phone Call
PG	Plasma Glucose
PPS	Per-Protocol Set
PT	Preferred Term
QUICKI	Quantitative Insulin Sensitivity Check Index
RS	Randomized Set
SAE	Serious Adverse Event
SAD	Sagittal Abdominal Diameter
SADE	Serious Adverse Device Effect
SAP	Statistical Analysis Plan
SF-12	Short Form Health Survey (12-Item)
SiPore21®	Gel containing MSP21

SOC	System Organ Class
SOP	Standard Operating Procedure
SS	Safety Set
T1D	Type 1 Diabetes
T2D	Type 2 Diabetes
TC	Total Cholesterol
TG	Triglycerides
TMF	Trial Master File
TSH	Thyroid-Stimulating Hormone
USADE	Unanticipated Serious Adverse Device Effect
V	Visit
VLDL-C	Very Low-Density Lipoprotein Cholesterol
WC	Waist Circumference
WHO	World Health Organization
Zn	Zinc

4 ETHICAL AND REGULATORY REQUIREMENTS

4.1 Regulatory Aspects

This clinical investigation will be performed in accordance with the principles of the World Medical Association (Declaration of Helsinki, version applicable for the involved investigators), the Medical Device Regulation (EU) 2017/745 MDR, ISO 14155:2020, GCP and applicable local regulations.

4.2 Independent Ethics Committee/Regulatory Authority

Prior to start of the clinical investigation and prior to implementation of any substantial amendment, the following conditions must be fulfilled at each study site:

- favorable documented opinion for the study must be obtained from the responsible ethics committee (EC)
- required documented approval must be obtained by the respective regulatory authority

Any additional requirements imposed by the EC or regulatory authority shall be followed, as appropriate.

4.3 Subject Information and Informed Consent Form

The subject must declare informed consent before participation in the clinical investigation, i.e., before any study specific procedure is performed.

The investigator will ensure that the subject is given full and adequate oral and written information (subject information) about the nature, purpose, consequences and potential risks of the clinical investigation. Subjects must be informed that they are free to withdraw from the study at any time without giving any reason for withdrawal and without any resulting disadvantages, how personal and health-related data will be collected and used during the study, and that their identity and medical information will not be disclosed. The subject should be given the opportunity to ask questions and should be allowed ample time to consider the information provided. The subject’s signed and dated informed consent must be obtained before conducting any study specific procedure. The investigator(s) must store the original, signed and dated ICFs in the investigator site file (ISF). A copy of the signed and dated ICF must be given to the subject.

If new information becomes available that can significantly affect a subject’s future health and medical care, that information will be provided to the subject(s) affected in written form. If relevant, all affected subjects will be asked to confirm their continuing informed consent in writing.

4.4 Investigator's Obligations

By signing this document, the investigator confirms to adhere to the Clinical Investigation Plan (CIP) and all applicable national and international regulations and guidelines. The investigator has to ensure that all other study (sub-) investigators and study personnel assisting in the present clinical investigation are qualified by training for their individual responsibilities and functions and informed about the clinical study documents, about the IMD, and the investigational procedures.

A delegation log will be set up and updated during the course of the study, in which the investigator will list all study team members including their delegated responsibilities, start and stop dates.

The investigators will permit access to source data for study-related monitoring as well as in case of audits and inspections, if applicable.

4.5 Data Protection and Confidentiality

The processing of personal data in the study follows the EU General Data Protection Regulation 2016/679 (GDPR).

The Sponsor will be the data controller for the study, responsible for safeguarding participant's personal data collected during the study. Participating study sites and other vendors assisting the Sponsor with the study (such as Contract Research Organizations [CROs]) will process data on behalf of the Sponsor.

Following consent to participation in the clinical investigation, the subject will be assigned a screening number. Subjects meeting the eligibility criteria will be assigned a randomization number. The collected data will be made available to the CRO and the study's Sponsor only in pseudonymous form, to minimize the chances of matching the data to an individual person. Only the age and gender will be recorded in the electronic Case Report Form (eCRF) (no initials, no date of birth). The paper-based identification list will be confidentially retained by the investigator.

In case of serious adverse event (SAE) related documentation provided to the CRO and the study Sponsor, the investigator will ensure the pseudonymity of the copies of the documentation provided.

In the event of a breach of data, the Sponsor should be notified within 24 hours of the discovery of the breach, and the data protection officer will be notified immediately. The data protection officer will report serious breaches to the responsible authority, and further corrective and preventive actions (CAPA) will be initiated accordingly. Procedures defined in the Regulation European Union 2016/679 (GDPR) regarding notification of a data breach to the supervisory authority and the affected study subjects (Article 33) will be followed. Specifically, without undue delay and, where feasible, the supervisory authority will be notified not later than 72 hours after the Sponsor has become aware of the breach.

With the receipt of the CIP, the investigator is bound to treat all information contained herein as strictly confidential. He/she is further required to inform his/her support staff or other personnel that may have access to the CIP of this confidentiality.

5 RISKS AND BENEFITS OF THE INVESTIGATIONAL MEDICAL DEVICE AND CLINICAL INVESTIGATION

5.1 Anticipated Clinical Benefit of the IMD

When used as intended, improvement of blood glucose control is anticipated within the period of treatment with the IMD.

5.2 Anticipated Adverse Device Effects

During or after the use of the IMD, the following side effects might be observed:

- Frequent bowel movement
- Flatulence, diarrhea, abnormal feces/hard feces, constipation
- Abdominal distention/discomfort/pain
- Oral pain/discomfort or dry mouth
- Hematochezia due to constipation/harder stool
- Dyspepsia, nausea, vomiting
- Increased urinary frequency

Contraindications, warnings, and precautions related to residual risks are included in the Instructions For Use (IFU).

Of note, no serious adverse device effects (SADEs) and one SAE (preferred term [PT]: macular degeneration) were documented in a preceding clinical investigation, STAR01, with the IMD predecessor SiPore15®.

5.2.1 Risk Associated With the IMD

As per the latest version of the Investigator’s Brochure (IB), the specific risks have been handled according to EN ISO 14971:2019, with the conclusion that the benefits outweigh the risks. The risk evaluation reveals no unacceptable residual risks.

As the IMD is known to adsorb water, insufficient liquid intake may lead to hard feces and constipation. To mitigate this risk, the respective information is included in the IFU stating that subjects should drink one glass of water (at least 200 mL) with each main meal when the IMD is taken.

Since the available data concerning the use of the IMD do not yet allow a final conclusion on any impact of the IMD on adsorption of essential micronutrients, serum levels of trace elements (Mg, Zn) and vitamins (B12, D) will be monitored during the study in order to reveal any deficiencies.

DocuSign Envelope ID: DC97F66E-B279-4A14-AC63-5C291FAB6C7D

5.2.2 Risks due to Manufacturing Errors

Manufacturing risks in regard to the device safety have been evaluated in agreement with HACCP (Hazard analysis and critical control points) for food supplement production. To mitigate the risks, the production is monitored by means of a quality management system ensuring that the quality of the manufactured product complies with the specified standards for food safety.

5.2.3 Risks due to Improper Use Including Overdose

To mitigate the risk of improper use including overdose, recommendations for dosing and frequency of administration and dosing intervals for the intended use based on previous clinical experience are defined in the IFU. The IFU will be provided to the subjects, specifying details on proper use and general and specific precautions. Further, the labeling on product packaging states the expiry date and proper storage requirements (see section 9.5.3).

As per risk management assessment, concerning the specific risk of overdose, the respective information is included in the IFU stating not to take more than the quantity indicated and that overdosing may lead to constipation.

Further, to prevent any drying out and/or contamination of the product, the respective information is included in the IFU requiring immediate intake upon opening of the stick pack with study treatment.

5.2.4 Risks due to Interactions with Pharmaceutical Products

As per the IFU, to avoid a possible impact on the absorption of some medications, the subjects will be instructed to take the IMD at least 2 hours before or after taking any concurrent medication.

Medications taken during meals are not allowed during the study.

5.2.5 Overall Risk Assessment

According to the risk management assessment for the IMD, all identified risks are judged to have been reduced as far as possible and no unacceptable residual risks have been identified.

5.3 Risks Associated with the Participation in the Clinical Investigation

Participation in the investigation is not deemed to pose any specific risks to subjects. The principal clinical assessments in the study are standard practice in health care applied to assessment of blood glucose control. The study procedures will be performed by qualified and appropriately trained investigators and site staff.

For prediabetic subjects, guidelines do not recommend drug therapy. Instead, lifestyle changes are advised. Diabetologists and experienced key opinion leaders in the field have deemed that the health risk of delaying lifestyle advice is low for the subjects as this is a

short-term study and patients are under supervision of physicians. Lifestyle advice will be provided once the prediabetic subjects exit the investigation.

For non-treated Type 2 diabetes (T2D) subjects there is an additional risk in delaying start of standard of care drug treatment by 12 weeks. In the study, their blood glucose levels and vital signs will be monitored to ensure safety of the subjects. All T2D subjects will be informed according to the guidelines before being provided with the opportunity to enroll in the investigation, if the investigator deems the enrolment appropriate. In addition, the subjects will be informed of anticipated symptoms so that if they experience these, they may contact their healthcare provider. When the T2D subjects exit the investigation, they will be referred to care according to national guidelines. This has been deemed adequate in discussions with diabetologists and experienced key opinion leaders in the field.

It is anticipated that pathologies are detected during screening based on the eligibility criteria and during the investigation, including high HbA1c, high blood pressure, and lipids outside normal range. The investigator is responsible for assessing if any subject needs additional medical attention and should be withdrawn from the investigation. Subjects will, whenever possible, be seen and assessed by the investigator and be medically advised as appropriate e.g., referral to general practitioner.

From a patient perspective, the risks described in the informed consent and subject information are as written below:

- Only for the newly diagnosed T2D; you will be asked to delay drug treatment of your diabetes for 12 weeks to test the IMD. Your blood sugar will be monitored, and you will receive information of symptoms to look out for. If you experience these symptoms contact your healthcare provider. When you exit the study, you will be provided with lifestyle advice.

- During or after the use of SiPore21®, the following side effects might be observed:

  Frequent bowel movement, flatulence, diarrhea, abnormal feces/hard feces, constipation, abdominal distention/discomfort/pain, oral pain/discomfort or dry mouth, hematochezia (blood in the stool) due to constipation/harder stool, dyspepsia (heart burn), nausea, vomiting, increased urinary frequency

  Measures to ensure your safety will be taken at the study site.

- There may be unforeseen risks associated with the use of the IMD. If new relevant information about the safety of the IMD is discovered during the study, you will be informed about it. You may then decide whether you wish to continue participating in the study.

DocuSign Envelope ID: DC97F66E-B279-4A14-AC63-5C291FAB6C7D

5.4 Rationale for Benefit-Risk Ratio

The current overall risk assessment reveals that, when used as intended, the IMD does not pose an unjustifiable risk.

The IMD has the potential to reduce cardio-metabolic risk factors in the prediabetic population through a gentle mode of action with no systemic exposure. Also, in the T2D population, the IMD has the potential to reduce cardio-metabolic risk factors with a safe and local effect.

Thus, the potential benefit of the IMD treatment is expected to outweigh its potential risks.

6 INVESTIGATOR(S) AND INVESTIGATION ADMINISTRATIVE STRUCTURE

This clinical investigation is sponsored by Sigrid Therapeutics AB. Key members of the Sponsor, CRO and sub-contractors are presented below.

**Sponsor**	**Sponsor's Signatory**<br><br>Name: Sana Alajmovic<br>Address: Sigrid Therapeutics AB<br>C/O 7A Odenplan<br>Norrtullsgatan 6, plan 5<br>SE-113 29 Stockholm, Sweden<br>Phone: +46 723 893 396<br>E-mail: sana@sigridthx.com
	**Sponsor Medical Officer**<br><br>Name: Stephan Rössner<br>Address: Apple bay obesity research centre<br>Snäckparken 7<br>SE- 167 53 Bromma, Sweden<br>Phone: +46 704 150 224<br>E-mail: stephan@rossner.se
	**Clinical Project Manager**<br><br>Name: Maria Klockare<br>Address: Sigrid Therapeutics AB<br>C/O 7A Odenplan<br>Norrtullsgatan 6, plan 5<br>SE-113 29 Stockholm, Sweden<br>Phone: +46 706 232 505<br>E-mail: maria@sigridthx.com
**Investigational site(s)**<br>**(See separate list)**	**Coordinating Investigator**<br><br>Name: Kirsi Pietiläinen, MD, Professor<br>Address: University of Helsinki<br>Haartmaninkatu 8<br>FI-00014 Helsinki, Finland<br>Phone: +35 850 599 2295<br>E-mail: kirsi.pietilainen@helsinki.fi

DocuSign Envelope ID: DC97F66E-B279-4A14-AC63-5C291FAB6C7D

**CRO**	HungaroTrial CRO<br>Address: 89-95. Fehervari ut<br>1119 Budapest, Hungary<br>Email: mpetho@hungarotrial.com
**Vigilance**	HungaroTrial CRO Address: 89-95. Fehervari ut<br>1119 Budapest, Hungary<br>Email: HTsafety@hungarotrial.com
**Statistics**	SDS Life Science AB<br>Address: Sankt Eriksgatan 113,<br>113 43 Stockholm, Sweden<br>Email: daniel.bruce@sdslifescience.com
**Data Management**	CRScube, Inc.<br>Address: #4F Hapjeong Office Building<br>19, Yanghwa-ro, Mapo-gu, Seoul (04027)<br>Republic of Korea
**Manufacturing, packaging and labelling of IMD**	Sigrid Therapeutics AB<br>C/O 7A Odenplan<br>Norrtullsgatan 6, plan 5<br>SE-113 29 Stockholm, Sweden

Signatures required are provided in Appendix 14.1.

7 INTRODUCTION

7.1 Background

Prediabetes, alternatively termed as "intermediate hyperglycemia", is characterized by blood glucose levels above the normal range but below diabetes diagnostic threshold (6.1-6.9 mmol/L). The recent inclusion of codes specifically listed 'prediabetes' as a separate billable condition (International Classification of Diseases, 10th Revision, Clinical Modification; ICD-10-CM), which distinguishes prediabetes from diabetes as an independent pathological condition. Prediabetes is associated with increased risk of developing other medical conditions, collectively termed metabolic syndrome, including obesity, hypertension, non-alcoholic fatty liver disease, hypertriglyceridemia, and cardiovascular diseases (Grundy, 2012). Approximately 25% of individuals with prediabetes will progress to overt T2D within 3–5 years, and as many as 70-90% will develop overt diabetes within their lifetime (Tabák et al., 2012). Therefore, maintaining blood glucose levels within the normal range is critical for preventing diabetes and its co-morbidities.

At present, approximately 537 million adults suffer from diabetes which caused 6.7 million deaths globally in 2021 (International Diabetes Federation, 2021). In addition, the global increase in the prevalence of prediabetes does not bode well for the growing epidemic of diabetes. According to the International Diabetes Federation Atlas (10th Edition, 2021), 541 million adults are living with prediabetes, and by 2045, the worldwide prevalence is estimated to affect 783 million individuals (11.4% of the global population). These figures, therefore, signify a need for preventive activities to reduce the ongoing progress of this disease and its associated complications.

With prediabetes being a high-risk state for developing T2D and its increasing prevalence worldwide, there are treatments available for blood sugar reduction in prediabetics and early Type 2 diabetics, such as Metformin and other agents which reduce blood sugar levels. The standard of care, recommended by physicians, to individuals with prediabetes is lifestyle interventions, which includes healthy diet and an increase in moderate physical activity. The largest clinical trial performed with prediabetes to date is the Diabetes Prevention Program (DPP; clinicaltrials.org, ID: NCT00004992). The DPP demonstrated that after 6 months of interventions, lifestyle modification lowered the HbA1c level and resulted in modest weight loss, which in turn reduced the incidence of diabetes in persons at high risk (Knowler et al., 2002). However, the resource-intensive lifestyle interventions implemented in the DPP trial are not only expensive but also complicated to implement and maintain. Furthermore, efforts to translate and implement diabetes prevention programs globally have been lagging. Due to these reasons, lifestyle interventions are not realistically applicable to a real-life setting, and even if it is applied, only approximately 10% of individuals adhere to a healthy lifestyle in practice (Schlesinger et al., 2020). This means that the majority of individuals with prediabetes are left without treatment. Metformin, the first-line pharmacological treatment for T2D, may be prescribed to individuals with prediabetes. However, metformin treatment is frequently associated with gastrointestinal (GI) side effects (20-30% of patients [Kirpichnikov et al., 2002]), and in approximately 5% of cases,

severe GI side effects develop which leads to the discontinuation of therapy (Florez et al., 2010, Bouchoucha and Cohen, 2011). Overall, it is evident that there is still an unmet clinical need within prediabetes which calls for safe and effective therapies, devices, and tools to help curb this growing public epidemic.

According to the International Expert Committee report (2009), HbA1c levels from 42 to <48 mmol/mol (6.0% to <6.5%) indicate the highest risk for progression to T2D. Prediabetes, as well as T2D, is identified by laboratory measurement of fasting plasma glucose (FPG) or, glycosylated hemoglobin (HbA1c) or 2-hour post-load blood glucose (2hBG). HbA1c is a broadly established measure of long-term blood glucose control (for the 2-3 months preceding the actual measurement). As per American Diabetes Association (ADA, 2022) the following thresholds apply for the prediabetes and T2D:

- Prediabetes:
  - HbA1c 39-47 mmol/mol (5.7-6.4%) or
  - FPG 100-125 mg/dL (5.6-6.9 mmol/L) or
  - 2hBG 140-199 mg/dL (7.8-11.0 mmol/L)
- T2D:
  - HbA1c 48 mmol/mol (≥6.5%) or
  - FPG ≥126 mg/dL (7.0 mmol/L) or
  - 2hBG ≥200 mg/dL (11.1 mmol/L) or
  - Random PG ≥126 mg/dL (7.0 mmol/L).

7.2 Investigational Medical Device

The IMD, SiPore21®, is a gel containing engineered mesoporous silica particles, named MSP21, as the principal component. IMD is taken in connection with a meal and acts locally in the GI tract. The functionality and performance of the IMD lies in the presence of pores in MSP21. MSP21 is carefully engineered to have pores with a specific diameter, which are larger than the size of digestive enzymes but smaller than undigested food. Due to this unique physical structure, the IMD acts as a molecular sieve, physically separating digestive enzymes from undigested and/or partly digested food in the GI tract. The physical separation of enzymes from food consequently reduces the breakdown of carbohydrates and fats, leading to lowering in total energy intake. This results in improved blood glucose control, as measured by a reduction in long-term blood glucose (HbA1c).

The IMD is targeted to be classified as a Class IIb medical device according to the classification rules of the European Medical Device Regulation (EU) 2017/745 (rules 5 and 21), intended to lower blood glucose levels in subjects with obesity or overweight.

7.3 Non-Clinical Summary

7.3.1 Biocompatibility and Toxicity

Assessment of the biocompatibility and potential toxicity of the IMD has been performed according to ISO 10993 and the relevant endpoints considered were: physical and chemical characterization, cytotoxicity testing, genotoxicity testing and testing for irritation and sensitization.

7.3.1.1 Physical and chemical characterization

The physical and chemical parameters of the IMD have been determined. In addition, an extensive systematic literature review has been performed and documented. In summary, all substances present in the IMD are approved for use in food. The information provided on ingredients and manufacturing is expected to fulfil the requirements providing the minimum physical and chemical information (ISO 10993-18).

7.3.1.2 Cytotoxicity

Cytotoxicity testing yielded no critical observations and the IMD safe without considerations, hence, the IMD is considered non-cytotoxic.

7.3.1.3 Genotoxicity

The IMD has been tested for mutagenicity in the "In vitro Bacterial Reverse Mutation Test" and no genotoxic activity was observed. The IMD was also tested under the conditions of the "Test for genotoxicity (in vitro micronucleus assay)" and did not cause genotoxic effects.

7.3.1.4 Irritation and Sensitization

The IMD has been submitted to irritation testing in rabbits and sensitization testing in mice, in order to test for potential immunoreactivity against the device or parts of the device.

The IMD did not cause skin irritation and was rated non-sensitizing.

7.3.2 Other Non-Clinical Data

During development of the IMD, the formulated gel was tested, and in some of the nonclinical tests only the principal component of SiPore21®, MSP21, have been tested.

In addition, data generated on SiPore15®, a predecessor of SiPore21®, is considered applicable. Please refer to the latest version of the IB for a detailed demonstration of equivalence.

The performances of SiPore15®, MSP21, and SiPore21® have been assessed *in vitro* in terms of their ability to adsorb pancreatic α-amylase and thereby reduce carbohydrate digestion (BY 2023-02-28 [internal report], Waara et al., 2020). Both MSP21 and SiPore21® are shown to efficiently adsorb α-amylase and reduce carbohydrate digestion, with similar effects for

DocuSign Envelope ID: DC97F66E-B279-4A14-AC63-5C291FAB6C7D

MSP21 and SiPore21®. SiPore15® and MSP21 are furthermore able to adsorb lipase and thereby reduce lipid digestion as shown in lipase adsorption assay using porcine pancreatic lipase.

In *ex vivo* studies, it has been demonstrated that SiPore15® is able to reduce the concentrations of digestive enzymes, with a concomitant reduction in carbohydrate/lipid digestion (Waara et al., 2020).

Furthermore, *in vivo* studies demonstrated that treatment of diet-induced obese mice with SiPore15® led to a decrease in body weight and fat composition (Kupferschmidt et al., 2014) and suppression of weight gain along with a positive effect on glucose tolerance (Rinde et al., 2020).

For further details on non-clinical studies, please refer to the latest version of the IB.

7.4 Clinical Experience

The safety, tolerability and performance of the predecessor SiPore15® has been confirmed in two clinical investigations.

In the first study, subjects with normal-weight or obesity were included to evaluate the tolerability, safety and feasibility of SiPore15® as a food additive in humans (Hagman et al., 2020; Waara et al., 2020).

The investigation was conducted in two parts:

- Part 1 lasted 3 weeks and all subjects participated. During Part 1, both subjects with normal weight (Group A) and obesity (Group B) received oral administration of treatment, involving a placebo run-in period for 5 days followed by an increasing dosage regiment of 3x1, 3x2 and 3x3 g (three doses per day) for 4, 5 and 7 days, respectively.

- Part 2 only included subjects with obesity from Group B. These received 3 daily doses of 3 g of SiPore15® for 10 additional weeks. In total, the obese participants consumed SiPore15® for 12 weeks.

Performance was studied by investigating potential effects on blood lipids, hormone levels and body weight. Analyses of blood included: levels of CRP, vitamin A and D, trace elements (Mg and Zn), blood lipids (cholesterol, triglycerides, HDL, LDL, ratio HDL/LDL, Apo A1 and Apo B), insulin, fasting glucose and HbA1c. In addition, weight and bioimpedance were measured.

Analysis of clinical chemistry variables did not raise any safety concerns. There were a few statistically significant changes but none that were considered clinically relevant (the values remained within a normal range). Vitamin A and D levels decreased significantly from baseline to Week 3 in Group A, however, no significant effect was seen in Group B after 12 weeks. This indicates that the changes are not clinically relevant. Compared to baseline, Zn levels were significantly lower in Group B at Week 3 and at Week 12. However, this was not

considered clinically relevant as the values remained within a normal range. Silicon was detected in urine, however with high individual variability. In the group consisting of subjects with obesity, no SAEs and no study discontinuations were reported. Adverse events (AEs) observed were mild and did not result in discontinuation. Subjects with obesity reported most AEs in the system organ class (SOC) 'infections and infestations' where 30% of participants reported at least one AE. In summary, the AEs reported were all mild and transient. The majority of the subjects in both groups reported unchanged stool frequency over the duration of the study. No symptoms compatible with fat malabsorption were noted. No blood was detected in the feces.

In the second study, subjects with prediabetes or newly diagnosed T2D were included to evaluate the performance and safety of SiPore15® after a 6 and 12-week long treatment period (Baek et al., 2021).

Performance was evaluated by measuring change in HbA1c and LDL-C by taking blood samples and for body fat levels by using a bioelectric impedance scale. Safety was evaluated by assessing the incidence and frequency of AEs and adverse device effects (ADEs).

Following the treatment period of 12 weeks, significant reductions in HbA1c (mean difference -1.4 mmol/mol (standard deviation: 1.5 mmol/mol); p = 0.0391), total cholesterol (p = 0.0330), and sagittal abdominal diameter (SAD; p = 0.0105) were observed. A few biochemistry assessments (Cystatin C, Vitamin B12 and platelet count) showed statistically significant changes after 12 weeks treatment, however these were within the normal ranges and were not considered clinically significant by the investigators. No SAE was observed over the 12-week treatment period and the majority of AEs recorded were mild. ADEs occurring in more than one out of ten subjects were abnormal feces, abdominal distension and hard feces.

For further details on clinical experience, please refer to the latest version of the IB.

7.5 Rationale for the Investigation

The proposed investigation is set out to be a randomized, double blind, placebo-controlled, multicenter study according to MDR Article 62 to investigate performance and safety of the medical device SiPore21® in subjects with obesity or overweight and elevated blood glucose levels.

8 INVESTIGATIONAL OBJECTIVES AND ENDPOINTS

The aim of this clinical investigation is to evaluate the clinical performance of the IMD for blood glucose control and its clinical safety. The endpoints selected to evaluate the effects of the IMD are based on the state-of-the-art assessments of blood glucose control (ADA, 2022).

8.1 Hypothesis

The main hypothesis is that treatment with SiPore21® for 12 weeks will reduce the HbA1c in obese or overweight subjects with elevated blood glucose levels. It is further hypothesized that treatment with SiPore21® for 12 weeks will result in a reduction in body weight and influence metabolic control parameters and lipid levels.

8.2 Primary and Secondary Objectives and Endpoints

**Primary objective**	**Primary endpoint**
To evaluate if the IMD treatment leads to a greater reduction in HbA1c level (relative to baseline) in comparison to placebo, in obese or overweight subjects with elevated blood glucose levels.	Difference in changes in HbA1c levels from V2 (baseline) to V4 (week 12) between IMD treatment group and placebo group.
**Main secondary objective**	**Main secondary endpoint**
To evaluate if the IMD treatment leads to a greater reduction in body weight (relative to baseline) in comparison to placebo.	Difference in changes in body weight assessed on-site from V2 (baseline) to V4 (week 12) between IMD treatment group and placebo group.
**Further secondary objectives**	**Further secondary endpoints**
To evaluate if the IMD treatment leads to a greater reduction in Homeostasis Model Assessment for Insulin Resistance (HOMA-IR), lipid levels, fasting blood insulin (FBI) and glucose (FBG) levels, sagittal abdominal diameter (SAD), waist-hip-ratio, body composition, and 12 Items Short Form Health Survey (SF-12) (all relative to baseline) in comparison to placebo.	Difference between IMD treatment group and placebo group in changes from V2 (baseline) to V4 (week 12) in:<br>- HOMA-IR<br>- Total cholesterol (TC)<br>- FBI<br>- FBG<br>- Low Density Lipoprotein Cholesterol (LDL-C)<br>- SAD<br>- Triglyceride (TG)<br>- Waist-hip-ratio

	- Body fat content/mass, fat free mass (assessed by bioelectrical impedance analysis [BIA])<br>- SF-12
To evaluate the effect of the IMD treatment on body weight in comparison to placebo after treatment has ended	Difference between IMD treatment group and placebo group in changes in body weight assessed off site from V2 (baseline) to V4 (week 12) and to PC3 (week 13).

8.2.1 Justification of Endpoints

**Parameters**	**Justification**
HbA1c	Golden standard used by International Diabetes Federation (IDF) (IDF, 2021) and World Health Organisation (WHO) (WHO, 2011) to screen for or diagnose prediabetes and T2D.<br>According to European Medicines Agency's (EMA) (EMA, 2018) Guideline on clinical investigation of medicinal products in the treatment or prevention of diabetes mellitus: "*Glycohaemoglobin (HbA1C) is the most widely accepted measure of overall, long-term blood glucose control in patients with diabetes. It reflects the mean glucose concentration over the past 2-3 months. Reduction of HbA1C is known to reduce the long-term risk of development of microvascular complications. Therefore, HbA1c is an appropriate primary endpoint to support a claim based on glycaemic control*".
Weight	- Strongly linked with prediabetes/ T2D prevalence/cause.<br>- Animal studies also showed significant reduction (Kupferschmidt et al., 2014; Rinde et al., 2020).
HOMA-IR	- Indicator of insulin resistance and is most widely used in clinical research.<br>- This parameter is more likely to show change as it takes both FBI and FBG into account.<br>- Decreasing trend was observed in the STAR trial (Baek et al., 2022).
TC	- TC level may also be high with prediabetes/ T2D due to high blood glucose level.<br>- Significantly reduced in the STAR trial (Baek et al., 2022).<br>- SiPore15® lowered blood lipid level in animals (May et al., 2022).
FBI	- Measure of insulin sensitivity.<br>- Impaired insulin sensitivity precedes glucose intolerance in the development of prediabetes and T2D.

Parameters	Justification
	- Decreasing trend was observed in the STAR trial (Baek et al., 2022).
FBG	- Used to diagnose prediabetes and T2D.<br>- SiPore21® is proposed to promote better control in blood glucose level in general.
LDL-C	- LDL-C level may also be high with prediabetes/T2D due to high blood glucose level.<br>- Significantly reduced in the FIM and STAR trial (Baek et al., 2022; Hagman et al., 2020).
SAD	- A measure of visceral fat depots.<br>- In combination with BMI, it is used as a "new" predictor of diabetes incidence.<br>- May also change in correlation to the changes in weight and WHR measures.<br>- Significantly reduced in the STAR trial (Baek et al., 2022).
TG	- High TG in prediabetes and T2D.<br>- May also change in correlation to the changes in weight.
WHR	- A good indicator for predicting prediabetes and T2D.<br>- May also change in correlation to the changes in weight and SAD measures.
BIA	- Body fat content may also change in correlation to the changes in weight, SAD, WHR measures.<br>- Animal studies showed significant reduction in body fat (Kupferschmidt et al., 2014; Rinde et al., 2020).
SF-12	SiPore21® may show improvements in one's physical and mental health.
Weight off site	To detect further changes after the SiPore21® treatment period.

8.3 Safety/Tolerability Endpoints

Safety objective	Safety endpoints
To assess the clinical safety of the IMD treatment in comparison to placebo.	Assessment of AEs (incidence, seriousness, outcome) throughout the study, comparison between IMD treatment and placebo.

**Safety objective**	**Safety endpoints**
	Assessment of ADEs throughout the study, post V2, comparison between IMD treatment and placebo.
	Assessment, including difference between IMD treatment and placebo in changes from V2 (baseline) to V3 (week 6) and to V4 (week 12), of:<br>- systolic/diastolic blood pressure<br>- and pulse rate.
	Assessment, including difference between IMD treatment and placebo in changes from V2 (baseline) to V4 (week 12), of:<br>- safety laboratory parameters (blood count, liver, and renal function parameters).
	Assessment of DDs throughout the study treatment period.
	Assessment, including difference between IMD treatment and placebo in changes from V2 (baseline) to V4 (week 12) of:<br>- vitamin B12 (cobalamin) and vitamin D<br>- trace elements Mg and Zn.

8.4 Other Explorative Parameters

Explorative objectives	Explorative endpoints
To evaluate the effect of the IMD treatment on HbA1c levels, body weight assessed on site, HOMA-IR, lipid levels, FBI, FBG levels, SAD, waist-hip-ratio, body composition in comparison to placebo.	Difference between IMD treatment group and placebo group in changes from V2 (baseline) to V3 (week 6) in:<br>- HbA1c levels<br>- Body weight assessed on site<br>- HOMA-IR<br>- TC<br>- FBI<br>- FBG<br>- LDL-C<br>- SAD<br>- TG<br>- Waist-hip-ratio<br>- Body fat content/mass, fat free mass (assessed by BIA)
To evaluate the effect of the IMD treatment on Homeostasis Model Assessment for Beta Cell Function (HOMA-B), Quantitative insulin sensitivity check index (QUICKI), lipid levels, cardiometabolic risk indices, waist (WC) and hip circumference (HC) in comparison to placebo.	Difference between IMD treatment group and placebo group in changes from V2 (baseline) to V3 (week 6) and to V4 (week 12) in:<br>- HOMA-B, QUICKI<br>- VLDL-C, HDL-C, non-HDL-C<br>- Atherogenic index of plasma (AIP), atherogenic coefficient (AC) and cardiac risk ratio (CRR) 1 and 2<br>- WC and HC<br>- BMI
To evaluate the effect of the IMD treatment on hs-CRP in comparison to placebo.	Difference between IMD treatment group and placebo group in changes from V2 (baseline) to V4 (week 12) in hs-CRP levels.
To assess subject satisfaction and the ease of use of study treatment.	Assessment of subject satisfaction and ease of use of study treatment at V4 (week 12).
To evaluate the microbiome diversity in stool samples.	Explore the differences in intestinal flora composition at V2 (baseline) and V4 (week 12) and content between IMD treatment group and placebo group.

9 INVESTIGATIONAL PLAN

9.1 Overall Investigational Design and Schedule of Events

The present clinical investigation is a randomized, double-blinded, placebo-controlled, multicenter international study, planned to be conducted in Poland, Romania and Slovakia. The investigation will be performed to establish clinical evidence on the performance and safety of the IMD and is aiming at evaluating the suitability of the product for the intended purpose and population.

Based on the intended claim for the IMD, the primary endpoint regarding clinical performance is focusing on the effects on HbA1c (see section 8.2). HbA1c is considered as the principal parameter in the state-of-the-art management of blood glucose control. The change in HbA1c is generally considered as an adequate main study endpoint with respect to claims on blood glucose reduction/improvement of impaired blood glucose control (e.g., European Food Safety Authority [EFSA], 2012; EMA, 2018; Food and Drug Administration [FDA], 2022). The time period of the assessment phase is based on the recommendations by EMA (2018), considered in this context as medically relevant also for the present IMD clinical investigation.

Use of HbA1c in prediabetes/T2D assessment requires implementation of internationally standardized reference measurement methods adhering to high quality assurance procedures (WHO, 2011; Little et al., 2019; ADA, 2022). The HbA1c assessment in the present study will be performed in a central laboratory applying a certified and standardized method, also allowing for standardization across the multiple sites/countries.

In a large-scale clinical study in prediabetes subjects by the Diabetes Prevention Program (DPP) Research Group (Maruthur et al., 2013), a decrease in HbA1c levels by 0.6 mmol/mol (mean difference) was observed at 6 months of metformin treatment. The continued metformin treatment during the study period of 3 years was reported to reduce the incidence of T2D by 31% compared to placebo (Knowler et al., 2002); after the washout phase to eliminate the pharmacological effect of metformin while taking the drug, the reduction of incidence amounted to 25% (DPP Research Group, 2003).

Potential confounding factors, such as relevant concurrent disorders/treatments, have been taken into account in the eligibility criteria and will additionally be monitored throughout the study. To further minimize bias, the randomization will be performed according to the most relevant stratification factors, HbA1c level (<48/≥48 mmol/mol; <6.5%/≥6.5%), BMI (<30/≥ 30 kg/m^2), and site.

The main secondary performance objective is to evaluate the effect of IMD on body weight, measured on site. The motivation for this assessment is that on one hand, reduced calorie utilization may be expected by use of the IMD and on the other, excess body weight is considered as one of the major risk factors for diabetes (Kivimäki et al., 2017). Complementary data on relevant traits of metabolic syndrome (blood lipids, WC, SAD) and further significant markers such as body composition will be collected as well. Finally, a

DocuSign Envelope ID: DC97F66E-B279-4A14-AC63-5C291FAB6C7D

possible impact of the study treatment on the quality of life will be rated. The safety objective is to assess the clinical safety of the IMD, including evaluation of SADE, ADE, AE and DDs. In addition, systolic/diastolic blood pressure and pulse rate will be measured, and laboratory parameters will be assessed to examine any clinically relevant changes upon IMD use on blood status, renal and liver parameters, as well as levels of minerals and vitamins (see section 8.3).

Visits and assessments will be performed as described in Table 9.1. Details of assessment methods are provided in sections 9.6.3 and 9.6.4; the analysis procedures are described in section 9.9.

Table 9.1 Schedule of events

**Procedure/ Assessment**	**Visit 1 Screening**	**Visit 2 Baseline Start of Treatment**	**Phone call 1**	**Visit 3 Control**	**Phone call 2**	**Visit 4 Final visit End of treatment**	**Phone call 3 (follow-up)**	**Phone call 4 (follow-up)**
	Day -14 to Day -5	Day 0	Day 21 ±3 (3 weeks ±3 days after V2)	Day 42 ±3 (6 weeks ±3 days after V2)	Day 63 ±5 (9 weeks ±5 days after V2)	Day 84 ±5 (12 weeks ±5 days after V2)	7-10 days after V4	21-35 days after V4
Subject information	X							
Written informed consent, consent for data processing	X							
Inclusion and exclusion criteria	X							
Confirmation of eligibility criteria[f], randomization		X						
Anamnestic, demographic data	X							
Questioning on smoking status	X	X		X		X		
Medical history/concurrent diseases	X							
Concurrent treatment	X	X	X	X	X	X	X	X
Urine pregnancy test for women of childbearing potential	X							
Physical examination	X					X		
12 lead ECG	X							
Blood pressure, pulse rate	X	X		X		X		
Body weight on site	X	X		X		X		
Body height and BMI[e]	X							

**Procedure/ Assessment**	**Visit 1 Screening**	**Visit 2 Baseline Start of Treatment**	**Phone call 1**	**Visit 3 Control**	**Phone call 2**	**Visit 4 Final visit End of treatment**	**Phone call 3 (follow-up)**	**Phone call 4 (follow-up)**
	Day -14 to Day -5	Day 0	Day 21 ±3 (3 weeks ±3 days after V2)	Day 42 ±3 (6 weeks ±3 days after V2)	Day 63 ±5 (9 weeks ±5 days after V2)	Day 84 ±5 (12 weeks ±5 days after V2)	7-10 days after V4	21-35 days after V4
Waist and hip circumference, SAD		X		X		X		
Body composition assessment (BIA)		X		X		X		
Fasted blood draw for HbA1c, glucose, insulin, and lipid parameters [a] (central lab)	X	X		X		X		
Fasted blood draw for safety parameters (blood count [b], liver [c] and renal [d] parameters) (central lab)	X	X				X		
Fasted blood draw for TSH (central lab)	X							
Fasted blood draw for hs-CRP (central lab)	X	X				X		
Blood draw for analyses of vitamins/trace elements[g] (central lab)		X				X		
12 Items Short Form Health Survey (SF-12)		X				X		
Issue of study treatment and instructions on study treatment use		X		X				
Issue of subject log and scales to subject, instruction		X						
Check of subject log (weight)			X			X	X	
Issue of material and instructions for stool sample collection	X			X				

**Procedure/ Assessment**	**Visit 1 Screening**	**Visit 2 Baseline Start of Treatment**	**Phone call 1**	**Visit 3 Control**	**Phone call 2**	**Visit 4 Final visit End of treatment**	**Phone call 3 (follow-up)**	**Phone call 4 (follow-up)**
	Day -14 to Day -5	Day 0	Day 21 ±3 (3 weeks ±3 days after V2)	Day 42 ±3 (6 weeks ±3 days after V2)	Day 63 ±5 (9 weeks ±5 days after V2)	Day 84 ±5 (12 weeks ±5 days after V2)	7-10 days after V4	21-35 days after V4
Stool sample collection[h] (central lab)		X				X		
Study treatment collection and compliance check[i]				X		X		
Adverse events	X	X	X	X	X	X	X	X
Device deficiencies			X	X	X	X		
Body weight measurement off site[j]		X				X	X	
Subject satisfaction and ease of use of study treatment[k]						X		

[a] Lipid parameters: TG, TC, LDL-C, HDL-C. VLDL-C and non-HDL-C will be calculated within the eCRF.

[b] Blood count: hemoglobin, hematocrit, erythrocytes, thrombocytes, reticulocytes, leucocytes

[c] Liver function parameters: ALAT, ASAT, Gamma-GT, alkaline phosphatase, bilirubin

[d] Renal function parameters: creatinine, urea, cystatine C, eGRF, uric acid

[e] BMI will be assessed on site at V1 and during the statistical analysis for all later time points, calculated within the eCRF based on body weight measured on site

[f] With respect to laboratory findings, only the blood draw at V1 is relevant for the enrolment/randomization

[g] Vitamins/trace elements: vitamin B12, vitamin D, Mg, Zn

[h] Two samples each time in the week before the visit; for gut microbiome parameters and storage for further putative later microbiome analyses

[I] Number of unused stick packs returned to be entered in the eCRF. Compliance will be calculated during the statistical analysis.

[j] One day after V2 and thereafter on the day of V4/PC3.

[k] Questions to be asked by site personnel and answers to be captured in the eCRF.

9.2 Duration of the Study

The study duration for each subject is approximately 16-18 weeks, with 12 weeks of study treatment.

The expected start date of this study is Q3 2023. The overall duration of the study (from first subject first visit to last subject last visit) is expected to be approximately 13 months.

9.3 Justification for the Design of the Clinical Investigation

The standard of care in the management of prediabetes consists of lifestyle modifications - dietary changes and increased physical activity. However, research shows making long-lasting improvements in diet and lifestyle are challenging for most individuals (Echouffo-Tcheugui et al., 2021). Prediabetes will progress to overt T2D in approximately 25% of subjects within 3–5 years, and as many as 70-90% of individuals with prediabetes will develop overt diabetes within their lifetime (Tabák et al., 2012).

The American Diabetes Association emphasizes that where lifestyle modifications are ineffective (which they are in majority of cases), pharmacotherapy in the form of metformin should be given especially to high-risk prediabetic individuals with an HbA1c level of 6% (equivalent to 42 mmol/mol) (Nuha et al., 2023). There are treatments available for blood sugar reduction in prediabetics and early Type 2 diabetics, such as Metformin and other agents which reduce blood sugar levels.

However, the first-line pharmacological treatment for T2D, metformin, is associated with side effects, predominantly in the GI tract (e.g., nausea, vomiting, diarrhea). In this context, the IMD SiPore21® has been developed to provide a novel and well-tolerated solution to improve blood glucose control. The decision to include drug-naïve T2D, was a recommendation from Key Opinion Leaders, as this patient population (HbA1c levels within the range of 48-58 mmol/mol) in practice oftentimes is recommended to change their lifestyle within 6 months before being put on pharmacotherapy. It is recognized that at an early stage of diabetes onset, with appropriate lifestyle measures and tools, diabetes can be reversed to normal state.

The clinical development stage of the IMD is at the pre-market level (before market approval). The present pivotal, confirmatory, interventional clinical investigation will be conducted with the aim to demonstrate conformity of the IMD as defined in the requirements stated in the MDR.

In line with the current Clinical Development Plan (CDP) and Clinical Evaluation Plan (CEP) for the IMD (both on file with the Sponsor), the foreseen assessment methods and the outcome parameters to be explored in the study have been selected to generate clinical evidence of conformity with regard to general safety and performance requirements and enable conclusive findings with respect to the clinical benefit of the IMD.

9.4 Selection of Investigational Population

9.4.1 Number of Subjects

288 subjects will be enrolled according to inclusion and exclusion criteria at V1.

As the IMD is intended for obese or overweight adult subjects with impaired blood glucose control, the investigation population will be selected based on the respective inclusion criteria: HbA1c level ≥42 to ≤58 mmol/mol (≥6 to ≤7.5%) and BMI >25 kg/m^2 to ≤40 kg/m^2.

Individuals with HbA1c levels >58 mmol/mol (>7.5%) and/or BMI >40 kg/m^2 will not be included, due to an increased need for treatment options to control blood glucose and body weight, which are not allowed in the present study to minimize the bias with regard to the primary outcome.

Furthermore, individuals with any severe concurrent diseases which may interfere with the adherence to the study procedures as per investigator's judgement will not be included in the study for reasons of study compliance.

Since the main site of action for the IMD is the GI tract, individuals with relevant diseases of the GI tract will not be included.

9.4.2 Screening and Enrolment Log

Each clinic will keep a log in the ISF of all subjects screened and included. The reason for screen failure should be stated for all subjects screened but not included. The reason for withdrawal should be stated for all subjects included but not completed.

9.4.3 Inclusion Criteria

For inclusion in the investigation, subjects must fulfil all the following criteria:

1. Male or female 18-70 years old
2. HbA1c level ≥42 to ≤58 mmol/mol (≥6 to ≤7.5% - according to Diabetes Control and Complications Trial (DCCT, 1987) at V1

   For Poland only: HbA1c level ≥42 to □53 mmol/mol (≥6 to ≤7% - according to the Official Journal of the Diabetes Poland, 2023 Vol. 3 Issue 1) at V1
3. Body mass index (BMI) >25 kg/m2 and ≤40 kg/m2
4. Regular intake of 3 main meals (self-reported)
5. Readiness and ability to:
   a. use the study treatment as recommended and attend all scheduled visits
   b. comply with all further study procedures
6. Readiness to maintain the current diet and level of physical activity during the study

7. Readiness not to participate in another clinical study during this study
8. Women of childbearing potential: commitment to use medically recognized contraception methods during the treatment period
9. Written informed consent by the participant following written and oral information by the investigator regarding nature, purpose, consequences and possible risks of the clinical study

9.4.4 Exclusion Criteria

Subjects must not enter the investigation if any of the following exclusion criteria are fulfilled:

1. Known allergy or hypersensitivity to the components of the IMD or placebo (self-reported)
2. Type 1 diabetes (T1D)/Latent Autoimmune Diabetes in Adult or secondary diabetes (self-reported)
3. Uncontrolled hypertension (regularly >179/109 mmHg [self-reported] and as per investigator's judgement based on screening procedures at V1)
4. History (self-reported) of myocardial infarction or stroke 6 months prior to V1
5. Clinically relevant abnormal electrocardiogram (ECG) at V1
6. History (<3 years prior to V1) or presence (self-reported) of:
    a. exocrine pancreatic insufficiency, chronic pancreatitis
    b. chronic inflammatory bowel disease, celiac disease
    c. diverticulosis (usually affecting the large intestine), adhesions, chronic constipation
7. State after pancreatic head resection with the need for additional intake of pancreatic enzymes (self-reported)
8. Major surgery of esophagus, stomach, intestine including colon which took place <3 years prior to V1, or >3 years prior to V1 in case of related current clinical symptoms (self-reported)
9. Clinically significant deviation, based on investigators judgment, in blood laboratory values at V1 of blood status (hemoglobin, erythrocytes, platelets, leucocytes, reticulocytes), kidney parameters (creatinine, cystatin C and estimated glomerular filtration rate), thyroid hormone status: TSH
10. Deviation in blood laboratory values at V1 of liver parameters (ASAT, ALAT), alkaline phosphatase and Gamma-GT) that is clinically significant based on investigators judgment

11. Blood donation/other major blood loss or blood transfusion, that may interfere with the study as per investigator's judgment, within 56 days prior to V1 and any blood donation or transfusion during the study
12. Previous or current metformin or other medical anti-diabetic treatment or blood glucose levels reducing/influencing treatment/supplementation within 30 days prior to V1 and during the study
13. Current treatment/supplementation for weight management (e.g., fat binder/burner, carb blocker, satiety products) or known to influence weight (e.g., systemic corticosteroids)
14. Medical conditions that require medications taken during meals
15. Extreme diet form (e.g., ketogenic, very low carbohydrate) during the last 3 months prior to study
16. Self-reported regular average consumption of >1 L/day total of sugary beverages (e.g., soft drinks, fruit juices, energy drinks) and/or >200 g/day total of food based on simple sugar(s) between meals (e.g., commercial candies, dried fruit)
17. Pregnancy, lactation or active planning to achieve pregnancy
18. History of or current abuse of drugs, alcohol or medication
19. Any severe diseases/disorder (e.g., chronic kidney disease, neoplastic disease or psychiatric disorder) which may interfere with the compliance to the study procedures as per investigator’s judgement
20. Participation in another study during the last 30 days prior to V1
21. Belonging to a vulnerable population, having any condition or other reason which in the opinion of the investigator would confound the conduct of the study or interpretation of the study results
22. Relative of the investigator or an employee at the clinical study site and Sponsor

9.4.5 Women of Childbearing Potential

Female subjects may not be pregnant at the time of inclusion into the study. Therefore, pregnancy testing will be performed during screening for women with childbearing potential (women of non-childbearing potential are defined as those who either have no uterus, or have undergone ligation of the fallopian tubes, or have permanent cessation of ovarian function due to ovarian failure or surgical removal of the ovaries, or are in the post menopause).

During the informed consent process, the investigator will advise every woman with childbearing potential that she must use medically recognized contraception methods during the study period. Subjects will be instructed not to take the IMD for at least 2 hours before or after taking any oral contraceptives.

DocuSign Envelope ID: DC97F66E-B279-4A14-AC63-5C291FAB6C7D

In case pregnancy occurs, the subject must be withdrawn from treatment and the subject discontinued from participation in the investigation. Any pregnancy during the study must be reported (see Section 9.7.8).

9.4.6 Restrictions

The subjects are to report on the use of any concomitant treatment (e.g., other medical devices, medication, any natural health products including food supplements etc.) to the investigator at the first visit. The investigator is to document the following information in the eCRF: substance, dosage, start and duration, reason. Subjects will also be advised to inform the investigator of any additional treatment performed throughout the clinical investigation period.

During the study, the following concomitant treatment listed in the exclusion criteria should not be used:

- medications taken during meals
- metformin or other medical anti-diabetic treatment or blood glucose levels reducing/influencing treatment/supplementation*

At enrollment, the investigator informs the subjects that any concomitant treatment that could possibly influence the outcome of the study as specified above is not allowed. If a subject will suffer any medical condition during the study that require medications taken during meals will be excluded from the study (see Section 12.2.1).

As per the IFU, the subjects will be instructed not to take the IMD at least 2 hours before or after taking any concurrent medication.

*if at any time during a subject's participation in the study the investigator determines that the HbA1c levels of the subject are not improving or worsening, or if clinically indicated, the subject will be immediately withdrawn from the study, IMD treatment will be stopped, and standard of care metformin or other medical anti-diabetic treatment will be initiated.

9.5 Identification and Description of the Investigational Medical Device and Placebo

9.5.1 Description of the IMD and Placebo

The IMD, SiPore21®, is an oral gel, containing silica particles with customized small pores. The silica particles (principal component), named MSP21, are micron sized and the diameter of their pores is around 10 nm. The pores of the silica physically entrap a portion of the digestive enzymes from the intestinal fluid. This physical separation reduces the breakdown and uptake of carbohydrates and fats from food, leading to a reduction in total energy intake. This results in improved blood glucose control, as measured by a reduction in long-term blood glucose, HbA1c level.

MSP21 is a type of synthetic amorphous silica (SAS). SAS is non-toxic, FDA-approved (FDA, 21CFR172.480), GRAS-listed (generally recognized as safe [FDA, 21CFR182.90]) and is widely used in foods, cosmetics, and pharmaceutical formulations as an excipient. SAS is inert when ingested orally. Silica, also known as silicon dioxide, is approved as a food additive in Europe under the food additive number E551. MSP21 specifications fulfill E551 purity requirements and the manufacture of MSP21 meets E551 production requirements (See *GR 2022-06-08 MSP21 and E551 specifications* and *GR 2022-10-10 MSP21 meets E551 manufacturing process requirements*). Absorption of orally ingested silica is negligible, silica is not metabolized, and it is excreted in the feces (ATSDR, 2019). Therefore, it can be regarded that MSP21 absorption is negligible, and it is not metabolized by the body. Instead, it acts locally in the GI tract.

The IMD is a surface medical device that comes in contact with intact mucosal membranes for > 30 days.

The IMD is packed in stick packs, each containing 3 g silicon dioxide. The contents of a single stick pack should be taken orally, 3 times daily, each in connection with the day's main meals, giving a total daily oral dose of 9 g silicon dioxide. The IMD will be taken for a maximum of 12 weeks.

The IMD is targeted to be classified as a Class IIb medical device according to the classification rules of the European Medical Device Regulation (EU) 2017/745 (rules 5 and 21). Refer to Table 9.2 for the composition of the IMD.

Table 9.2 Composition of the IMD

Component	Food additive number	CAS number	g/dose	wt%
Water	n.a	7732-18-5	27.300	85.634
MSP21 (component responsible for achieving the intended purpose)	E551	7631-86-9	3.000	9.410
Erythritol	E968	149-32-6	1.197	3.755
Xantham gum	E415	11138-66-2	0.200	0.627
Citric acid	E330	77-92-9	0.070	0.220
Potassium sorbate	E202	24634-61-5	0.030	0.094
Vanilla extract	n.a.	n.a.	0.050	0.157
Blueberry flavor	n.a.	n.a.	0.020	0.063
Peach flavor	n.a.	n.a.	0.010	0.031

**Component**	**Food additive number**	**CAS number**	**g/dose**	**wt%**
Steviol glycosides	E960	n.a.	0.003	0.009
Total			*31.880*	*100.000*

The placebo is comparable to the IMD in appearance, texture, taste, and smell. The placebo contains maltodextrin in place of the principal component of the IMD. Refer to Table 9.3 for the composition of the placebo.

Table 9.3 Composition of the placebo

**Component**	**Food additive number**	**CAS number**	**g/dose**	**wt%**
Water	n.a	7732-18-5	27.296	85.621
Maltodextrin	E1400	9050-36-6	3.046	9.556
Erythritol	E968	149-32-6	1.252	3.927
Gellan gum	E418	71010-52-1	0.063	0.197
Citric acid	E330	77-92-9	0.105	0.328
Potassium sorbate	E202	24634-61-5	0.031	0.098
Vanilla extract	n.a.	n.a.	0.052	0.164
Blueberry flavor	n.a.	n.a.	0.021	0.066
Peach flavor	n.a.	n.a.	0.011	0.033
Steviol glycosides	E960	n.a.	0.003	0.010
Total			*31.880*	*100.000*

For further information, please refer to the latest version of the IB.

9.5.2 Manufacturer of the IMD and Placebo

Sigrid Therapeutics AB

C/O 7A Odenplan

Norrtullsgatan 6, plan 5

DocuSign Envelope ID: DC97F66E-B279-4A14-AC63-5C291FAB6C7D

SE-113 29 Stockholm, Sweden

9.5.3 Production, Packaging and Labelling of IMD and Placebo

Production, packaging and labelling of the IMD and placebo is the responsibility of the Sponsor.

The manufacturing process of MSP21 is FSSC 22000 certified. The manufacturing process follows the process described by the European Food Safety Agency for E551, silica gel.

The manufacturing process of SiPore21® and placebo is certified according to food safety standards BRCGS and NSF/ANSI 173 section 8, which includes cGMP.

The study treatment will be packed in a home box. Each home box will contain 7 weekly boxes and each weekly box will contain 21 single dose stick packs.

The labelling of the tertiary packs (home box) and secondary packs (weekly box) follow EN ISO 15223-1.

9.5.4 Storage, Inventory, Return, Traceability and Documentation of the IMD and Placebo

The IMD and the placebo will be provided by the Sponsor. The investigational sites will be responsible for storing the study treatment in a proper place, at the appropriate conditions and inaccessible to unauthorized persons. The investigator (or designee) will dispense study treatment to subjects.

The study treatment should be stored at room temperature. Additionally, it should be kept away from sunlight and shall not be frozen. Once opened, the stick pack is to be consumed immediately.

Unused (if any) stick packs and boxes will be collected from the subjects at the sites and the study treatment accountability will be performed. After completion of the investigation, the unused study treatment will be returned to the Sponsor.

The Sponsor and site personnel will keep records documenting the location of the IMD and the comparator from shipment from Sponsor, distribution for usage to study participants, to return to Sponsor. Shipment documentation will be kept at the Sponsor and device accountability will be documented at the sites (see Section 10).

9.5.5 Intended Purpose of the IMD in the Proposed Clinical Investigation

The purpose of the IMD is to reduce blood glucose levels (as measured by HbA1c) and improve blood sugar control as compared to placebo.

The IMD is intended for self-treatment.

9.5.6 Populations and Indications for which the IMD is Intended

The IMD is intended for overweight or obese (BMI ≥25 kg/m^2) prediabetic individuals with HbA1c levels of 42-47 mmol/mol or T2D patients with HbA1c levels of 48-58 mmol/mol.

9.5.7 Required Training and Experience of Users

On V2, the subject will be instructed on the use of the study treatment and provided IFU.

During the treatment period of 12 weeks, the IMD/placebo should be taken 3 times a day. It should be taken with the 3 largest meals of the day (e.g., breakfast, lunch, dinner). It should be taken in direct connection with the first bite of each respective meal, preferably after having chewed (if solid food) and swallowed the first bite of the meal.

Subjects are instructed to drink one glass of water (at least 200 mL) with each main meal when the IMD is taken.

In case the study treatment is accidentally not taken:

- at the start of a meal, it is to be taken anytime during the meal
- in conjunction with a meal, it is to be taken as soon as possible after the meal

For further information, please refer to the IFU.

9.5.8 Medical or Surgical Procedures Involved in the Use of the Device

N/A. There are no medical or surgical procedures involved in the use of the IMD or placebo.

9.5.9 Method of Assigning Subjects to Treatment Groups and Blinding

The clinical investigation will be conducted in a randomized double-blinded manner. Stratified randomization will be performed according to the following criteria at V1: HbA1c level (<48/≥48 mmol/mol), BMI (<30/≥ 30 kg/m^2) and site.

The randomization list will be provided to the Sponsor's assigned responsible person (not involved in the study) by the statistician responsible for generation of the randomization list. The ratio of randomization between the IMD and placebo groups will be 1:1.

Based on the randomization list, allocation of the study treatment to the subjects will be done by a web-based randomization system (IWRS).

Subjects who withdraw from the study will not be replaced.

Randomization list will be concealed to the investigational sites. It will be stored under lock and key by the Sponsor until database closure.

After database closure and sign-off of the SAP, the Sponsor's responsible person will provide the randomization list to the project manager at the CRO and the statistician responsible for the statistical analysis.

9.5.10 Blinding and Emergency Decoding of Blinded Treatment

The IMD and placebo are comparable in appearance, texture, taste, and smell, and indistinguishable in packaging and labelling, so that study participants, CRO and investigators are blinded to treatment assignment.

Emergency unblinding will be implemented in the randomization system and is strictly restricted to authorized persons at participating study sites. In case of emergency unblinding the study management personnel will be automatically notified by the system.

Emergency unblinding should be performed by the investigator only in emergency cases (e.g., SAE), in which the investigator suspects a causal relation with the study treatment, requiring unblinding for appropriate treatment of the subject. The CRO will be notified by the randomization system and will promptly inform the Sponsor. Emergency unblinding will be documented in the randomization system including the randomization number of the study participant to be unblinded, dispensed package numbers to the study participant, performing person, specific reason for and date and time of unblinding. Emergency unblinding will also be documented in the eCRF by documenting the performing person, the specific reason for and the date and time of unblinding.

Any cases of emergency unblinding will be checked during monitoring.

9.6 Investigational Assessments

The investigational assessments are described in the sections below and the timing of these assessments is detailed in the schedule of events (Table 9.1, Section 9).

9.6.1 Visit Schedule

9.6.1.1 Visit 1 (V1, screening), Day -14 to Day -5

- Oral and written information about the nature, purpose, possible risks and benefits of the study provided to the subjects by the investigator
- Written consent of the subject to participate, and consent for data processing; the subject understands the requirements of the clinical investigation and is willing to comply
- Questioning and recording of the medical history, concurrent diseases and concomitant treatment, smoking status and demographic data
- Questioning and recording of possible occurrence of AEs
- Pregnancy test (urine) for women of childbearing potential
- Physical examination
- 12 lead ECG

- Assessment of body weight (fasted condition), height and BMI
- Measurement of blood pressure and pulse rate
- Verification that the inclusion criteria are met and that there are no violations of the exclusion criteria
- Blood draw (fasted condition) for:
  - safety laboratory parameters (blood count, liver and renal function parameters)
  - TSH
  - hs-CRP
  - HbA1c, fasting glucose, fasting insulin
  - lipid parameters
- Issue of material and instructions for stool sample collection

9.6.1.2 Visit 2 (baseline/randomization), Day 0

- Confirmation of eligibility criteria (with respect to laboratory findings, only the blood draw at V1 is relevant for randomization; concerning stool sample, any subjects not providing the sample would still be eligible)
- Randomization
- Questioning and recording of possible occurrence of AEs
- Questioning and recording of new or changed concurrent treatment and smoking status
- Collection of stool sample
- Assessment of body weight on site
- Body composition assessment (BIA) – body fat content/mass, fat free mass
- Measurement of blood pressure and pulse rate
- Blood draw (fasted condition) for:
  - safety laboratory parameters (blood count, liver and renal function parameters)
  - hs-CRP
  - HbA1c, fasting glucose, fasting insulin
  - lipid parameters
  - analyses of vitamins/trace elements

- Measurement of WC and HC
- Measurement of SAD
- SF-12
- Issue of study treatment and instruction for use
- Handing out the subject log, scale and instructions for determining body weight off site on the morning of the next day after V2, before the intake of the first meal and the first intake of study treatment

9.6.1.3 Phone Call 1, Day 21 ±3 (3 weeks ±3 days after V2)

- Questioning and documentation of possible occurrence of AEs/DDs
- Questioning and documentation of new or changed concurrent treatment
- Control of subject log

9.6.1.4 Visit 3 (control), Day 42 ±3 (6 weeks ±3 days after V2)

- Questioning and recording of possible occurrence of AEs/DDs
- Questioning and recording of new or changed concurrent treatment and smoking status
- Assessment of body weight on site
- Body composition assessment (BIA) – body fat content/mass, fat free mass
- Measurement of blood pressure and pulse rate
- Blood draw (fasted condition) for:
    - HbA1c, fasting glucose, fasting insulin
    - lipid parameters
- Measurement of WC and HC
- Measurement of SAD
- Return of study treatment and intermediary compliance check
- Issue of study treatment and instructions
- Issue of material for stool sample collection

9.6.1.5 Phone Call 2, Day 63 ±5 (9 weeks ±5 days after V2)

- Questioning and documentation of possible occurrence of AEs/DDs
- Questioning and documentation of new or changed concurrent treatment

9.6.1.6 Visit 4 (final visit), Day 84 ±5 (12 weeks ±5 days after V2)

- Measurement of body weight off site prior to V4, in the morning of the day of V4, before intake of first meal
- Questioning and recording of possible occurrence of AEs/DDs
- Questioning and recording of new or changed concurrent treatment and smoking status
- Control of subject log
- Stool sample collection
- Assessment of body weight on site
- Body composition assessment (BIA) – body fat content/mass, fat free mass
- Measurement of blood pressure and pulse rate
- Blood draw for:
    - safety laboratory parameters (blood count, liver and renal function parameters)
    - hs-CRP
    - HbA1c, fasting glucose, fasting insulin
    - lipid parameters
    - analyses of vitamins / trace elements
- Measurement of WC and HC
- Measurement of SAD
- SF-12
- Return of study treatment and documentation of compliance
- Subject satisfaction and ease of treatment use
- Physical examination

9.6.1.7 Phone Call 3, 7-10 days after V4

- Determination of body weight off site prior to PC3, in the morning of the day of PC3, before the intake of the first meal (the value of body weight should be questioned by the site and documented in the eCRF)
- Questioning and documentation of possible occurrence of AEs
- Questioning and documentation of new or changed concurrent treatment
- Control of subject log

9.6.1.8 Phone Call 4, 21-35 days after V4

- Questioning and documentation of possible occurrence of AEs
- Questioning and documentation of new or changed concurrent treatment

9.6.2 Screening Procedures

In case a subject does not meet the inclusion criterion with respect to the HbA1c, re-screening is possible after >4 weeks if the value at first screening is less than 5% different than the specified range.

If any pathology is detected, the investigator is responsible for assessing if the subject needs additional medical attention e.g., referral to general practitioner as applicable.

9.6.2.1 Demographic, Anthropometric and Anamnestic Data at Screening

The subject's age (years), gender (male/female), ethnicity (Caucasian, Asian, or other), body height (cm), body weight (kg) and BMI ((kg)/(height [m])^2) will be assessed and documented in the eCRF.

Body weight (kg) is measured in subjects wearing only underwear and barefoot, after emptying the bladder and bowels as needed, using calibrated weighing scales (Tanita MC-780S MA). Body height will be measured in barefoot subjects using standard devices (WHO, 1995).

The subject will be questioned by the investigator about anamnestic data (including medical history and concurrent diseases and treatment), smoking status and will undergo a physical examination including in sitting blood pressure and pulse rate accordance with standard clinical practice. Known pre- or co-morbidities as well as any abnormal and/or pathological findings of the examination will be systematically recorded.

Further, a 12 lead ECG will be performed using standard devices and procedures.

9.6.2.2 Laboratory Parameters at Screening

Venous blood samples will be drawn after fasting overnight for analysis of:

- blood status (hemoglobin, hematocrit, erythrocytes, thrombocytes, leucocytes, reticulocytes), liver (ALAT, ASAT, Gamma-GT, alkaline phosphatase, bilirubin), and renal function parameters (creatinine, urea, cystatin C, estimated glomerular filtration rate (eGRF), uric acid),
- HbA1c, FBG, FBI, TSH, hs-CRP, and the lipid parameters (TG, TC, LDL-C, HDL-C).

The measurements will be performed in a central laboratory according to standard procedures. Details on collection, handling, storage and transport of samples to central lab will be provided in laboratory instructions prior to study start and filed in the ISF and Trial

Master File (TMF). A urine pregnancy test will be performed in women with childbearing potential.

9.6.3 Assessment of Efficacy/Performance

9.6.3.1 Blood Glucose Control Parameters

Venous blood samples will be collected after overnight fasting to determine HbA1c, FBG, and FBI. The measurements will be performed in a central laboratory according to standard procedures. Details on collection, handling, storage and transport of samples will be provided in laboratory instructions prior to study start and filed in the ISF and TMF.

Homeostasis Model Assessment (HOMA) indices will be calculated during the statistical analysis as follows (Matthews et al., 1985):

- HOMA for Insulin Resistance (IR) (mmol/l) = FBI (mU/l) * FBG (mmol/l) / 22,5
- HOMA for Beta Cell Function (B) = 20 * FBI (mU/l) / (FBG (mmol/l) – 3,5).

Quantitative insulin sensitivity check index (QUICKI) will be calculated during the statistical analysis as follows (Katz et al., 2000):

- QUICKI = 1/[log(FBI)) + log(FBG)]

9.6.3.2 Lipid Levels

Venous blood samples will be collected after overnight fasting to determine TG, TC, HDL-C, LDL-C. The measurements will be performed in a central laboratory according to standard procedures. Details on collection, handling, storage and transport of samples will be provided in laboratory instructions prior to study start and filed in the ISF and TMF.

Non-HDL-C and VLDL-C will be calculated during the statistical analysis as follows:

- VLDL-C = TG / 5
- Non-HDL = TC - HDL

9.6.3.3 Atherogenic Index, Atherogenic Coefficient, Cardiac Risk Ratio

The cardiometabolic risk indices will be calculated during the statistical analysis as follows:

- Atherogenic Index of Plasma (AIP): log10 (TG/HDL-C)
- Atherogenic Coefficient (AC): [(TC- HDL-C)/HDL-C]
- Cardiac risk ratio 1 (i.e., Castelli's Risk Index (CRI) I: TC/HDL-C
- Cardiac risk ratio 2 (i.e., CRI II): LDL-C/HDL-C

9.6.3.4 Body Weight Measurement – On Site

Body weight (kg) will be measured on site in subjects in fasted condition, wearing only underwear and barefoot, after emptying the bladder and bowels as needed, using calibrated weighing scales (Tanita MC-780 S MA).

9.6.3.5 Body Weight Measurement – Off Site

Body weight (kg) off site will be measured by subjects themselves, in fasted condition, preferably in the morning, wearing only underwear and barefoot, after emptying the bladder and bowels as needed, using standardized scales to be provided at V2, along with the instructions for measurement. The subjects will record the body weight in a paper subject log. Documentation on the implemented scales will be filed in the ISF and TMF.

9.6.3.6 Body Composition

Body fat mass (kg) and fat free mass (kg) will be measured by the BIA method using validated and calibrated electronic weighing scales (Tanita MC-780 S MA) according to the instruction manual.

9.6.3.7 Waist/Hip Circumference

Waist circumference (WC) (cm) will be measured at the level midway between the lateral lower rib margin and the iliac crest; hip circumference (HC) (cm) is measured as the maximal circumference over the buttocks (WHO, 1995).

9.6.3.8 Sagittal Abdominal Diameter

Sagittal abdominal diameter (SAD) (cm) will be measured by means of a standardized abdominal caliper as described by Sampaio et al. (2007).

9.6.3.9 Short Form Health Survey

General well-being will be assessed by the self-reported SF-12 (Appendix 14.3) containing 12 items clustered into eight dimensions: physical functioning, role limitations due to physical health problems, bodily pain, general health, vitality (energy/ fatigue), social functioning, role limitations due to emotional problems and mental health (psychological distress and psychological wellbeing).

9.6.4 Assessment of Safety

9.6.4.1 Adverse Events and Device Deficiencies

Any AE or DD that occurs during the course of the clinical investigation (for evaluation and reporting see section 9.7), will be recorded in the eCRF.

After signing of informed consent, the subjects will be provided with emergency contact

details so that they can report events/observations that may qualify as serious AEs or SADEs without delay.

9.6.4.2 Blood Pressure/Pulse Rate

Sitting blood pressure and pulse rate will be measured upon at least 10 minutes rest, using standard devices and procedures.

9.6.4.3 Central Laboratory Parameters

9.6.4.3.1 Safety Parameters

Venous blood samples will be drawn after fasting overnight for analysis of routine safety parameters:

- full blood count parameters (hemoglobin, hematocrit, erythrocytes, thrombocytes, reticulocytes, and leucocytes)
- liver function parameters (ALAT, ASAT, gamma-GT, alkaline phosphatase, bilirubin)
- renal function parameters (creatinine, urea, cystatine C, eGRF, and uric acid)

Details on collection, handling, storage and transport of samples to central lab will be provided in laboratory instructions prior to study start and filed in the ISF and TMF.

Any values outside of reference range will be rated by the investigators regarding their clinical significance. Laboratory values assessed as clinically significant by the investigator need to be documented as AEs.

9.6.4.3.2 Vitamins and Minerals

Venous blood samples will be collected after overnight fasting for analysis of vitamins (B12, D) and trace elements (Mg, Zn). Details on specimen collection, handling, storage and transport of samples to central lab will be provided in the laboratory instructions prior to study start and filed in the ISF and TMF.

9.6.5 Other Parameters

9.6.5.1 Stool Sample Analyses

Stool samples will be collected for analyses of gut microbiome parameters.

Two stool samples will be collected in the week before the visit V2 and the week before the visit V4.

Subjects will be provided with the respective material and instructions for specimen collection, handling, storage and transport prior to study start; the documentation will be filed in the ISF and TMF.

For gut microbiome analyses, generation of data and data analyses will be performed at central lab according to prespecified procedures and analyses plan (generated before unblinding the assignment of the investigational product groups) and the results reported in a distinct report.

Aliquots of samples will be stored for later putative microbiome analyses, for a maximum of 2 years after completion of the clinical phase, and subsequently destroyed after consent of the Sponsor.

In the exceptional case that a subject does not provide the sample at baseline (V2), he/she would still be considered eligible for the study.

9.6.5.2 Rating of Subject Satisfaction and Ease of Use

At study end, the subjects will report to the study staff their assessment of subject satisfaction and the ease of use of study treatment by answering the following questions:

- Is the product easy to use? (very easy/easy/moderately easy/difficult)
- Is the instruction for use easy to follow? (very easy/easy/moderately easy/difficult)
- Do you feel safe using this product? (yes/no)
- Are you satisfied with this product? (yes/no)
- Would you recommend this product to your prediabetic/diabetic friend? (yes/no)
- Has your quality of life improved since you started using this product? (yes/no)

9.6.6 Comment on Methodology Deviating from Normal Clinical Practice

The following methods may be considered as deviating from normal clinical practice:

- Pregnancy test (urine) for women of childbearing potential
- SF-12
- Blood draws for analyses of vitamin/mineral levels
- Assessment of ease of use of study treatment by the subject
- Assessment of AEs/DDs
- Stool sample collection/assessment

9.7 Adverse Events and Device Deficiencies

This section includes information about safety reporting, including definitions of AEs and SAEs, DDs, procedures, and timelines for reporting. The definitions in this section are in accordance with ISO 14155:2020, European Medical Device Regulation (MDR) 2017/745 and the guidance document Safety reporting in clinical investigations of medical devices under the Regulation (EU) 2017/745 (MDCG 2020-10/1).

In this study, there is no active comparator. The term ”investigational medical device“ in this section includes the blinded control treatment with the placebo and thus, all assessments/procedures described for the “investigational medical device” do inherently also apply to the placebo.

The definitions and procedures for safety reporting are presented in the sections below. It is of utmost importance that all staff involved in the investigation are familiar with the definitions and procedures described and it is the responsibility of the investigator to ensure this.

9.7.1 Definitions

<u>Adverse Event (AE)</u>

**Adverse event (AE)**
AE is any untoward medical occurrence, unintended disease or injury, or untoward clinical signs (including abnormal laboratory findings) in subjects, users or other persons, whether or not related to the IMD and whether anticipated or unanticipated.<br>Note 1: This definition includes events related to the IMD or the comparator.<br>Note 2: This definition includes events related to the procedures involved.<br>Note 3: For users or other persons, this definition is restricted to events related to the use of investigational medical devices or comparators.

<u>Adverse Device Effect (ADE)</u>

**Adverse device effect (ADE)**
ADE is any adverse event related to the use of an investigational medical device.<br>Note 1: This definition includes AEs resulting from insufficient or inadequate IFU, deployment, implantation, installation, or operation, or any malfunction of the investigational medical device.<br>Note 2: This definition includes any event resulting from use error or from intentional misuse of the investigational medical device.<br>Note 3: This includes ‘comparator’ if the comparator is a medical device.

Malfunction

Malfunction is any failure of an IMD to perform in accordance with its intended purpose when used in accordance with the IFU or CIP, or IB.

Use error

Use error is any user action or lack of user action while using the medical device that leads to a different result than that intended by the manufacturer or expected by the user.

Note 1: Use error includes the inability of the user to complete a task.

Note 2: Use errors can result from a mismatch between the characteristics of the user, user interface, task or use environment.

Note 3: Users might be aware or unaware that a use error has occurred.

Note 4: An unexpected physiological response of the patient is not by itself considered a use error.

Note 5: A malfunction of medical device that causes an unexpected result is not considered a use error.

Device Deficiency (DD)

**Device deficiency (DD)**
DD is any inadequacy of a medical device with respect to its identity, quality, durability, reliability, usability, safety, or performance.<br>Note 1: DDs include malfunctions, use errors, and inadequacy in the information supplied by the manufacturer including labelling.<br>Note 2: This definition includes DDs related to the IMD or the comparator.

Serious Adverse Event (SAE)

**Serious adverse event (SAE)**
AE that led to any of the following:<br>a) death,<br>b) serious deterioration in the health of the subject, users, or other persons as defined by one or more of the following:<br>1) a life-threatening illness or injury, or<br>2) a permanent impairment of a body structure or a body function including chronic diseases, or<br>3) in-patient or prolonged hospitalization, or

4) medical or surgical intervention to prevent life-threatening illness or injury, or permanent impairment to a body structure or a body function,

c) fetal distress, fetal death, a congenital abnormality, or birth defect including physical or mental impairment.

Planned hospitalization for a pre-existing condition, or a procedure required by the CIP, without serious deterioration in health, is not considered a SAE.

Serious Adverse Device Effect (SADE)

**Serious adverse device effect (SADE)**
ADE that has resulted in any of the consequences characteristic of a SAE.

Unanticipated Serious Adverse Device Effect (USADE)

**Unanticipated serious adverse device effect (USADE)**
SADE which by its nature, incidence, severity, or outcome has not been identified in the current risk assessment.<br>Anticipated serious adverse device effect (ASADE) is an effect which by its nature, incidence, severity, or outcome has been identified in the risk assessment.

Anticipated Serious Adverse Device Effect (ASADE)

An ASADE is an effect which by its nature, incidence, severity or outcome has been identified in the risk analysis report.

Currently, there are no AE types identified as being critical to the evaluation of the results of this clinical investigation.

For the purpose of this study, no SADE are considered anticipated.

Serious health threat

**Serious health threat**
Signal from any AE or DD that indicates an imminent risk of death or a serious deterioration in the health in subjects, users or other persons, and that requires prompt remedial action for other subjects, users or other persons.<br>This would include events that are of significant and unexpected nature such that they become alarming as a potential serious health hazard or possibility of multiple deaths occurring at short intervals.

9.7.2 Collecting and Recording of AEs and DDs

All AEs will be documented from Screening (after signing the ICF).

In general, all A(D)Es, SA(D)Es must be documented in the source data and in eCRFs from the time of signing informed consent until the last clinical investigation visit (i.e., up to Phone call 4, follow-up). DDs must be documented from Visit 2 (i.e., start of treatment) up to Visit 4 (i.e., end of treatment). The subjects will be instructed that they must report any relevant events/observations that may, upon investigator assessment, qualify as A(D)E, SA(D)E, and/or DDs to the investigator.

At each visit, the investigator records the following events in the corresponding part of the eCRF: all AEs and DDs (with or without SADE potential):

- observed
- those based on events/observations reported by subjects upon questioning

If an event occurs, the investigator will assess and record the seriousness, relationship to the investigational device/procedure or comparator, time of occurrence and duration of the observed event, treatment, and resolution/outcome in the study documentation (eCRF). All necessary measures are to be taken to determine the cause of the event and its possible connection to the study.

If a medical device is suspected of having caused a SAE, investigator and principal investigator shall ensure that the IMD is not discarded until the assessment of the competent higher federal authority has been completed.

9.7.3 Assessment of Severity/Intensity

The severity (intensity) of an event or effect is a relative estimate, made by the investigator or qualified designee, based on the comparison with the most severe case encountered in past training and clinical experience.

Each event or effect will be classified in one of the three following categories and will for retrospective documentation (when the AE occurred between the visits) represent the maximum intensity reported during the evaluation period in question.

Mild: asymptomatic or mild symptoms; clinical or diagnostic observations only; intervention not indicated.

Moderate: minimal, local or non-invasive intervention indicated, limiting age-appropriate instrumental activities of daily living.

Severe: severe or medically significant but not immediately life-threatening: hospitalization or prolongation of hospitalization indicated; disabling; limiting self-care activities of daily living.

Comment: The term ‘severe’ is often used to describe the intensity (severity) of a specific event. This is not the same as ’serious’, which is based on patient/event outcome or action criteria.

9.7.4 Assessment of Causal Relationship

For each event, the investigator has to give a statement whether there is a reasonable causal relationship to the IMD or study procedures. The expression ‘reasonable causal relationship’ means to convey in general that there is evidence or argument to suggest a causal relationship to the IMD or study procedures. A gradual assessment will be applied as advised by MDCG 2020-10/1. As causal relationship regarding safety reporting to Competent Authorities the following qualify: ‘causal (relationship)’, ‘probable’ and ‘possible’ qualify. Only ’not related’ does not.

The causality assessment as per MDCG 2020-10/1 is performed as follows:

Not related: Relationship to the device, comparator or procedure can be excluded, when:

- the event has no temporal relationship with the use of the investigational device, or the procedures related to application of the investigational device
- the SAE does not follow a known response pattern to the medical device (if the response pattern is previously known) and is biologically implausible
- the discontinuation of medical device application or the reduction of the level of activation/exposure – when clinically feasible – and reintroduction of its use (or increase of the level of activation/exposure), do not impact on the SAE
- the event involves a body-site or an organ that cannot be affected by the device or procedure
- the SAE can be attributed to another cause (e.g., an underlying or concurrent illness/clinical condition, an effect of another device, drug, treatment or other risk factor)
- the event does not depend on a false result given by the investigational device used for diagnosis, when applicable

In order to establish the non-relatedness, not all the criteria listed above might be met at the same time, depending on the type of device/procedures and the SAE.

Possible: The relationship with the use of the investigational device or comparator, or the relationship with procedures, is weak but cannot be ruled out completely. Alternative causes are also possible (e.g., underlying or concurrent illness/clinical condition or/and an effect of another device, drug or treatment). Cases where relatedness cannot be assessed, or no information has been obtained should also be classified as possible.

Probable: The relationship with the use of the investigational device or comparator, or the relationship with procedures, seems relevant and/or the event cannot be reasonably explained by another cause.

Causal relationship: the SAE is associated with the investigational device, comparator or with procedures beyond reasonable doubt when:

- the event is a known side effect of the product category the device belongs to or of similar devices and procedures
- the event has a temporal relationship with the investigational device use/application or procedures
- the event involves a body-site or organ that
  - the investigational device or procedures are applied to,
  - the investigational device or procedures have an effect on
- the SAE follows a known response pattern to the medical device (if the response pattern is previously known)
- the discontinuation of medical device application (or reduction of the level of activation/exposure) and reintroduction of its use (or increase of the level of activation/exposure), impact on the SAE (when clinically feasible)
- other possible causes (e.g., an underlying or concurrent illness/ clinical condition or/and an effect of another device, drug or treatment) have been adequately ruled out
- harm to the subject is due to error in use
- the event depends on a false result given by the investigational device used for diagnosis, when applicable

In order to establish the relatedness, not all the criteria listed above might be met at the same time, depending on the type of device/procedures and the AE.

9.7.5 Assessment of Outcome

The outcome of the AE must be documented and can be as follows:

Resolved without sequelae: Fully recovered.

Resolved with sequelae: Partially recovered (e.g., event resolved but residual permanent disturbance of health).

Ongoing: AE not yet resolved, or no other outcome can be defined; including AEs ‘resolving’ or ‘not resolved’ at the end of the observation period.

Fatal: Death, which is reasonably related to this specific reported event/reaction. If the death is not reasonably related to this event/reaction, the outcome reached at time of death has to be given (e.g., ongoing). Please mind that death is always the outcome of an event and will not be accepted as diagnosis. Instead, the diagnosis leading to death has to be provided.

Unknown: Only if absolutely no information on the patient's outcome can be collected, e.g., in the case the patient is lost for follow up.

9.7.6 Reporting of SAE/SADE and DDs with SADE potential

9.7.6.1 Reporting by the Investigator

The following events are considered reportable events and shall be reported without delay as defined in MDCG 2020-10/1:

a) Any SAE that has a causal relationship with the investigational device, the comparator or the investigation procedure or where such causal relationship is reasonably possible

b) Any DD that might have led to a SAE if appropriate action had not been taken, intervention had not occurred, or circumstances had been less fortunate

c) Any new findings in relation to any event referred to in points a) and b)

The Sponsor or delegate will ensure that all sites are trained and consequently monitored for adequate safety reporting.

Initial reports as well as follow-up information need to be documented immediately after becoming aware (in case of justified delay not later than 3 calendar days after the occurrence of the event) within the eCRF. All SAEs that occur for any reason during the study, will, even when the cause is not connected to the use of the device, be reported by the investigator as specified in the form "Report on serious adverse event" (in the ISF) immediately after becoming aware (in case of justified delay not later than 3 calendar days) to the CRO vigilance responsible.

In case the eCRF is unavailable for reporting a printed version of "Report on serious adverse event" (in the ISF) needs to be used for report to the designated CRO's vigilance responsible. If the event was reported paper-based the investigator must assure that the information will be added to eCRF when the system becomes available again.

The initial reports will be followed up as soon as new relevant information is available, until event resolution, or for 14 calendar days after study close-out/subject withdrawal, whichever comes first. Any subject who cannot be reached will be considered as lost to follow-up (see section 12.2).

The following timelines, starting with the first knowledge by the investigator, will be followed for the documentation/reporting of AEs and DDs:

**Observed Event (according to MDR definition)**	**When**	**How**
SAE/SADE	Immediate report	CRO's vigilance responsible via eCRF (or Email)
DDs with SADE potential	Immediate report	CRO's vigilance responsible via eCRF (or Email)
Non-serious AE/ADE, DD	No immediate reporting, but documentation in the eCRF within 3 days	CRO's vigilance responsible via eCRF (or Email)

The Sponsor will perform 2nd assessment according to ISO 14155:2020 9.2.5 of the reported AEs and DDs. This includes but is not limited to seriousness and relationship to the investigational device and procedures required by the CIP.

ADEs associated with users or other persons can be documented separately from AEs/ADEs associated with the subject, taking into account the data privacy regulations (section 7.7 of ISO14155:2020).

Vigilance responsible:

email: HTsafety@hungarotrial.com

**In case of emergency calls outside office hours (inclusive weekends and bank holidays):**	
**Name:**	**Timea Görcsös**
**Phone:**	**+36 30 572 3366**

9.7.6.2 Reporting to Regulatory Authorities

Reporting to regulatory authorities will be performed by the CRO's vigilance responsible. As defined in applicable national/regional regulations, expedited reporting requirements will follow the following timelines:

Fatal or Life-Threatening USADEs

The authorities should be notified as soon as possible but no later than 7 calendar days after first knowledge by the Sponsor of a USADE where valid case criteria are reported, followed by as complete a report as possible within 15 calendar days of initial receipt by the Sponsor. Further relevant follow-up information should be given to the authorities as soon as possible, but within an additional 8 calendar days.

<u>All Other USADEs</u>

All other USADEs must be reported to the EC as soon as possible but no later than 15 calendar days after first knowledge of the USADE with valid case criteria is reported. Further relevant follow-up information should be given to the authorities as soon as possible, but within 15 calendar days of the initial report.

9.7.6.3 Reporting to Ethics Committees

CRO's vigilance responsible will prepare the expedited report contents and provide the documents for submission to CRO. CRO will then be responsible for performing the submission of the expedited report to the Central and/or Local ECs.

9.7.7 Follow-up Period after an AE

AEs must be followed up until resolution or the follow-up assessment, whichever comes first. At the follow-up visit, information on new AEs, if any, and stop dates for previously reported AEs must be recorded.

It is the responsibility of the Investigator to follow up on all SAEs until the subject has recovered, stabilized, or recovered with sequelae, and to report to the Sponsor all relevant new information using the same procedures and timelines as those for the initial report. Relevant information includes discharge summaries, autopsy reports, and medical consultation.

SAEs spontaneously reported by a subject to the investigator within 30 days after the last follow-up assessment should be reported to the Sponsor even after the clinical investigation has been finished, if, in the judgment of the investigator, there might be an association between the event and the previous use of the IMD or as a result of the investigation procedures.

9.7.8 Procedures in Case of Pregnancy

In case of pregnancy or suspicion of possible pregnancy, the investigational intervention must be stopped immediately, and the subject discontinued from participation in the investigation. Pregnancy itself is not regarded as an AE unless there is a suspicion that the IMD may have interfered with the effectiveness of the contraceptive medication. However, the outcome of all pregnancies (spontaneous miscarriage, elective termination, normal birth or congenital abnormality) must be followed up and documented even after the subject was discontinued from the investigation.

All events of congenital abnormalities/birth defects are SAEs. Spontaneous miscarriages should also be reported and handled as AEs. All outcomes of pregnancy must be reported to the CRO vigilance responsible on the pregnancy outcomes report form.

The Project Manager of the CRO will provide this information within 24 hours of becoming aware to the safety responsible contact.

Vigilance responsible:

email: HTsafety@hungarotrial.com

Any initial report must be followed by a detailed written report.

9.8 Data Quality Assurance

Quality assurance and quality control systems will comprise e.g.:

- Training
- Monitoring visits
- Standard Operating Procedure (SOPs) of the CRO

9.8.1 CIP Adherence

No systematic or prospective deviations from the CIP are tolerated, except to protect the rights, safety and well-being of subjects under emergency circumstances (such deviations have to be documented by the site and reported to the CRO/Sponsor without delay).

CIP deviations will be documented, classified by the monitor and evaluated during data review by the Sponsor, the CRO, and the biometrician to define the study data sets. Procedures for recording, reporting, and analyzing CIP deviations (corrective and preventive actions) will be performed in accordance with the applicable SOPs and addressed in the Monitoring Plan. Any deviations should be documented and explained by the principal investigator.

9.8.2 Monitoring

The purpose of monitoring is to verify that the conduct of the clinical investigation complies with the approved CIP, subsequent amendments and applicable regulations.

Before the initiation of the clinical investigation, a monitor will verify the adequacy of the investigational site and facilities and discuss with the investigator(s) and other personnel involved in the clinical investigation their responsibilities with regard to the clinical investigation.

During the clinical investigation, a monitor will have regular contacts with the investigational site, including visits to verify that all data in the eCRFs are complete, recorded in a timely manner and consistent with source data, that signed and dated ICFs have been obtained from each subject at the time of enrolment and before any clinical study-related procedures are undertaken, that the clinical investigation is being performed according to the CIP, and in line with ISO14155:2020 and applicable regulations. For this purpose, the investigator must ensure that the Monitor has access to medical records. Access to medical records shall also be granted as far as these relate to the study and without jeopardizing research subject integrity. CRFs for all included subjects must be made available to the Monitor for review.

The Investigator and other relevant personnel should be available during the monitoring visit and should devote sufficient time. Further details with respect to monitoring including the extent of source data verification based on risk assessment will be laid out in a Monitoring Plan.

At the end of the clinical investigation a close-out visit will be performed at the investigational site.

9.8.3 Audits and Inspections

Authorized representatives of the Sponsor and/or regulatory authorities, if applicable, may visit the study centers to perform audits or inspections, including source data verification. The purpose of an audit or inspection is to systematically and independently examine all intervention-related activities and documents to determine whether these activities were conducted, and whether data were recorded, analyzed and reported according to the CIP and in line with the ISO 14155:2020, GCP and applicable regulations.

9.8.4 Case Report Forms

An eCRF will be established to collect the study data. Study personnel responsible for entering data into the eCRFs will be trained to use the system.

It is the investigator’s responsibility to maintain adequate and accurate eCRF documentation to record all observations and other data pertinent to the study.

The principal investigator confirms by signature the accuracy of all entries in the eCRF. The eCRF will be checked by the monitor according to the Monitoring plan.

Study data will be stored in an appropriate format in accordance with applicable data protection regulations.

9.8.5 Source Data

All required source data (any medical records as well as all data collected on the subject during the investigation) will be transcribed into the eCRF. The source data entries must allow to assess whether the study including all procedures was conducted according to the CIP. The study logs and questionnaires act both as source data as well as a part of the eCRF. Source data verification log is to be signed at each site at study start and be filed in ISF/TMF.

9.8.6 Training of Investigation Staff

Before inclusion of the first investigational subject the monitor and/or project manager will perform an Initiation Visit at the investigational site. The requirements of the CIP and related documents will be reviewed and discussed, and the investigational staff will be trained in any investigation specific procedures and system(s) utilized. The training will include IMD usage and mitigation of residual risks. Training should be documented. Current, dated, and signed resumes of all study staff should be filed in the ISF and TMF.

It is the responsibility of the investigator to ensure that all personnel involved in the investigation have been adequately trained and are fully informed of all relevant aspects of the investigation and have detailed knowledge of and training in the procedures that are to be executed by them. Any new information of relevance to the performance of this investigation must be forwarded to the staff involved in a timely manner.

The investigator will keep a list of all personnel involved in the investigation together with their function and investigation related duties delegated. A Curriculum Vitae (CV) will be available for all staff delegated investigation-specific duties.

9.9 Statistical Methods and Determination of Sample Size

The principal features of the statistical analysis to be performed are described in this section. A more technical and detailed elaboration of the principal features will be presented in a separate Statistical Analysis Plan (SAP).

9.9.1 Statistical Analysis Plan

A SAP will be finalized prior to the database lock of the study. The SAP contains a detailed description of all statistical methodology utilized. The final SAP will take into account any amendment to the CIP. The final SAP will be reviewed and possibly updated as a result of the blinded data review(s) performed. The updated version(s) will be finalized and signed prior to database lock and before unblinding of study treatment allocation.

9.9.2 Sample Size Calculation

A sample size of n=130 subjects in each group (the active and the control group), will have 80% power to detect a difference in means of -0,7 mmol/mol (the difference between a mean change in HbA1c in the active group and a mean change in HbA1c in the placebo group), assuming that the standard deviation in both treatment groups is 2 mmol/mol using a two-group t-test with a 5% two-sided significance level.

Considering a maximum dropout rate of about 10% of subjects not completing the study, it is therefore planned to randomize a total of 288 subjects (with n = 144 subjects per treatment group). In case of a large number of dropouts/withdrawals, randomization of additional subjects could be considered.

The choice of mean difference is based on previous trial data. In orientation to previously conducted trials, the pre/post change of HbA1c levels at V4 (week 12) compared to V2 (baseline) in the IMD treatment group is expected to be at least -0.7 mmol/mol (Maruthur et al., 2013; Baek et al., 2021). For the placebo control group, no change is presumed (0 mmol/mol). Thus, the difference in pre/post changes between the IMD treatment group and the placebo group is expected to be at least -0.7 mmol/mol.

The choice of the standard deviation in the sample size calculation is based on data from an earlier study with the IMD predecessor SiPore15®. In that study (STAR01, Baek et al., 2021; clinical investigation report, on file with the Sponsor) the observed standard deviation for the change in HbA1c over 12 weeks of treatment was 1.51 mmol/mol. It is realized that the observed standard deviation may be low in relation to what can be expected in the planned study. In order to compensate for a possible greater standard deviation in the present larger scale study, a standard deviation of 2.0 mmol/mol will be used in the sample size calculations.

With the planned required sample size of n = 130 subjects per treatment group completing the study, the table below shows the probability of observing at least one SADE (in one treatment group) when the expected probability of the SADE is 1%, 1.5%, and 2%.

	**Case 1**	**Case 2**	**Case 3**
Probability of observing at least one event	72.9%	86.0%	92.8%
Actual probability of event	1%	1.5%	2%
Number of subjects studied (n)	176	176	176

9.9.3 Analysis Sets

The study plans to use the following analysis sets:

**Analysis Set**	**Definition**
Enrolled set (ES):	The ES includes all subjects who signed the ICF.
Randomized set (RS):	The RS includes all subjects who were randomized.
Safety set (SS):	The SS includes all randomized subjects who received at least 1 dose of study treatment.
Full analysis set (FAS):	The FAS includes all randomized subjects who received at least 1 dose of study treatment and have both a baseline and at least 1 postbaseline measurement of HbA1c values.
Per-protocol set (PPS):	The PPS includes a subset of subjects in the FAS who completed the study without any important deviations from the CIP and its procedures.

The classification of each patient with respect to each analysis set will be done prior to database lock.

9.9.4 General Principles

Continuous variables will be summarized as number of patients (n), mean, median, standard deviation, quartile 1 and 3 and range (min, max) by visit. The change from baseline at each

respective visit will also be presented. Discrete (categorical/ordinal) variables will be summarized in frequency tables (frequency and proportion) by visit.

Graphical presentations will be used as appropriate.

9.9.5 Primary Endpoint

The primary objective is to evaluate if the IMD treatment leads to a reduction of HbA1c (related to baseline) in comparison to placebo.

The primary endpoint is defined as the difference in changes in HbA1c levels from V2 (baseline) to V4 (week 12) between IMD treatment group and placebo group. A higher reduction of HbA1c (related to baseline) in comparison to placebo corresponds to a better clinical performance.

The primary analysis will be conducted using a two-sided test with $\alpha = 0.05$ (significance level α=5%), using the following hypotheses:

- H_0: $\mu_{active} = \mu_{placebo}$, i.e., no difference between IMD treatment group and placebo control group with respect to the primary endpoint,
- H_A: $\mu_{active} \neq \mu_{placebo}$, i.e., difference between IMD treatment group and placebo control group with respect to the primary endpoint,

where H_0 is the null hypothesis and H_A is the alternative hypothesis.

μ_{active} and $\mu_{placebo}$ are,
μ_{active} = expected change in HbA1c levels at V4 (week 12) compared to V2 (baseline) for the active group treated with IMD,
$\mu_{placebo}$ = expected change in HbA1c levels at V4 (week 12) compared to V2 (baseline) for the control group treated with placebo.

The analysis will be conducted using an ANCOVA model. The model will include baseline HbA1c at V2 as covariate and treatment group, BMI at V1 (<30 or ≥ 30 kg/m2), HbA1c level at V1 (<48 or ≥48 mmol/mol) and site as factors. The two-sided p-value will be considered statistically significant if it is below 5%.

The primary analysis will be conducted on both FAS and PPS populations. The FAS is considered the main analysis while the PPS is only supportive.

If the underlying assumptions of the ANCOVA is clearly violated, the non-parametric analysis Wilcoxon rank sum test will be performed and reported (further described in the SAP).

9.9.6 Main Secondary Endpoint

The CIP defines the following endpoint as main secondary endpoint:

Difference in changes in body weight assessed on-site from V2 (baseline) to V4 (week 12) between IMD treatment group and placebo group.

All statistical analyses proposed in the CIP and the SAP will be reported in the CIR, regardless if the primary endpoint reaches significance.

The statistical analysis will be an ANCOVA analysis based on FAS. The model will include body weight value at V2 as covariate and treatment group, BMI at V1 (<30 or ≥ 30 kg/m2), HbA1c level at V1 (<48 or ≥48 mmol/mol) and site as factors.

9.9.7 Further Secondary Endpoints

The secondary endpoints, difference between IMD treatment group and placebo group in changes from V2 (baseline) to V4 (week 12) in:

- HOMA-IR
- Total cholesterol (TC)
- FBI
- FBG
- LDL-C
- SAD
- TG
- waist-hip-ratio
- body fat content/mass, fat free mass (assessed by BIA)
- SF-12

will be tested according to the order given above.

Detailed description of the analyses will be given in the SAP.

All analyses on the further secondary endpoints will be based on FAS.

9.9.8 Exploratory Endpoints

Detailed description of the analyses for exploratory endpoints will be given in the SAP.

All exploratory endpoints will be analyzed in a non-hierarchical procedure using FAS.

9.9.9 Analysis of Safety and Tolerability

Safety evaluations will be based on AEs, ADEs, DDs, safety laboratory parameters, vital signs, physical examinations, and assessment of serum levels of vitamin and minerals.

All safety evaluations will be done using SS population.

All reported AEs with onset during the treatment phase (i.e., treatment-emergent AEs) will be included in the analysis. For each AE the number and percentage of subjects who

experienced at least one pertinent event will be summarized using Medical Dictionary for Regulatory Activities (MedDRA) primary SOCs and Preferred Terms (PTs) (also including the break down by intensity and relationship to the study treatment and to the procedure).

Any treatment-emergent AEs with a causal relationship to study treatment (including those rated as possibly or probably related) will be considered ADEs. The number and percentage of subjects with a given serious and non-serious adverse device effect will be displayed in a frequency table by MedDRA Primary SOC and PT.

The SAP will provide more details on the summaries and presentations of safety evaluations.

9.9.10 Treatment Compliance

Percent compliance to the study treatment will be calculated as 100 x number of actual stick packs taken / number of expected stick packs taken. The number of actual stick packs taken will be calculated as number of stick packs dispensed – number of unused stick packs returned. If unused stick packs are not returned, the number of unused stick packs returned will be considered 0 for the compliance calculation. The number of expected stick packs taken is equal to 12*7*3, for 12 weeks of treatment with 3 stick packs per day. Percent compliance to the study treatment will be summarized by treatment group based on FAS.

9.9.11 Interim Analysis

No interim analysis is planned in this study.

9.9.12 Subgroup Analysis

Subgroup analyses are planned to be performed for the primary endpoint and the main secondary endpoint using FAS.

Subgroup analyses will be performed for subgroups HbA1c level at V1 (<48 or ≥48 mmol/mol; < 6.5 or ≥6.5% - according to DCCT, 1987), BMI at V1 (<30 or ≥ 30 kg/m2).

Other subgroup analyses might be added in the SAP.

9.9.13 Sensitivity Analyses

Sensitivity analyses might be added to the SAP, for example, addressing a large number of drop-outs. A sensitivity analysis will be conducted for the primary endpoint where missing values will be imputed. The type of imputation will be specified in the SAP.

9.10 Data Management

All collected data will be documented in the eCRF and checked by the monitor at regular intervals for plausibility and completeness according to the Monitoring Plan. The designated site personnel will be instructed to make any required corrections or additions.

Any inconsistencies detected in the eCRF will be resolved during the data cleaning process by using data clarification forms (queries). Once the database is declared to be complete and accurate, it will be locked.

Concomitant medications entered into the database will be coded using the WHO Drug Reference List. Medical history and AEs will be coded using the MedDRA terminology.

Laboratory samples will be processed centrally by a vendor and the results will be sent electronically to the designated CRO.

All electronic data capture systems will be validated. eCRF will be validated according to Data Validation Plan and user acceptance tests will be performed prior to release, in accordance with the applicable SOP.

10 DEVICE ACCOUNTABILITY

The Sponsor and the investigator will keep records documenting the location of the IMD and placebo from shipment via the investigation sites to study subjects, until they have been returned. Accountability will be documented by a shipment log stored at the Sponsor delegate and in a device accountability log at the investigation site. To ensure traceability, there is a batch number system in place according to EN ISO 13485.

The device accountability at site will include batch number, dates when delivered, handed out to subjects, returned from subjects, and returned to Sponsor. The monitor will verify the accountability process at each site during the site monitoring visits.

DocuSign Envelope ID: DC97F66E-B279-4A14-AC63-5C291FAB6C7D

11 EMERGENCY PROCEDURES

The Principal Investigator is responsible for ensuring that procedures and expertise are available at the investigational site to handle medical emergencies during the investigation.

In the case of a medical emergency, the investigator may contact the Medical Monitor (9.7.6 Contact details)

Detailed SAE reporting procedures are included in Section 9.7.6.

12 MANAGEMENT OF THE INVESTIGATION

12.1 CIP Amendments

All amendments to the CIP need agreement between the Sponsor and the coordinating investigator. Every substantial amendment to the CIP or the subject information including the ICF needs to be approved by the EC and competent authority prior to implementation.

Under emergency circumstances, deviations from the CIP to protect the rights, safety and well-being of human subjects may proceed without prior approval of the Sponsor and the EC; such deviations shall be documented and reported to the Sponsor without delay (see section 9.8.1).

12.2 Discontinuation of the Investigation

Subjects may withdraw their consent and discontinue their participation in the clinical study at any time, without providing a reason for discontinuation. The investigator may intermit or prematurely terminate the clinical investigation for individual subjects. Further, the Sponsor has the right to terminate this clinical investigation prematurely.

12.2.1 Withdrawal of Subjects

Reasons for subject's withdrawal may be for example:

- AE
- Intolerance of the study treatment
- Required additional medical attention or therapy, which could influence the safety of the subject or the results of the study
- Clinically significant illness or intake of concurrent medication according to exclusion criteria, which could influence the results of the study
- Pregnancy
- Subject is not compliant with study requirements (according to investigator judgement)
- Withdrawal of informed consent

The reason, time and specific details of a subject's withdrawal will be documented in the eCRF. Any subject terminating the study prematurely will be asked to undergo the end-of-study on site visit (visit V4), to be documented in the eCRF. If the subject does not appear on this visit, reasonable attempts to reach the subject (e.g., per phone call or electronic message) will be documented; subjects who cannot be reached will be considered as lost to follow-up. With regard to study discontinuation (e.g., due to AEs or intolerance of the study

treatment) the investigator must ensure the additional documentation within the AE section of eCRF, as applicable.

The IMD should be returned to the site by the subject.

Subjects who discontinue the study for safety reasons should, whenever possible, be seen and assessed by the investigator and be medically advised as appropriate.

Subjects who withdraw from the study will not be replaced.

12.2.2 Study Discontinuation Criteria

If the clinical investigation must be discontinued, each subject is to be treated as described above.

The Sponsor has the right to suspend or prematurely terminate this clinical investigation, e.g., for the following reasons:

- Serious, insolvable problems with the quality of the data
- Serious or repeated deviations on the part of an investigator
- Unforeseeable circumstances at the investigational sites, which require discontinuation of the study (at an individual site and/or in general)
- Unacceptable risks or serious health threat
- New scientific or medical knowledge

12.3 Reporting and Publication of Investigation Results

A Clinical Investigation Report, in compliance with ISO 14155:2020, Annex D, describing the conduct of the investigation, the statistical analysis performed, and the results obtained, will be prepared. The analyses and results from the exploratory objectives will not be part of the report but reported separately.

The Clinical Investigation Report will be submitted to the Competent Authorities within 12 months from end of investigation or be available on request, as per local requirements.

Before recruitment starts, the Sponsor will register the clinical investigation in a publicly accessible database and maintain the content up-to-date during the investigation as applicable.

The results of this clinical investigation may be published with the written agreement of the study Sponsor.

12.4 Disclosure and Confidentiality

All unpublished information concerning the IMD, and research carried out by the Sponsor, including patent applications, manufacturing processes, basic scientific data, etc., is

considered confidential and the sole property of the Sponsor. Disclosure to third parties must be limited to those undertaking legitimate peer review of the scientific and ethical aspects of the investigation and to those participating, including the recipients of IMD, so that customary medical care and informed consent can be achieved.

12.5 Archiving

The medical records of subjects shall be retained in accordance with local legislation and in accordance with the maximum period of time permitted by the site. Any transfer of responsibility for storage of the records should be documented and the Sponsor should be informed in writing. The study data is the sole property of the Sponsor and should not be made available in any form to third parties without written permission from the sponsor.

At the end of the study, electronic data are kept at the Sponsor and an independent copy is kept at the study center as part of the ISF. All records pertaining to the conduct of the study, including signed eCRFs, ICFs, study product accountability records, source documents, and other study documentation must be retained for as long as is specified in the Clinical Investigation Agreement or for at least 10 years after the premature termination or completion of the investigation. Measures should be taken to prevent accidental or premature destruction of these documents (e.g., protection against damage and unauthorized access, preferably by storage in a fire-proof cabinet). It is the Investigator's responsibility to inform the Sponsor in writing if the Investigator file is moved or if the responsibility for the documents is transferred to someone else.

The Sponsor will retain the TMF in line with applicable regulations or for at least 10 years after the clinical investigation has ended, or, in the event that the device is subsequently placed on the market, at least 10 years after the last device has been placed on the market.

12.6 Insurance

All subjects participating in this clinical investigation will be insured through the Sponsor. A copy of the insurance certificate will be provided to each investigator and will be filed in the ISF at the sites and in the TMF.

12.7 Financing and Agreements

The study is organized and financed by the Sponsor of this clinical investigation, Sigrid Therapeutics AB.

An appropriate contract will be established between the Sponsor and each site. Each responsible investigator is obliged to comply with the commitments and obligations set out in the signed agreement. The site will receive a fee for each study participant who completes the study in accordance with the agreement. The study doctor and the study staff have no personal or financial ties to the Sponsor, other than in connection with sponsored studies that they conduct, or services that they provide to the Sponsor.

Agreements between Sponsor and the investigational site must be in place before any investigation-related procedures can take place, or subjects are enrolled.

13 REFERENCES

American Diabetes Association (ADA); Standards of Medical Care in Diabetes—2022 Abridged for Primary Care Providers. Clin Diabetes. 2022;40(1):10–38.

Baek J, Robert-Nicoud G, Herrera Hidalgo C, Borg ML, Iqbal MN, Berlin R, Lindgren M, Waara E, Uddén A, Pietiläinen K, Bengtsson T. Engineered mesoporous silica reduces long-term blood glucose, HbA1c, and improves metabolic parameters in prediabetics. Nanomedicine (Lond). 2022;17(1):9-22.

Bouchoucha M, Uzzan B, Cohen R. Metformin and digestive disorders. Diabetes Metab. 2011;37(2):90-96.

Diabetes Control and Complications Trial (DCCT): results of feasibility study. The DCCT Research Group. Diabetes Care. 1987;10(1):1-19.

Diabetes Prevention Program (DPP) Research Group. Effects of withdrawal from metformin on the development of diabetes in the diabetes prevention program. Diabetes Care. 2003;26(4):977-80.

Dujic T, Zhou K, Donnelly LA, Tavendale R, Palmer CN, Pearson ER. Association of organic cation transporter 1 with intolerance to metformin in Type 2 diabetes: a GoDARTS study. Diabetes. 2015;64(5):1786-93.

Echouffo-Tcheugui JB, Selvin E. Prediabetes and What It Means: The Epidemiological Evidence. Annu Rev Public Health. 2021;42:59-77.

EFSA Panel on Dietetic Products, Nutrition and Allergies (NDA); Guidance on the scientific requirements for health claims related to appetite ratings, weight management, and blood glucose concentrations. EFSA Journal. 2012;10(3):2604.

European Medicines Agency (EMA), Committee for Medicinal Products for Human Use (CHMP). Guideline on clinical investigation of medicinal products in the treatment or prevention of diabetes mellitus, Draft. CHMP/EWP/1080/00 Rev. 2. 2018.

Florez H, Luo J, Castillo-Florez S, et al. Impact of metformin-induced gastrointestinal symptoms on quality of life and adherence in patients with type 2 diabetes. Postgrad Med. 2010;122(2):112-120.

Food and Drug Administration (FDA). Feasibility and Early Feasibility Clinical Studies for Certain Medical Devices Intended to Therapeutically Improve Glycemic Control in Patients with Type 2 Diabetes Mellitus. Draft Guidance for Industry and Food and Drug Administration Staff. Rockville, MD. 2022.

Grundy SM. Pre-diabetes, metabolic syndrome, and cardiovascular risk. J. Am. Coll. Cardiol. 2012; 59(7),635–643.

Hagman E, Elimam A, Kupferschmidt N, Ekbom K, Rössner S, Iqbal MN, Johnston E, Lindgren M, Bengtsson T, Danielsson P. Oral intake of mesoporous silica is safe and well tolerated in male humans. PLoS One. 2020;15(10):e0240030.

International Classification of Diseases, 10th Revision, Clinical Modification; ICD-10-CM codes. Available from https://www.icd10data.com (accessed Mar 30, 2023).

International diabetes federation (IDF): IDF diabetes atlas - 10th edition, 2021.

International Expert Committee. International Expert Committee report on the role of the A1C assay in the diagnosis of diabetes. Diabetes Care. 2009;32(7):1327-34.

Katz A, Nambi SS, Mather K, Baron AD, Follmann DA, Sullivan G, Quon MJ. Quantitative insulin sensitivity check index: a simple, accurate method for assessing insulin sensitivity in humans. J Clin Endocrinol Metab. 2000;85(7):2402-10.

Kirpichnikov D, McFarlane SI, Sowers JR. Metformin: an update. Ann Intern Med. 2002;137(1):25-33.

Kivimäki M, Kuosma E, Ferrie JE, Luukkonen R, Nyberg ST, Alfredsson L, Batty GD, Brunner EJ, Fransson E, Goldberg M, Knutsson A, Koskenvuo M, Nordin M, Oksanen T, Pentti J, Rugulies R, Shipley MJ, Singh-Manoux A, Steptoe A, Suominen SB, Theorell T, Vahtera J, Virtanen M, Westerholm P, Westerlund H, Zins M, Hamer M, Bell JA, Tabak AG, Jokela M. Overweight, obesity, and risk of cardiometabolic multimorbidity: pooled analysis of individual-level data for 120 813 adults from 16 cohort studies from the USA and Europe. Lancet Public Health. 2017;2(6):e277-e285.

Knowler WC, Barrett-Connor E, Fowler SE, Hamman RF, Lachin JM, Walker EA, Nathan DM; Diabetes Prevention Program Research Group. Reduction in the incidence of type 2 diabetes with lifestyle intervention or metformin. N Engl J Med. 2002;346(6):393-403.

Kupferschmidt N, Csikasz RI, Ballell L, Bengtsson T, Garcia-Bennett AE. Large pore mesoporous silica induced weight loss in obese mice. Nanomedicine (Lond). 2014;9(9):1353-62.

Little RR, Rohlfing C, Sacks DB. The National Glycohemoglobin Standardization Program: Over 20 Years of Improving Hemoglobin A1c Measurement. Clin Chem. 2019;65(7):839-848.

Maruthur NM, Ma Y, Delahanty LM, Nelson JA, Aroda V, White NH, Marrero D, Brancati FL, Clark JM; Diabetes Prevention Program Research Group. Early response to preventive strategies in the Diabetes Prevention Program. J Gen Intern Med. 2013;28(12):1629-36.

Matthews DR, Hosker JP, Rudenski AS, Naylor BA, Treacher DF, Turner RC. Homeostasis model assessment: insulin resistance and B-cell function from fasting plasma glucose and insulin concentrations in man. Diabetologia. 1985;28(7):412-9.

May KL, Pham AC, Ramirez G, Herrera-Hidalgo C, Naeem Iqbal M, Robert-Nicoud G, Clulow AJ, Bengtsson T, Boyd BJ. Towards mesoporous silica as a pharmaceutical treatment for obesity - impact on lipid digestion and absorption. Eur J Pharm Biopharm. 2022;173:1-11.

Nuha A. ElSayed, Grazia Aleppo, Vanita R. Aroda, Raveendhara R. Bannuru, Florence M. Brown, Dennis Bruemmer, Billy S. Collins, Marisa E. Hilliard, Diana Isaacs, Eric L. Johnson, Scott Kahan, Kamlesh Khunti, Jose Leon, Sarah K. Lyons, Mary Lou Perry, Priya Prahalad, Richard E. Pratley, Jane Jeffrie Seley, Robert C. Stanton, Robert A. Gabbay; on behalf of the

American Diabetes Association, 3. Prevention or Delay of Type 2 Diabetes and Associated Comorbidities: *Standards of Care in Diabetes—2023*. *Diabetes Care*. 2023;46 (Supplement_1): S41–S48.

Rinde M, Kupferschmidt N, Iqbal MN, Robert-Nicoud G, Johnston EV, Lindgren M, Bengtsson T. Mesoporous silica with precisely controlled pores reduces food efficiency and suppresses weight gain in mice. Nanomedicine (Lond). 2020;15(2):131-144.

Sampaio LR, Simões EJ, Assis AM, Ramos LR. Validity and reliability of the sagittal abdominal diameter as a predictor of visceral abdominal fat. Arq Bras Endocrinol Metabol. 2007;51(6):980-6.

Schlesinger S, Neuenschwander M, Ballon A, Nöthlings U, Barbaresko J. Adherence to healthy lifestyles and incidence of diabetes and mortality among individuals with diabetes: a systematic review and meta-analysis of prospective studies. J Epidemiol Community Health. 2020;74(5):481-487.

SiPore21® Instructions for use (IFU). Sigrid Therapeutics AB, Internal Document (TF 02-02), 2023.

SiPore21® Investigator's brochure (IB). Sigrid Therapeutics AB, Internal Document (DOC-001), 2023.

Tabák AG, Herder C, Rathmann W, Brunner EJ, Kivimäki M. Prediabetes: a high-risk state for diabetes development. Lancet. 2012;379(9833):2279-90.

US Food & Drug Administration (FDA), Code of Federal Regulations, Title 21, Volume 3, 21CFR172.480

US Food & Drug Administration (FDA), Code of Federal Regulations, Title 21, Volume 3, 21CFR182.90; GRAS Notices No. GRN 000321 and GRN 000554

US Agency for Toxic Substances and Disease Registry (ATSDR). 2019. Toxicological profile for Silica. Atlanta, U.S.

Waara ER, Iqbal MN, Robert-Nicoud G, Benziane B, Vallhov H, Wasik AM, Lindgren M, Hagman E, Rinde M, Kupferschmidt N, Berlin R, Johnston EV, Danielsson P, Bengtsson T. Entrapping Digestive Enzymes with Engineered Mesoporous Silica Particles Reduces Metabolic Risk Factors in Humans. Adv Healthc Mater. 2020;9(11):e2000057.

World Health Organization (WHO). Physical status: the use and interpretation of anthropometry. Report of a WHO Expert Committee. Technical Report Series No. 854. 1995.

World Health Organization (WHO). Abbreviated report of a WHO consultation. Use of glycated hemoglobin (HbA1c) in the diagnosis of diabetes mellitus. 2011.

14 APPENDICES

14.1 Signature Pages

"I agree to the terms of this Clinical Investigation Plan (CIP)."

Sponsor signatory

Sana Alajmovic<br>Co-founder & CEO	DocuSigned by:<br>Sana Alajmovic<br>5964F3BB28C64D8...	24-Jul-2023
Name, title	*Signature*	*Date*

Sponsor Medical Officer

Stephan Rössner<br>Professor Emeritus	DocuSigned by:<br>Stephan Rössner<br>0ADCFBE96308440...	26-jul-2023
Name, title	*Signature*	*Date*

13.1 Signature pages

Coordinating/Principal Investigator(s)

"I agree to the terms of this Clinical Investigation Plan (CIP). I will conduct the investigation in accordance with the procedures specified in the CIP, the ethical principles in the latest version of the Declaration of Helsinki, ICH Good Clinical Practice, ISO 14155:2020 and any other applicable regulatory requirements".

Kirsi Pietiläinen<br>MD Prof.	DocuSigned by:<br>Kirsi Pietiläinen<br>98A0A232BA42402...	25-Jul-2023
Name, title	*Signature*	*Date*

14.2 Declaration of Helsinki

http://www.up.ac.za/media/shared/Legacy/sitefiles/file/45/2875/declarationofhelsinki_fortaleza_brazil2013.pdf

14.3 Short Form 12 Health Survey Questionnaire

SF12 HEALTH SURVEY

ID NUMBER: ☐☐☐☐☐☐☐☐ FORM CODE: **SFH** VERSION: **3.0** 11/07/2017 Event: ____________

0a) Date of Collection ☐☐ / ☐☐ / ☐☐☐☐ 0b) Staff Code ☐☐☐

Instructions: *This form should be completed during the clinic visit. Please read each question carefully.*

The first question is about your health now. Please try to answer as accurately as you can.

1) In general, would you say your health is...
- ☐ Excellent1
- ☐ Very good2
- ☐ Good3
- ☐ Fair4
- ☐ Poor5

Now, please think about the activities that you might do during a typical day. As you read each item, please select whether your health now limits you a lot, limits you a little, or does not limit you at all when doing these activities.

2a) …moderate activities, such as moving a table, pushing a vacuum cleaner, bowling, or playing golf. Does your health now limit you a lot, limit you a little, or not limit you at all?
- ☐ Yes, Limited a lot1
- ☐ Yes, Limited a little2
- ☐ No, Not at all limited3

2b) …climbing several flights of stairs. Does your health now limit you a lot, limit you a little, or not limit you at all?
- ☐ Yes, Limited a lot1
- ☐ Yes, Limited a little2
- ☐ No, Not at all limited3

The following two questions ask you about your physical health and your daily activities.

3a) During the past four weeks, how much of the time have you accomplished less than you would like as a result of your physical health?
- ☐ All of the time1
- ☐ Most of the time2
- ☐ Some of the time3
- ☐ A little of the time4
- ☐ None of the time5

SF-12v2™ Health Survey © 1994, 2002 by Quality Metric Incorporated and Medical Outcomes Trust. All Rights Reserved. SF-12 ® a registered trademark of Medical Outcomes Trust. (SF12v2 Standard, US Version 2.0)

ID NUMBER: [ ] [ ] [ ] [ ] [ ] [ ] [ ] [ ]   FORM CODE: **SFH** VERSION: **3.0** 11/07/2017   Event: ____________

3b) During the past four weeks, how much of the time were you limited in the kind of work or other regular daily activities you do as a result of your physical health?
- ☐ All of the time 1
- ☐ Most of the time 2
- ☐ Some of the time 3
- ☐ A little of the time 4
- ☐ None of the time 5

The following two questions ask you about your emotions and your daily activities.

4a) During the past four weeks, how much of the time have you accomplished less than you would like as a result of any emotional problems, such as feeling depressed or anxious?
- ☐ All of the time 1
- ☐ Most of the time 2
- ☐ Some of the time 3
- ☐ A little of the time 4
- ☐ None of the time 5

4b) During the past four weeks, how much of the time were you limited in the kind of work or other regular daily activities you do as a result of any emotional problems, such as feeling depressed or anxious?
- ☐ All of the time 1
- ☐ Most of the time 2
- ☐ Some of the time 3
- ☐ A little of the time 4
- ☐ None of the time 5

5) During the past 4 weeks, how much did pain interfere with your normal work (including both work outside the home and housework)?
- ☐ Not at all 1
- ☐ A little bit 2
- ☐ Moderately 3
- ☐ Quite a bit 4
- ☐ Extremely 5

The next four questions are about how you feel and how things have been with you during the past 4 weeks. As you read each statement, please select the one answer that comes closest to the way you have been feeling; is it all of the time, most of the time, some of the time, a little of the time, or none of the time?

6a) How much of the time during the past 4 weeks…have you felt calm and peaceful?
- ☐ All of the time 1
- ☐ Most of the time 2
- ☐ Some of the time 3
- ☐ A little of the time 4
- ☐ None of the time 5

SF-12v2™ Health Survey © 1994, 2002 by Quality Metric Incorporated and Medical Outcomes Trust. All Rights Reserved. SF-12 ® a registered trademark of Medical Outcomes Trust. (SF12v2 Standard, US Version 2.0)

ID NUMBER: | | | | | | | | |

FORM CODE: **SFH**
VERSION: **3.0** 11/07/2017

Event: ____________________

6b) How much of the time during the past 4 weeks…did you have a lot of energy?

- ☐ All of the time1
- ☐ Most of the time2
- ☐ Some of the time3
- ☐ A little of the time4
- ☐ None of the time5

6c) How much of the time during the past 4 weeks…have you felt downhearted and depressed?

- ☐ All of the time1
- ☐ Most of the time2
- ☐ Some of the time3
- ☐ A little of the time4
- ☐ None of the time5

7) How much of the time during the past 4 weeks…has your physical health or emotional problems interfered with your social activities (like visiting with friends, relatives, etc.)?

- ☐ All of the time1
- ☐ Most of the time2
- ☐ Some of the time3
- ☐ A little of the time4
- ☐ None of the time5

END OF FORM

SF-12v2™ Health Survey © 1994, 2002 by Quality Metric Incorporated and Medical Outcomes Trust. All Rights Reserved. SF-12 ® a registered trademark of Medical Outcomes Trust. (SF12v2 Standard, US Version 2.0)
