# Supplementary material for: Effect of engineered mesoporous silica particles with tailored pore size on glycaemic control in individuals with prediabetes or type 2 diabetes: a randomised, double-blind, placebo-controlled SHINE trial
Source: eClinicalMedicine. 2026 Jul 2;97:104042. doi: 10.1016/j.eclinm.2026.104042 (PMC13352034; doi:10.1016/j.eclinm.2026.104042)
Supplement: Statistical Analysis Plan_SHINE [file mmc5.pdf]

Statistical Analysis Plan

Randomized, double-blinded, placebo-controlled, multicenter trial investigating performance and safety of the medical device SiPore21® in obese or overweight subjects with elevated blood glucose levels

Clinical Investigation Plan - SITH/001921

Sponsor : Sigrid Therapeutics

Version : 1.1

Issue/Report Date : 26.NOV.2024

TABLE OF CONTENTS

Signature Page 5

Revision History 6

LIST OF ABBREVIATIONS AND DEFINITIONS OF TERMS 8

1. INTRODUCTION 10

2. STUDY OBJECTIVE AND DESIGN 10
   - 2.1 Study Objective 10
     - 2.1.1 Primary Objective 10
     - 2.1.2 Main Secondary Objective 10
     - 2.1.3 Further secondary Objectives 10
     - 2.1.4 Safety objective 10
     - 2.1.5 Explorative objectives 10
   - 2.2 Study Design 11
   - 2.3 Statistical Hypotheses and Sample Size Justification 15

3. GENERAL ANALYSIS DEFINITIONS 16
   - 3.1 Analysis Sets 16
     - 3.1.1 Enrolled Set (ES) 16
     - 3.1.2 Randomized Set (RS) 16
     - 3.1.3 Safety Set (SS) 16
     - 3.1.4 Full Analysis Set (FAS) 16
     - 3.1.5 Per Protocol Set (PPS) 17
   - 3.2 Clinical Investigation Plan deviations 17
   - 3.3 General Principles 17
   - 3.4 Stratifications 17
   - 3.5 Medical Dictionary and version 17
   - 3.6 Software version 17
   - 3.7 Visit Windows 17
   - 3.8 Data Handling Rules 18
     - 3.8.1 Handling of Missing data 18
     - 3.8.2 Handling of Incomplete dates 18
     - 3.8.3 Handling of Invalid Values 18

4. INTERIM ANALYSIS AND DATA MONITORING COMMITTEE REVIEW 19

5. SUBJECT INFORMATION 19
   - 5.1 Disposition Information 19
     - 5.1.1 Variable and/or Definition 19
     - 5.1.2 Analysis Methods 20

5.2 Demographics and Baseline Characteristics 20
5.2.1 Variable and/or Definition 20
5.2.2 Analysis Methods 20
5.3 Medical History 21
5.3.1 Variable and/or Definition 21
5.3.2 Analysis Methods 21
5.4 Prior and Concomitant Medications 21
5.4.1 Variable and/or Definition 21
5.4.2 Analysis Methods 21
5.5 Prior and Concomitant Treatments 21
5.5.1 Variable and/or Definition 21
5.5.2 Analysis Methods 22
5.6 Treatment Compliance 22
5.6.1 Variable and/or Definition 22
5.6.2 Analysis Methods 22

6. EFFICACY 22
6.1 Primary Efficacy Endpoint 23
6.1.1 Variable and/or Definition 23
6.1.2 Analysis Methods 23
6.2 Main Secondary Efficacy Endpoint 24
6.2.1 Variable and/or Definition 24
6.2.2 Analysis Methods 24
6.3 Further Secondary Efficacy Endpoints 24
6.3.1 Variable and/or Definition 25
6.3.2 Analysis Methods 25
6.4 Exploratory Efficacy Endpoints 25
6.4.1 Variable and/or Definition 26
6.4.2 Analysis Methods 27

7. SAFETY 27
7.1 Adverse Events 27
7.1.1 Variable and/or Definition 27
7.1.2 Analysis Methods 28
7.2 Device Deficiencies (DDs) 28
7.2.1 Variable and/or Definition 28
7.2.2 Analysis Methods 28
7.3 Laboratory Tests 29
7.3.1 Variable and/or Definition 29
7.3.2 Analysis Methods 29

7.4 Vital Signs ... 29
7.4.1 Variable and/or Definition ... 29
7.4.2 Analysis Methods ... 29
7.5 Vitamins and Trace Elements ... 30
7.5.1 Variable and/or Definition ... 30
7.5.2 Analysis Methods ... 30
8. CHANGE IN STATISTICAL ANALYSIS METHODS ... 31
9. REFERENCES ... 33
Appendix. TLF Shells ... 33

Signature Page

This document has been reviewed and approved by as below.

Prepared by

**Title(Company): Project Biostatistician (CRScube)**<br>**Name: Daehan Kim**

Reviewed by

**Title(Company): STAT Team Manger (CRScube)**<br>**Name: NaYoon Chang**

Reviewed by

**Title(Company): Senior Consultant Biostatistics (SDS Life Science)**<br>**Name: Daniel Bruce**

Approved by

**Title(Company): Project Manager (Sigrid Therapeutics AB)**<br>**Name: Maria Klockare**

Approved by

**Title(Company): Project Manager (HungaroTrial)**<br>**Name: Matyas Petho**

REVISION HISTORY

<table>
<tr><th>Document Ver.</th><th>Approval Date</th><th colspan="3">Revision Details</th></tr>
<tr><td>1.0</td><td>20.JUN.2024</td><td colspan="3">First Document</td></tr>
<tr><td rowspan="4">1.1</td><td rowspan="4">26.NOV.2024</td><td>Item</td><td>Before (Version No.: 1.0)</td><td>After (Version No.: 1.1)</td></tr>
<tr><td>3.7 Visit Windows</td><td>Applied variable: HbA1c, Glucose, Insulin, Lipid parameters, Physical Measurements</td><td>Applied variable: HbA1c, Glucose, Insulin, Lipid parameters, Physical Measurements, hs-CRP</td></tr>
<tr><td>3.8.1 Handling of Missing data</td><td>A sensitivity analysis will be conducted for the primary endpoint where missing values will be imputed using Multiple imputation.
In the case of safety analysis, raw data will be used as collected.</td><td>A sensitivity analysis will be conducted for the primary and main secondary endpoint where missing values will be imputed using Multiple imputation.
In the case of safety analysis, raw data will be used as collected.

Baseline is defined as the last measurement prior to the first dose of study treatment unless stated otherwise. If all measurements prior to first dose of study drug is missing, then Baseline value is set to missing.</td></tr>
<tr><td>5.6.1 Variable and/or Definition</td><td>(3) Total duration of administration
• Total duration of administration(day) = Study treatment End Date – Study treatment Start Date</td><td>(3) Total duration of administration
• Total duration of administration(day) = Study treatment End Date* – Study treatment Start Date
*Study treatment End Date is regarded as 'Date of Completion/Withdrawal' of 'End of Study' page in the CRF if it is missing.</td></tr>
</table>

		6.2.2 Analysis Methods		**A sensitivity analysis will be conducted where missing data are imputed based on multiple imputations using the same methods as for the primary efficacy sensitivity analysis.**
		6.3 Further Secondary Efficacy Endpoints	Difference between IMD treatment group and placebo group in changes in body weight assessed off site from V2 (baseline) to V4 (week 12) and to PC3 (week 13)	Difference between IMD treatment group and placebo group in changes in body weight assessed off site from V2 (baseline) **to PC1 (week 3) and** to V4 (week 12) and to PC3 (week 13)
		6.3.1 Variable and/or Definition	• FBI is collected as 'Insulin' (~~mg/dL~~) in the Laboratory Tests (Central Lab) of CRF.<br><br>• SF-12 total score<br><br>Body weight assessed off site will be collected as 'Body weight (off site)'.<br>- Changes in body weight assessed off site (kg) = Week 12 (Visit 4) weight or Week 13 (PC3) weight) – Baseline (Visit 2) weight	• FBI is collected as 'Insulin' (**uU/ml**) in the Laboratory Tests (Central Lab) of CRF.<br><br>• SF-12 total score**: FCS(Physical Component Summary), MCS(Mental Component Summary)**<br><br>Body weight assessed off site will be collected as 'Body weight (off site)'.<br>- Changes in body weight assessed off site (kg) = (**Week 3 (PC1) weight or** Week 12 (Visit 4) weight or Week 13 (PC3) weight) – Baseline (Visit 2) weight **(on-site)**
		6.4.1 Variable and/or Definition	• QUICKI = 1/[log(FBI(='Insulin') (mU/l*)) + log(FBG(='Glucose') (~~mmol/l*~~))]	• QUICKI = 1/[log**10**(FBI(='Insulin') (mU/l*)) + log**10**(FBG(='Glucose') (**md/dL**))]

LIST OF ABBREVIATIONS AND DEFINITIONS OF TERMS

Abbreviation	Term
AE	Adverse Event
AC	Atherogenic Coefficient
ADE	Adverse Device Effect
AIP	Atherogenic Index of Plasma
ALAT	Alanine Transaminase
ANCOVA	Analysis of Covariance
ASAT	Aspartate Aminotransferase
ATC	Anatomical Therapeutic Chemical
BIA	Bioelectrical Impedance Analysis
BMI	Body Mass Index
CIP	Clinical Investigation Plan
CRF	Case Report Form
CRR	Cardiac Risk Ratio
CS	Clinically Significant
CSR	Clinical Study Report
ECG	Electrocardiogram
EDC	Electronic Data Capture
ES	Enrolled Set
FAS	Full Analysis Set
FBG	Fasting Blood Glucose
FBI	Fasting Blood Insulin
Gamma-GT	y-glutamyl transpeptidase
GLM	General Linear Model
HbA1c	Glycated Hemoglobin
HC	Hip Circumference
HDL-C	High-density lipoprotein-cholesterol
HOMA-IR	Homeostasis Model Assessment for Insulin Resistance
HOMA-B	Homeostasis Model Assessment for Beta Cell Function
hs-CRP	High Sensitivity C-Reactive Protein
ICF	Informed Consent Form
ICH	International Council for Harmonisation
IMD	Investigational Medical Device
LDL-C	Low-density lipoprotein cholesterol
LOCF	Last Observation Carried Forward
MedDRA	Medical Dictionary for Regulatory Activities
Mg	Magnesium
MMRM	Mixed Model Repeated Measure
NCS	Not Clinically Significant

**Abbreviation**	**Term**
PC	Phone Call
PPS	Per-Protocol Set
PT	Preferred Term
QUICKI	Quantitative Insulin Sensitivity Check Index
RS	Randomized Set
SAD	Sagittal Abdominal Diameter
SADE	Serious Adverse Device Effect
SAE	Serious Adverse Event
SAP	Statistical Analysis Plan
SAS	Statistical Analysis Software
SF-12	Short Form Health Survey (12-Item)
SiPore21®	Gel containing MSP21
SS	Safety Set
SOC	System Organ Class
TC	Total Cholesterol
TG	Triglyceride
TSH	Thyroid-Stimulating Hormone
UK	Unknown
V	Visit
VLDL-C	Very Low-Density Lipoprotein Cholesterol
WC	Waist Circumference
WHODD	World Health Organization Drug Dictionary
Zn	Zinc

1. INTRODUCTION

The Statistical Analysis Plan (SAP) describes the planned statistical analysis and result report of this clinical trial SITH/001921.

SITH/001921 is to evaluate the clinical performance of the Investigational Medical Device (IMD) for blood glucose control and its clinical safety.

The following documents have been reviewed in preparation of this SAP.

- Clinical Investigation Plan of SITH/001921 (v 2.0)
- CRF of SITH/001921 (v 2.0)
- ICH Guidelines on Statistical Principles for Clinical Trials

2. STUDY OBJECTIVE AND DESIGN

2.1 Study Objective

The aim of this clinical investigation is to evaluate the clinical performance of the IMD for blood glucose control and its clinical safety.

2.1.1 Primary Objective

The primary objective of the study is as follows:

- To evaluate if the IMD treatment leads to a greater reduction in HbA1c level (relative to baseline) in comparison to placebo, in obese or overweight subjects with elevated blood glucose levels.

2.1.2 Main Secondary Objective

The main secondary objective of the study is as follows:

- To evaluate if the IMD treatment leads to a greater reduction in body weight (relative to baseline) in comparison to placebo.

2.1.3 Further secondary Objectives

The further secondary objectives of the study are as follows:

- To evaluate if the IMD treatment leads to a greater reduction in Homeostasis Model Assessment for Insulin Resistance (HOMA-IR), lipid levels, fasting blood insulin (FBI) and glucose (FBG) levels, sagittal abdominal diameter (SAD), waist- hip-ratio, body composition, and 12 Items Short Form Health Survey (SF-12) (all relative to baseline) in comparison to placebo.
- To evaluate the effect of the IMD treatment on body weight in comparison to placebo after treatment has ended.

2.1.4 Safety objective

The safety objective of the study is as follows:

- To assess the clinical safety of the IMD treatment in comparison to placebo.

2.1.5 Explorative objectives

The explorative objectives of the study are as follows:

- To evaluate the effect of the IMD treatment on HbA1c levels, body weight assessed on site, HOMA-IR, lipid levels, FBI, FBG levels, SAD, waist-hip-ratio, body composition in comparison to placebo.
- To evaluate the effect of the IMD treatment on Homeostasis Model Assessment for Beta Cell Function (HOMA-B), Quantitative insulin sensitivity check index (QUICKI), lipid levels, cardiometabolic risk indices, waist (WC) and hip circumference (HC) in comparison to placebo.
- To evaluate the effect of the IMD treatment on hs-CRP in comparison to placebo.
- To assess subject satisfaction and the ease of use of study treatment.
- To evaluate the microbiome diversity in stool samples.

2.2 Study Design

The present clinical investigation is a randomized, double-blinded, placebo-controlled, multicenter international study, planned to be conducted in Poland, Romania and Slovakia. The investigation will be performed to establish clinical evidence on the performance and safety of the IMD and is aiming at evaluating the suitability of the product for the intended purpose and population.

Schedule of Study

Procedure/ Assessment	Visit 1 Screening	Visit 2 Baseline Start of Treatment	Phone call 1	Visit 3 Control	Phone call 2	Visit 4 Final visit End of treatment	Phone call 3 (follow-up)	Phone call 4 (follow-up)
	Day -14 to Day -5	Day 0	Day 21 ±3 (3 weeks ±3 days after V2)	Day 42 ±3 (6 weeks ±3 days after V2)	Day 63 ±5 (9 weeks ±5 days after V2)	Day 84 ±5 (12 weeks ±5 days after V2)	7-10 days after V4	21-35 days after V4
Subject information	X							
Written informed consent, consent for data processing	X							
Inclusion and exclusion criteria	X							
Confirmation of eligibility criteria[f], randomization		X						
Anamnestic, demographic data	X							
Questioning on smoking status	X	X		X		X		
Medical history/concurrent diseases	X							
Concurrent treatment	X	X	X	X	X	X	X	X
Urine pregnancy test for women of childbearing potential	X							
Physical examination	X					X		
12 lead ECG	X							
Blood pressure, pulse rate	X	X		X		X		
Body weight on site	X	X		X		X		
Body height and BMI[e]	X							
Waist and hip circumference, SAD		X		X		X		
Body composition assessment (BIA)		X		X		X		
Fasted blood draw for HbA1c, glucose, insulin, and lipid parameters[a] (central lab)	X	X		X		X		

Procedure/ Assessment	Visit 1 Screening	Visit 2 Baseline Start of Treatment	Phone call 1	Visit 3 Control	Phone call 2	Visit 4 Final visit End of treatment	Phone call 3 (follow-up)	Phone call 4 (follow-up)
	Day -14 to Day -5	Day 0	Day 21 ±3 (3 weeks ±3 days after V2)	Day 42 ±3 (6 weeks ±3 days after V2)	Day 63 ±5 (9 weeks ±5 days after V2)	Day 84 ±5 (12 weeks ±5 days after V2)	7-10 days after V4	21-35 days after V4
Fasted blood draw for safety parameters (blood count[b], liver[c] and renal[d] parameters) (central lab)	X	X				X		
Fasted blood draw for TSH (central lab)	X							
Fasted blood draw for hs-CRP (central lab)	X	X				X		
Blood draw for analyses of vitamins/trace elements[g] (central lab)		X				X		
12 Items Short Form Health Survey (SF-12)		X				X		
Issue of study treatment and instructions on study treatment use		X		X				
Issue of subject log and scales to subject, instruction		X						
Check of subject log (weight)			X			X	X	
Issue of material and instructions for stool sample collection	X			X				
Stool sample collection[h] (central lab)		X				X		
Study treatment collection and compliance check[i]				X		X		
Adverse events	X	X	X	X	X	X	X	X
Device deficiencies			X	X	X	X		
Body weight measurement off site[j]		X				X	X	
Subject satisfaction and ease of use of study treatment[k]						X		

[a] Lipid parameters: TG, TC, LDL-C, HDL-C. VLDL-C and non-HDL-C will be calculated within the eCRF.

[b] Blood count: hemoglobin, hematocrit, erythrocytes, thrombocytes, reticulocytes, leucocytes

[c] Liver function parameters: ALAT, ASAT, Gamma-GT, alkaline phosphatase, bilirubin

[d] Renal function parameters: creatinine, urea, cystatine C, eGRF, uric acid

[e] BMI will be assessed on site at V1 and during the statistical analysis for all later time points, calculated within the eCRF based on body weight measured on site

[f] With respect to laboratory findings, only the blood draw at V1 is relevant for the enrolment/randomization

[g] Vitamins/trace elements: vitamin B12, vitamin D, Mg, Zn

[h] Two samples each time in the week before the visit; for gut microbiome parameters and storage for further putative later microbiome analyses

[I] Number of unused stick packs returned to be entered in the eCRF. Compliance will be calculated during the statistical analysis.

[j] One day after V2 and thereafter on the day of V4/PC3.

[k] Questions to be asked by site personnel and answers to be captured in the eCRF.

2.3 Statistical Hypotheses and Sample Size Justification

The primary analysis will be conducted using a two-sided test with α = 0.05 (significance level α=5%), using the following hypotheses:

- H_0: $\mu_{active} = \mu_{placebo}$, i.e., no difference between IMD treatment group and placebo control group with respect to the primary endpoint,
- H_A: $\mu_{active} \neq \mu_{placebo}$, i.e., difference between IMD treatment group and placebo control group with respect to the primary endpoint,

where H_0 is the null hypothesis and H_A is the alternative hypothesis.

μ_{active} and $\mu_{placebo}$ are,
μ_{active} = expected change in HbA1c levels at V4 (week 12) compared to V2 (baseline) for the active group treated with IMD,
$\mu_{placebo}$ = expected change in HbA1c levels at V4 (week 12) compared to V2 (baseline) for the control group treated with placebo.

A sample size of n=130 subjects in each group (the active and the control group), will have 80% power to detect a difference in means of -0,7 mmol/mol (the difference between a mean change in HbA1c in the active group and a mean change in HbA1c in the placebo group), assuming that the standard deviation in both treatment groups is 2 mmol/mol using a two-group t-test with a 5% two-sided significance level.

Considering a maximum dropout rate of about 10% of subjects not completing the study, it is therefore planned to randomize a total of 288 subjects (with n = 144 subjects per treatment group). In case of a large number of dropouts/withdrawals, randomization of additional subjects could be considered.

The choice of mean difference is based on previous trial data. In orientation to previously conducted trials, the pre/post change of HbA1c levels at V4 (week 12) compared to V2 (baseline) in the IMD treatment group is expected to be at least -0.7 mmol/mol (Maruthur et al., 2013; Baek et al., 2021). For the placebo control group, no change is presumed (0 mmol/mol). Thus, the difference in pre/post changes between the IMD treatment group and the placebo group is expected to be at least -0.7 mmol/mol.

The choice of the standard deviation in the sample size calculation is based on data from an earlier study with the IMD predecessor SiPore15®. In that study (STAR01, Baek et al., 2021; clinical investigation report, on file with the Sponsor) the observed standard deviation for the change in HbA1c over 12 weeks of treatment was 1.51 mmol/mol. It is realized that the observed standard deviation may be low in relation to what can be expected in the planned study. In order to compensate for a possible greater standard deviation in the present larger scale study, a standard deviation of 2.0 mmol/mol will be used in the sample size calculations.

With the planned required sample size of n = 130 subjects per treatment group completing the study, the table below shows the probability of observing at least one SADE (in one treatment group) when the expected probability of the SADE is 1%, 1.5%, and 2%.

	**Case 1**	**Case 2**	**Case 3**
Probability of observing at least one event	72.9%	86.0%	92.8%

Actual probability of event	1%	1.5%	2%
Number of subjects studied (n)	130	130	130

3. GENERAL ANALYSIS DEFINITIONS

3.1 Analysis Sets

The analysis sets of subjects of this clinical trial are classified into Enrolled Set (ES), Randomized Set (RS), Safety Set (SS), Full Analysis Set (FAS), and Per-Protocol Set (PPS).

The primary and main secondary analysis will be conducted on both FAS and PPS populations. The FAS is considered the main analysis while the PPS is only supportive. The analysis sets for each item are as follows.

	ES	RS	SS	FAS	PPS
Participants Status	O				
Protocol Violation and Disposition, Demographics and Baseline Characteristics		O			
Medical History, Prior and Concomitant Medications		O			
Treatment Compliance				O	
Primary Endpoint				O* (Main)	O
Main Secondary Endpoint				O* (Main)	O
Further Secondary Endpoints				O	
Exploratory Endpoints				O	
Safety			O		

*Subgroup and sensitivity analyses

The classification of each patient with respect to each analysis set will be done prior to database lock.

3.1.1 Enrolled Set (ES)

The ES includes all subjects who signed the ICF.

3.1.2 Randomized Set (RS)

The RS includes all subjects who were randomized.

3.1.3 Safety Set (SS)

The SS includes all randomized subjects who received at least 1 dose of study treatment. This meant that the subjects who have taken study treatment at least once.

3.1.4 Full Analysis Set (FAS)

The FAS includes all randomized subjects who received (have taken) at least 1 dose of study treatment and have both a baseline and at least 1 postbaseline measurement of HbA1c values.

<u>In case of Incorrect assignment of study treatment</u>

It is analyzed according to the originally randomly assigned group.

3.1.5 Per Protocol Set (PPS)

The PPS includes a subset of subjects in the FAS who completed the study without any important deviations from the Clinical Investigation Plan (CIP) and its procedures.

3.2 Clinical Investigation Plan deviations

CIP violation lists will be categorized according to the document of CIP deviations.

It will be determined whether to exclude those who violate the clinical investigation plan from the PPS by comprehensively considering whether they have affected the results of the clinical trial through data review meeting before database lock.

3.3 General Principles

Continuous variables will be summarized as number of patients (n), mean, median, standard deviation, quartile 1 and 3 and range (min, max) by visit. The change from baseline at each respective visit will also be presented. Discrete (categorical/ordinal) variables will be summarized in frequency tables (frequency and proportion) by visit and 95% confidence interval will be presented if necessary.

Graphical presentations will be used where appropriate.

Basic statistics are rounded to the second decimal place. However, the p-value is presented up to the fourth decimal place (if less than 0.0001, <0.0001) and the frequency is presented as an integer.

All safety analyses and summaries will be based on the safety analysis set. There will be no hypothesis testing for the safety data.

By-subject listings will be presented for all subjects in the Randomized Set.

When creating analysis data sets, if arithmetic operations are performed, SAS's "ROUND" function will be used to generate values up to 6 decimal places.

3.4 Stratifications

Stratification factors are HbA1c level (<48/≥48 mmol/mol; <6.5%/≥6.5%) and BMI (<30/≥ 30 kg/m^2).

3.5 Medical Dictionary and version

The medical coding and version applied to this study are as follows.

- Adverse events, Medical History, and Prior and Concomitant Treatments are classified into System Organ Class (SOC) and Preferred Term (PT) according to the latest version of MedDRA.
- Prior and Concomitant Medications are classified into Anatomical Therapeutic Chemical (ATC) Classification System Level 1 and Level 2 according to the latest version of WHODrug.

3.6 Software version

All statistical analyses will be analyzed using SAS statistical software version 9.4 or higher. If a graph is required, it will be presented using SAS/Graph or NCSS, Excel, etc.

3.7 Visit Windows

The data handling considering Visit Window in the regular visit and dropout of this clinical trial will be based on the table below.

Scheduled Visit	Time Interval (label on output)	Target Time Point	Visit window	Assessment Time Interval (Day)
Visit 1	Screening			
Visit 2	Baseline	0		0
Phone call 1	3 weeks	21	±3 days	
Visit 3	6 weeks	42	±3 days	1 to 63
Phone call 2	9 weeks	63	±5 days	
Visit 4	12 weeks	84	±5 days	64 to 89
Phone call 3	follow-up	7-10 days after V4		
Phone call 4	follow-up	21-35 days after V4		
Applied variable: HbA1c, Glucose, Insulin, Lipid parameters, Physical Measurements, hs-CRP				

If multiple assessments were taken within an analysis window, the assessment obtained on the day closest to the target day will be used; in the case of a tie, the assessment obtained on the later day will be used in the analysis.

3.8 Data Handling Rules

3.8.1 Handling of Missing data

For efficacy analysis, all collected values of regular and dropout visits will be allocated to the assessment visit according to the 'Assessment Time Interval' described in 3.7 Visit Window.

A sensitivity analysis will be conducted for the primary and main secondary endpoint where missing values will be imputed using Multiple imputation.
In the case of safety analysis, raw data will be used as collected.

Baseline is defined as the last measurement prior to the first dose of study treatment unless stated otherwise. If all measurements prior to first dose of study drug is missing, then Baseline value is set to missing.

3.8.2 Handling of Incomplete dates

If a date variable is required in the definition of items and the date variable contains a missing (UK), then the most conservative method is used to define them. For example, if the month and year of start date of an adverse event is the same as the month and year of study treatment start date, the adverse event is regarded as a treatment-emergent adverse event. It also will be described in the 'variable and/or definition' of each item.

3.8.3 Handling of Invalid Values

If the measurements of the laboratory are collected with inequalities, they are summarized as follows. When presenting a list of subjects, the collected values are presented in the EDC.

	Before	After
Upper bound (>, >=)	>5	5
	>=5	5
Lower bound (<. <=)	<5	5
	<=5	5

4. INTERIM ANALYSIS AND DATA MONITORING COMMITTEE REVIEW

Any interim analysis is not planned in this study.

5. SUBJECT INFORMATION

5.1 Disposition Information

5.1.1 Variable and/or Definition

The subjects of this clinical trial are classified as follows.

- Enrolled: All subjects who signed the ICF
- Screening Failure: Subjects who failed prior to randomization
- Randomized: Subjects who were randomized
- Treated: Randomized subjects who have taken study treatment
- Withdrawn: Randomized subjects who were withdrawn from the clinical trial
- Completed: Randomized subjects who completed the clinical trial

The reasons for those who have not completed are classified as follows.

- Screening Failure
  - Inclusion/Exclusion criteria not met
  - Consent withdrawal
  - Lost to follow-up
  - Other
- Withdrawn
  - Withdrawal of informed consent by the subject or his/her legally acceptable representative
  - Enrolled subject despite the inclusion and exclusion criteria violation is identified during the study
  - Difficult to continue the study due to an AE
  - Prohibited therapy (drug/non-drug) administered or required
  - Lost to follow-up
  - Pregnancy in a female subject

- Other reasons based on which continued study participation is difficult, in the opinion of the investigator

5.1.2 Analysis Methods

A disposition table will be presented by treatment group where applicable, and overall, the number and/or percentage of subjects who have been enrolled, screen failed prior to randomization, and who have taken treatment, discontinued and completed the clinical trial. The reasons of screening failure and withdrawn will also be summarized.

The number and percentage of subjects for each violation.

The number and percentage of randomized set, safety set, FAS, PPS will be presented.

By-subject listings of discontinued patients, clinical investigation plan deviation list, detailed of the analysis sets, screening failure (for enrolled), and visit date information will be provided.

5.2 Demographics and Baseline Characteristics

5.2.1 Variable and/or Definition

The definitions or categories of demographic characteristics of subjects are as follows.

Type	Variable Name	Unit/Category	Derivation
Continuous variable	Age	years	
	Weight	kg	Viisit 1
	Height	cm	
	BMI	kg/m^2	Auto-calculated value of EDC
Categorical variable	Gender	Male/Female	
	Childbearing Potential	Yes/No	
	Pregnancy Test	Positive / Negative	
	Ethnicity	American Indian or Alaska Native / Asian / Black or African American / Native Hawaiian or Other Pacific Islander / White / Hispanic or Latino / Other / Not Reported	
	Smoking Status	Yes/No	Visit 1

5.2.2 Analysis Methods

Baseline anthropometric and demographic characteristics will be summarized by treatment group for the subjects in the randomized set. Descriptive statistics (n, mean, standard deviation, median, quartile 1 ,3 and range) will be provided by treatment group for Continuous variables.

The number and percentage of subjects in the categorical variables will also be summarized by treatment group.

A by-subject listing of demographics and baseline characteristics will be provided.

5.3 Medical History

5.3.1 Variable and/or Definition

Past and current medical history will be classified into System Organ Class (SOC) and Preferred Term (PT) according to MedDRA coding.

- Past medical history: It is defined as the case where 'Ongoing at Screening' is checked as 'No'.
- Current medical history: It is defined as the case where 'Ongoing at Screening' is checked as 'Yes'.

5.3.2 Analysis Methods

Number and percentage of subjects and number of events will be summarized by system organ class (SOC) and preferred term (PT).

A by-subject listing of medical history will be provided.

5.4 Prior and Concomitant Medications

5.4.1 Variable and/or Definition

Medications will be classified as follows.

- Prior medications: It is defined as medications that has been taken before the study treatment start date. If the end date of administration of the medications is the same as the study treatment start date, it is considered as concomitant medications.
- Concomitant medications: It is defined as medications taken on and after the study treatment start date. If it is difficult to determine whether it is a prior or concomitant medication due to "UK" in dates, it is considered a concomitant medication.

Prior and Concomitant Medications will be classified into Level 1 and Level 2 of ATC code.

5.4.2 Analysis Methods

Number and percentage of subjects and number of events will be summarized by Level 1 and Level 2 of ATC code.

A by-subject listing of prior and concomitant medication data will be provided.

5.5 Prior and Concomitant Treatments

5.5.1 Variable and/or Definition

- Prior treatments: It is defined as treatments that has been taken before the study treatment start date. If the end date of treatments is the same as the study treatment start date, it is considered as concomitant treatments.
- Concomitant treatments: It is defined as treatments taken on and after the study treatment start date. If it is difficult to determine whether it is a prior or concomitant treatment due to "UK" in dates, it is considered a concomitant treatment.

Prior and Concomitant treatments will be classified into SOC and PT.

5.5.2 Analysis Methods

Number and percentage of subjects and number of events will be summarized by SOC and PT.

A by-subject listing of prior and concomitant treatment data will be provided.

5.6 Treatment Compliance

5.6.1 Variable and/or Definition

(1) Identification and Description of the Investigational Medical Device and Placebo

Investigational Medical Device (IMD): SiPore21®

Placebo: comparable to the IMD in appearance, texture, taste, and smell

(2) Daily dose and administration method, time of administration

- During the treatment period of 12 weeks, the IMD/placebo should be taken 3 times a day.
- It should be taken with the 3 largest meals of the day (e.g., breakfast, lunch, dinner). It should be taken in direct connection with the first bite of each respective meal, preferably after having chewed (if solid food) and swallowed the first bite of the meal.

(3) Total duration of administration

- Total duration of administration(day) = Study treatment End Date* – Study treatment Start Date

  *Study treatment End Date is regarded as 'Date of Completion/Withdrawal' of 'End of Study' page in the CRF if it is missing.

(4) Treatment Compliance

$$\text{Treatment Compliance}(\%) = \frac{\text{Number of actual stick packs taken}}{\text{Number of expected stick packs taken}} \times 100$$

- Number of actual stick packs taken = Sum of 'Dispensed' – 'Returned'(unsued) collected in the CRF (Visit 2 ~ Visit 4)
- Number of expected stick packs taken = (Last on-site visit* - Randomization Date - 1) X 3

  *For subject who withdrew, the last on-site visit is regarded as 'Date of Completion/Withdrawal' of 'End of Study' page in the CRF.

Percent compliance to the study treatment will be summarized by treatment group based on FAS.

5.6.2 Analysis Methods

Number of patients (n), mean, median, standard deviation, quartile 1 and 3 and range (min, max) for each group will be presented for total duration of administration and treatment compliance.

A by-subject listing of treatment compliance will be provided.

6. EFFICACY

All tests will be two-sided and performed at the 5% significance level. When reporting the results of significance tests, p-values for the two-sided test will be reported together with the corresponding 2-sided symmetric 95% confidence interval.

6.1 Primary Efficacy Endpoint

Difference in changes in HbA1c levels from V2 (baseline) to V4 (week 12) between IMD treatment group and placebo group.

6.1.1 Variable and/or Definition

The primary endpoint is defined as the difference in changes in HbA1c levels from V2 (baseline) to V4 (week 12) between IMD treatment group and placebo group. A higher reduction of HbA1c (related to baseline) in comparison to placebo corresponds to a better clinical performance.

The primary analysis will be conducted on both FAS and PPS populations. The FAS is considered the main analysis while the PPS is only supportive.

HbA1c is one of the measurements of Laboratory Tests (Central Lab).

- Changes in HbA1c (mmol/mol) = Week 12 (Visit 4) HbA1c – Baseline (Visit 2) HbA1c

6.1.2 Analysis Methods

The analysis will be conducted using an ANCOVA model. The model will include baseline HbA1c at V2 as covariate and treatment group, BMI at V1 (<30 or ≥ 30 kg/m^2), HbA1c level at V1 (<48 or ≥48 mmol/mol) as factors. The two-sided p-value will be considered statistically significant if it is below 5%.

If one of the assumptions of the ANCOVA listed below is violated, the non-parametric analysis Wilcoxon rank sum test will also be performed as a supportive analysis.

1. Kolmogorov–Smirnov test will be applied to the residuals resulting from an ANCOVA. If the p-value is < 0.05, then normality assumption is regarded to be violated.
2. Homogeneity of variances will be checked based on Levene's test of Procedure GLM. If the p-value is < 0.05, then the assumption is regarded to be violated.

A sensitivity analysis will be conducted for the primary endpoint where missing values will be imputed using multiple imputation.
For the multiple imputation, the SAS procedures MI will be used. Derived variables will not be imputed but will be derived from imputed values (e.g., if the HbA1c value at V4 (week 12) is missing, the HbA1c value at V4 (week 12) will be imputed to calculate the change in HbA1c levels at V4 (week 12) compared to V2 (baseline)). The seed number will be set to the date of unblinding (YYYYMMDD; for example, if the unblinding date is January 1, 2021, it would be 20210101) and the number of imputations will be set to 100. The Fully Conditional Specification (FCS) regression method will be used to impute values for continuous data with an arbitrary missing data pattern.
Covariates that are considered for inclusion in the multiple imputation model for better prediction of the missing values are study treatment, variables used for stratified randomization and HbA1c values at all points in time from V2 (baseline) to V4 (week 12)
ANCOVA will then be performed for each of the relevant MI datasets. The results of the 100 analyses will be combined into a single analysis using PROC MIANALYZE.

Subgroup analyses will be performed on FAS for subgroups BMI at V1 (<30 or ≥ 30 kg/m^2), HbA1c level at V1 (<48 or ≥48 mmol/mol).

A by-subject listing of primary efficacy response data will be provided.

6.2 Main Secondary Efficacy Endpoint

Difference in changes in body weight assessed on-site from V2 (baseline) to V4 (week 12) between IMD treatment group and placebo group.

6.2.1 Variable and/or Definition

Body weight is one of the Physical Measurements and will be collected as 'Weight(kg)' in the Physical Measurements of CRF.

- Changes in body weight assessed on-site (kg) = Week 12 (Visit 4) weight – Baseline (Visit 2) weight

6.2.2 Analysis Methods

All statistical analyses proposed in the CIP and the SAP will be reported in the CIR, regardless if the primary endpoint reaches significance. The analysis will be conducted using an ANCOVA model. The model will include body weight value at V2 as covariate and treatment group, BMI at V1 (<30 or ≥ 30 kg/m^2), HbA1c level at V1 (<48 or ≥48 mmol/mol) as factors.

If one of the assumptions of the ANCOVA is clearly violated, the non-parametric analysis Wilcoxon rank sum test will also be performed as a supportive analysis.

A sensitivity analysis will be conducted where missing data are imputed based on multiple imputations using the same methods as for the primary efficacy sensitivity analysis.

Subgroup analyses will be performed on FAS for subgroups BMI at V1 (<30 or ≥ 30 kg/m^2), HbA1c level at V1 (<48 or ≥48 mmol/mol).

A by-subject listing of main secondary efficacy response data will be provided.

6.3 Further Secondary Efficacy Endpoints

Difference between IMD treatment group and placebo group in changes from V2 (baseline) to V4 (week 12) in:

- HOMA-IR
- Total Cholesterol (TC)
- Fasting Blood Insulin (FBI)
- Fasting Plasma Glucose (FBG)
- LDL-C
- Sagittal abdominal diameter (SAD)
- Triglycerides (TG)
- Waist-hip-ratio
- Body fat content/mass, fat free mass (assessed by BIA)
- SF-12

Difference between IMD treatment group and placebo group in changes in body weight assessed off site from V2 (baseline) to PC1 (week 3) and to V4 (week 12) and to PC3 (week 13)

6.3.1 Variable and/or Definition

Further secondary efficacy endpoints will be collected in the CRF as below.

- HOMA-IR (mmol/l) = FBI(='Insulin') (mU/l*) X FBG(='Glucose') (mmol/l*) / 22.5
  *where 1 mU/l = 1 uU/ml, 1 mg/dL = 0.0555 mmol/L.
- 'TC' (mg/dL) in the Laboratory Tests (Central Lab) of CRF
- FBI is collected as 'Insulin' (uU/ml) in the Laboratory Tests (Central Lab) of CRF.
- FBG is collected as 'Glucose' (mg/dL) in the Laboratory Tests (Central Lab) of CRF.
- 'LDL-C' (mg/dL) in the Laboratory Tests (Central Lab) of CRF
- 'SAD' (cm) in the Physical Measurements of CRF
- 'TG' (mg/dL) in the Laboratory Tests (Central Lab) of CRF
- 'Waist-hip-ratio' [auto-calculated] in the Physical Measurements of CRF
- 'Body fat content/mass' (kg) in the Physical Measurements of CRF
- 'Fat free mass' (kg) in the Physical Measurements of CRF
- SF-12 total score: FCS(Physical Component Summary), MCS(Mental Component Summary)

  - Changes in each endpoint = Week 12 (Visit 4) – Baseline (Visit 2)

Body weight assessed off site will be collected as 'Body weight (off site)'.

  - Changes in body weight assessed off site (kg) = (Week 3 (PC1) weight or Week 12 (Visit 4) weight or Week 13 (PC3) weight) – Baseline (Visit 2) weight (on-site)

6.3.2 Analysis Methods

The statistical analysis will be an ANCOVA analysis such as primary and main secondary efficacy analysis based on FAS. If one of the assumptions of the ANCOVA is clearly violated, the non-parametric analysis Wilcoxon rank sum test will also be performed as a supportive analysis.

By-subject listings of further secondary efficacy response data will be provided.

6.4 Exploratory Efficacy Endpoints

- Difference between IMD treatment group and placebo group in changes from V2 (baseline) to V3 (week 6) in:

  - HbA1c levels
  - Body weight assessed on site
  - HOMA-IR
  - TC
  - FBI
  - FBG
  - LDL-C
  - SAD
  - TG
  - Waist-hip-ratio

- Body fat content/mass, fat free mass (assessed by BIA)

- Difference between IMD treatment group and placebo group in changes from V2 (baseline) to V3 (week 6) and to V4 (week 12) in:
  - HOMA-B, Quantitative Insulin Sensitivity Check Index (QUICKI)
  - Very Low-Density Lipoprotein Cholesterol (VLDL-C), HDL-C, non-HDL-C
  - Atherogenic index of plasma (AIP), atherogenic coefficient (AC) and cardiac risk ratio (CRR) 1 and 2
  - Waist Circumference (WC) and Hip Circumference (HC)
  - Body Mass Index (BMI)
- Difference between IMD treatment group and placebo group in changes from V2 (baseline) to V4 (week 12) in hs-CRP levels
- Assessment of subject satisfaction and ease of use of study treatment at V4 (week 12)
- Explore the differences in intestinal flora composition at V2 (baseline) and V4 (week 12) and content between IMD treatment group and placebo group

6.4.1 Variable and/or Definition

HbA1c levels, Body weight assessed on site, HOMA-IR, TC, FBI, FBG, LDL-C, SAD, TG, Waist-hip-ratio, Body fat content/mass and fat free mass are defined in '6.1 Primary Efficacy End point', '6.2 Main Secondary Efficacy End point' and '6.3 Further Secondary Efficacy End point'.

- Changes in HbA1c levels, Body weight assessed on site, HOMA-IR, TC, FBI, FBG, LDL-C, SAD, TG, Waist-hip-ratio, Body fat content/mass, and fat free mass = Week 6 (Visit 3) – Baseline (Visit 2)

HOMA-B, QUICKI, VLDL-C, HDL-C, non-HDL-C, AIP, AC, CRR 1, CRR2, WC, HC and BMI will be collected in the CRF as below.

- HOMA-B = 20 X FBI(='Insulin') (mU/l*) / (FBG(='Glucose') (mmol/l*) – 3.5)
- QUICKI = 1/[log10(FBI(='Insulin') (mU/l*)) + log10(FBG(='Glucose') (md/dL))]
  *where 1 mU/l = 1 uU/ml, 1 mg/dL = 0.0555 mmol/L.
- 'VLDL-C' [auto-calculated] (mg/dL) in the Laboratory Tests (Central Lab) of CRF
- 'HDL-C' (mg/dL) in the Laboratory Tests (Central Lab) of CRF
- 'non-HDL-C' [auto-calculated] (mg/dL) in the Laboratory Tests (Central Lab) of CRF
- Atherogenic index of plasma (AIP) = log10 ('TG'/'HDL-C') in the Laboratory Tests (Central Lab) of CRF
- atherogenic coefficient (AC) = ('TC'- 'HDL-C')/'HDL-C' in the Laboratory Tests (Central Lab) of CRF
- Cardiac risk ratio (CRR) 1 = 'TC'/'HDL-C' in the Laboratory Tests (Central Lab) of CRF
- Cardiac risk ratio (CRR) 2 = 'LDL-C'/'HDL-C' in the Laboratory Tests (Central Lab) of CRF
- WC is collected as 'Waist circumference' (cm) in the Physical Measurements of CRF.
- HC is collected as 'Hip circumference' (cm) in the Physical Measurements of CRF.
- BMI = 'Weight' (kg) in the Physical Measurements of CRF / (Height (cm) at V1 / 100)^2

  - Changes in HOMA-B, QUICKI, VLDL-C, HDL-C, non-HDL-C, AIP, AC, CRR 1, CRR2, WC, HC

and BMI = (Week 6 (Visit 3) or Week 12 (Visit 4)) – Baseline (Visit 2)

hs-CRP is one of the measurements of Laboratory Tests (Central Lab).

- Changes in hs-CRP (mg/l) = Week 12 (Visit 4) hs-CRP – Baseline (Visit 2) hs-CRP

Subject satisfaction consists of 6 questions in the CRF.

- Is the product easy to use? (very easy/easy/moderately easy/difficult)
- Is the instruction for use easy to follow? (very easy/easy/moderately easy/difficult)
- Do you feel safe using this product? (yes/no)
- Are you satisfied with this product? (yes/no)
- Would you recommend this product to your prediabetic/diabetic friend? (yes/no)
- Has your quality of life improved since you started using this product? (yes/no)

The frozen stool samples will not be analyzed, so no analysis of intestinal flora composition will be conducted, and the stool sample analysis results will not be included in the Clinical Study Report (CSR).

6.4.2 Analysis Methods

All exploratory endpoints will be analyzed in a non-hierarchical procedure using FAS.

The statistical analysis will be an ANCOVA analysis such as primary and main secondary efficacy analysis based on FAS. If one of the assumptions of the ANCOVA is clearly violated, the non-parametric analysis Wilcoxon rank sum test will also be performed as a supportive analysis.

But frequency and proportion for each group will be presented for subject satisfaction and ease of use. For this, the Chi-square test will be performed, or Fisher's exact test if cells with an expected frequency of less than 5 are more than 20% of all cells.

By-subject listings of exploratory efficacy response data will be provided.

7. SAFETY

1) Adverse Events
2) Device Deficiencies
3) Laboratory Tests (Central Lab)
4) Vital Signs
5) Vitamins and Minerals

Patients will be analyzed according to the actual treatment they have received.

7.1 Adverse Events

7.1.1 Variable and/or Definition

All reported adverse events with onset during the treatment phase (i.e., treatment-emergent AEs) will be included in the analysis and are defined as follows.

- Treatment-emergent AEs: All reported adverse events with onset during the treatment phase. If the start date of adverse event is the same as the study treatment start date, it is considered as a Treatment-emergent AE. If it is difficult to determine whether it is a Treatment-emergent AE due to "UK" in dates, it is considered a Treatment-emergent AE.

- Adverse Device Effects (ADEs): Treatment-emergent AEs with a causal relationship to study treatment (including those rated as possibly or probably related)
- Serious Adverse Events (SAEs): Serious Treatment-emergent AEs
- Serious Adverse Device Effects (SADEs)
- Treatment-emergent AEs that caused study withdrawal: Treatment-emergent AEs in which the reason for withdrawal is "Difficult to continue the study due to an AE" and the actions taken for IMD is "Discontinued".

All Treatment-emergent AEs will be classified into System Organ Class (SOC) and Preferred Term (PT) according to MedDRA coding.

The categories of Outcome, Severity, Causality, Actions Taken for IMD, Other Actions Taken for adverse event evaluation are as follows:

- Outcome: Resolved without sequelae, Resolved with sequelae, Ongoing, Fatal, Unknown
- Severity: Mild, Moderate, Severe
- Causality: Not related, Possible, Probable, Causal relationship
- Actions Taken for IMD: No dose change, Dose increased, Dose decreased, Discontinued, Unknown, Not applicable
- Other Actions Taken: Drug therapy, Non-drug therapy, Drug and non-drug therapy, No treatment given

7.1.2 Analysis Methods

For all Treatment-emergent AEs, ADEs, SAEs, and Treatment-emergent AEs that caused study withdrawal, number and percentage of subjects, 95% two-sided confidence interval (Clopper-Pearson exact method) of the percentage, and number of events will be presented by treatment group. Those will also be summarized by SOC and PT.

The types of Treatment-emergent AEs for each treatment group will be classified according to outcome, severity, causality, Actions Taken for IMD, and Other Actions Taken, and corrective treatment, and number and percentage of subjects, and number of events will be summarized by SOC, PT, and treatment group.

By-subject listings of adverse event listings, subjects with serious adverse events, and subjects withdrawn due to adverse events will be provided.

7.2 Device Deficiencies (DDs)

7.2.1 Variable and/or Definition

Device Deficiencies are defined as follows.

- DDs: All reported Device Deficiencies
- DDs with SADE

The categories of Action Taken with Study Device are as follows:

- Action Taken with Study Device: No Change, Device Modified/Adjusted, Device Replaced, Removed Temporarily, Removed, Unknown

7.2.2 Analysis Methods

For Device Deficiencies and Device Deficiencies with SADE, number and percentage of subjects, 95% two-sided confidence interval (exact method) of the percentage, and number of events will be presented by treatment group.

A by-subject listing of further secondary efficacy response data will be provided.

7.3 Laboratory Tests

7.3.1 Variable and/or Definition

Safety parameters of laboratory tests (central lab) are classified as follows.

Full blood count parameters	Hemoglobin, Hematocrit, Erythrocytes, Thrombocytes, Reticulocytes, Leucocytes
Liver function parameters	ALAT, ASAT, Gamma-GT, Alkaline Phosphatase, Bilirubin
Renal function parameters	Creatinine, Urea, Cystatine C, Uric acid, eGRF

Venous blood samples will be drawn at Visit 1, Visit 2, Visit 3, and Visit 4.

Laboratory test results are shown as normal and abnormal, and abnormalities are divided into NCS and CS. Changes from baseline (Visit 2) to week 12 (Visit 4) will be calculated as follow.

- Change from Baseline to Week 12 = Result at Week 12 – Result at Baseline (Visit 2)

7.3.2 Analysis Methods

Descriptive statistics of each parameter will be presented by each time point and treatment group. Change from baseline (Visit 2) to week 12 (Visit 4) will also be presented.

For Normality of laboratory test, the changes after IMD Treatment compared to before treatment (Visit 2) will be classified into 'normal/abnormal, not clinically significant (NCS)' or 'abnormal, clinically significant (CS)', and presented in a shift table.

By-subject listings of individual laboratory measurements by patient will be provided.

7.4 Vital Signs

7.4.1 Variable and/or Definition

Sitting blood pressure and pulse rate will be measured at Visit 1, Visit 2 (Baseline), Visit 3 (week 6), and Visit 4 (week 12).

- Systolic/Diastolic Blood Pressure (mmHg)
- Pulse Rate (beats/min)

Changes from baseline (Visit 2) to each time point will be calculated as follow.

- Change from Baseline to week n = Result at week n – Result at Baseline (Visit 2), n=6 or 12

7.4.2 Analysis Methods

Descriptive statistics of sitting blood pressure and pulse rate will be presented by each time point and treatment group. Change from baseline (Visit 2) to each time point will also be presented.

A by-subject listing of individual vital signs by patient will be provided.

7.5 Vitamins and Trace Elements

7.5.1 Variable and/or Definition

Venous blood samples will be collected after overnight fasting for analysis of vitamins (B12, D) and trace elements (Mg, Zn) at Visit 1, Visit 2, Visit 3, and Visit 4.

Changes from baseline (Visit 2) to week 12 (Visit 4) will be calculated as follow.

- Change from Baseline to Week 12 = Result at Week 12 – Result at Baseline (Visit 2)

7.5.2 Analysis Methods

Descriptive statistics of vitamins (B12, D) and trace elements (Mg, Zn) will be presented by each time point and treatment group. Change from baseline (Visit 2) to week 12 (Visit 4) will also be presented.

A by-subject listing of individual Vitamins and trace elements by patient will be provided.

8. CHANGE IN STATISTICAL ANALYSIS METHODS

Protocol Section No.	Before (Protocol)	After (SAP)	Reason
9.9.5 Primary Endpoint & 9.9.6 Main Secondary Endpoint	The model will include baseline HbA1c at V2 as covariate and treatment group, BMI at V1 (<30 or ≥ 30 kg/m2), HbA1c level at V1 (<48 or ≥48 mmol/mol) ~~and site~~ as factors.	The model will include baseline HbA1c at V2 as covariate and treatment group, BMI at V1 (<30 or ≥ 30 kg/m2), HbA1c level at V1 (<48 or ≥48 mmol/mol) as factors.	Since the study is conducted at many sites, where some might enroll very few subjects, it was decided that the statistical models should not adjust for site. This change was done to make sure that estimates from the models are more robust and that the risk of convergency issues is minimized. The effect of this change on the estimated treatment difference is expected to be very small, especially because the endpoint is a laboratory endpoint analyzed at a central vendor.
9.9.10 Treatment Compliance	The number of expected stick packs taken is equal to 12*7*3, for 12 weeks of treatment with 3 stick packs per day.	• Number of expected stick packs taken = (Last on-site visit* - Randomization Date - 1) X 3<br>*For subject who withdrew, the last on-site visit is regarded as 'Date of Completion/Withdrawal' of 'End of Study' page in the CRF.	To calculate based on the actual visit dates for each subject.
9.9.13 Sensitivity Analyses	Sensitivity analyses might be added to the SAP, for example, addressing a large number of drop-outs. A sensitivity analysis will be conducted for the primary endpoint where missing values will be imputed. The type of	6.2.2 Analysis Methods<br><br>A sensitivity analysis will be conducted according to the primary efficacy sensitivity analysis.	This analysis was added to the main secondary efficacy endpoint change in body weight to address the effect on a potential different drop-out rate between the treatments. A similar

	imputation will be specified in the SAP.		analysis has already been proposed for the primary endpoint.

9. REFERENCES

1. Baek J, Robert-Nicoud G, Herrera Hidalgo C, Borg ML, Iqbal MN, Berlin R, Lindgren M, Waara E, Uddén A, Pietiläinen K, Bengtsson T. Engineered mesoporous silica reduces long-term blood glucose, HbA1c, and improves metabolic parameters in prediabetics. Nanomedicine (Lond). 2022;17(1):9-22.
2. Maruthur NM, Ma Y, Delahanty LM, Nelson JA, Aroda V, White NH, Marrero D, Brancati FL, Clark JM; Diabetes Prevention Program Research Group. Early response to preventive strategies in the Diabetes Prevention Program. J Gen Intern Med. 2013;28(12):1629-36

Appendix. TLF Shells

TLF Shells for the presentation of Tables, Listings, and Figures of this study are separately attached.

Signature Page

**Task**	Write
**Company**	CRScube
**Title**	Project Biostatistician
**Name**	Daehan Kim
**E-Mail**	dhkim01@crscube.co.kr
**Date**	2024-11-26 10:30 (UTC+09:00)
**Signature**	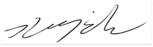

**Task**	Review
**Company**	CRScube
**Title**	STAT Group Manger
**Name**	NaYoon Chang
**E-Mail**	nychang@crscube.co.kr
**Date**	2024-11-26 10:52 (UTC+09:00)
**Signature**	NaYoon Chang

**Task**	Review
**Company**	SDS Life Science
**Title**	Senior Consultant Biostatistics
**Name**	Daniel Bruce
**E-Mail**	Daniel.Bruce@sdslifescience.com
**Date**	2024-11-26 16:23 (UTC+09:00)
**Signature**	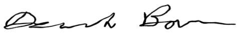

**Task**	Approve
**Company**	Sigrid Therapeutics AB
**Title**	Project Manager
**Name**	Maria Klockare
**E-Mail**	maria@sigridthx.com
**Date**	2024-11-26 17:21 (UTC+09:00)
**Signature**	Maria Klockare

**Task**	Approve
**Company**	HungaroTrial
**Title**	Project Manager
**Name**	Matyas Petho
**E-Mail**	MPetho@Hungarotrial.com
**Date**	2024-11-26 20:12 (UTC+09:00)
**Signature**	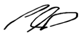
